# Comprehensive evaluation of methods for differential expression analysis of metatranscriptomics data

**DOI:** 10.1093/bib/bbad279

**Published:** 2023-08-09

**Authors:** Hunyong Cho, Yixiang Qu, Chuwen Liu, Boyang Tang, Ruiqi Lyu, Bridget M Lin, Jeffrey Roach, M Andrea Azcarate-Peril, Apoena Aguiar Ribeiro, Michael I Love, Kimon Divaris, Di Wu

**Affiliations:** Department of Biostatistics, University of North Carolina, Chapel Hill, NC, United States; Department of Biostatistics, University of North Carolina, Chapel Hill, NC, United States; Department of Biostatistics, University of North Carolina, Chapel Hill, NC, United States; Department of Statistics, University of Connecticut, Storrs, CT, United States; School of Computer Science, Carnegie Mellon University, Pittsburgh, Pennsylvania, United States; Department of Biostatistics, University of North Carolina, Chapel Hill, NC, United States; Research Computing, University of North Carolina, Chapel Hill, NC, United States; Department of Medicine and Nutrition, University of North Carolina, Chapel Hill, NC, United States; Division of Diagnostic Sciences, University of North Carolina, Chapel Hill, NC, United States; Department of Biostatistics, University of North Carolina, Chapel Hill, NC, United States; Department of Genetics, University of North Carolina, Chapel Hill, NC, United States; Division of Pediatric and Public Health, University of North Carolina, Chapel Hill, NC, United States; Department of Epidemiology, University of North Carolina, Chapel Hill, NC, United States; Department of Biostatistics, University of North Carolina, Chapel Hill, NC, United States; Division of Oral and Craniofacial Health Sciences, Adam School of Dentistry, University of North Carolina, Chapel Hill, NC, United States; Lineberger Comprehensive Cancer Center, University of North Carolina, Chapel Hill, NC, United States

**Keywords:** metatranscriptomics, metagenomics, differential expression, benchmark, logistic-beta, early childhood caries

## Abstract

Understanding the function of the human microbiome is important but the development of statistical methods specifically for the microbial gene expression (i.e. metatranscriptomics) is in its infancy. Many currently employed differential expression analysis methods have been designed for different data types and have not been evaluated in metatranscriptomics settings. To address this gap, we undertook a comprehensive evaluation and benchmarking of 10 differential analysis methods for metatranscriptomics data. We used a combination of real and simulated data to evaluate performance (i.e. type I error, false discovery rate and sensitivity) of the following methods: log-normal (LN), logistic-beta (LB), MAST, DESeq2, metagenomeSeq, ANCOM-BC, LEfSe, ALDEx2, Kruskal–Wallis and two-part Kruskal–Wallis. The simulation was informed by supragingival biofilm microbiome data from 300 preschool-age children enrolled in a study of childhood dental disease (early childhood caries, ECC), whereas validations were sought in two additional datasets from the ECC study and an inflammatory bowel disease study. The LB test showed the highest sensitivity in both small and large samples and reasonably controlled type I error. Contrarily, MAST was hampered by inflated type I error. Upon application of the LN and LB tests in the ECC study, we found that genes C8PHV7 and C8PEV7, harbored by the lactate-producing *Campylobacter gracilis*, had the strongest association with childhood dental disease. This comprehensive model evaluation offers practical guidance for selection of appropriate methods for rigorous analyses of differential expression in metatranscriptomics. Selection of an optimal method increases the possibility of detecting true signals while minimizing the chance of claiming false ones.

## INTRODUCTION

The human microbiome has emerged as an undeniable cornerstone for a multitude of health and disease outcomes [1–4]. Compared with 16S ribosomal RNA (16S rRNA) sequencing, metagenomic analysis of whole-genome shotgun sequencing (WGS) provides better phylogenetic resolution and information on microbial genomic content. In addition, metatranscriptomic analysis of total RNA sequencing provides information pertaining to microbialfunctional activity [5]. Microbial gene expression and metabolism represent viable and active members of the microbial community and reflect the biology underlying the microbiome’s interactions with the host and the environment [6–11]. Metatranscriptomic data reflect microbial communities’ functional gene activity which is essential to investigate as (1) the same genes across different species may have similar functions, and (2) many genes may be either unique to a species, or are map to as yet unclassified species. Despite the increasing significance and availability of metatranscriptomic sequencing data, the development of tailored statistical analysis methods has remained comparatively underdeveloped. Fundamentally, metagenomic and metatranscriptomic analyses differ from the analysis of single organism sequencing in that the abundance and identity of the underlying organisms being sequenced are unknown a priori and may differ extensively by sample. This makes the data compositional and sparse. Metatranscriptomic sequencing analysis is further complicated by the ambiguity of expression and abundance in the observations.

As with single organism bulk RNA sequencing and single-cell RNA sequencing (scRNAseq), one of the fundamental key analyses applied to metatranscriptomics data is differential expression (DE) analysis. DE analysis compares the expression level of taxa or microbial genes (or gene-families) between groups or conditions, e.g. different health/disease conditions, before and after treatment, or host developmental stages. It typically identifies genes whose expression levels are significantly associated with the outcomes of interest after controlling for nuisance factors such as batch or block information and host characteristics (e.g. demographics). Most currently available methods to analyze metatranscriptomic data rely on either the joint mapping of microbial DNA sequencing and RNA sequencing data [11–13] or analytic pipelines employed in other high-throughput sequencing technologies—e.g. single organism RNA sequencing—or were developed for 16S or WGS metagenomic data [14–16]. Unfortunately, very few methods have been specifically developed for metatranscriptomic DE analysis [17–24]. Although metatranscriptomic data distributions are regarded as of the same class as metagenomic data: either count, normalized count or proportion with a zero mass, metatranscriptomic data are more sparse, containing more zeros. This higher sparsity is a likely result of some taxa, genes or gene-families either not being expressed actively or not measured with sufficient sensitivity. It follows that a systematic evaluation of existing methods’ performance is warranted before they can be recommended for metatranscriptomic data analysis.

There have been attempts to evaluate differential-abundance (DA) or DE methods at the species- or the taxon-level that have included many of the commonly used preprocessing and differential abundance analysis approaches [23, 25, 26]. However, those studies neither consider the recently developed statistical models that were specifically designed for zero-inflated over-dispersed counts or compositional data such as Model-based Analysis of Single-cell Transcriptomics (MAST) [27] and logistic-beta test [28], nor evaluate methods’ performance at the gene or gene-family level in metatranscriptomics [15, 23, 25]. Recent large-scale evaluations of microbiome DA methods involve highly flexible methods accommodating high zero proportions in microbiome data but still analyze metagenomics data at a species- or taxon-level [15, 20]. Gene or gene-family level of data have much higher dimensions and are more sparse than the species- or the taxon-level of data. Due to the fact that counts of a conserved gene may be shared by many taxa, we used marginal level of gene data—aggregating abundance or expression across species (instead of analyzing each gene per species, so-called ‘joint analysis’)—as the main focus in this paper. Also, due to the fact that some different gene names may still have very similar sequence and can be hard to be discriminated, we use the gene-family level instead of the gene level of data for individual DE tests. In this paper, we simply refer to the microbial *‘gene-family’* as *‘gene’*.

In this paper, we present the results of a comparative evaluation of 10 statistical analysis methods that could be used for gene-wise microbial DE analyses in metatranscriptomics. Challenges in the methods’ evaluation include the high dimensionality of the metatranscriptomic data (e.g. millions of genes), sparse and skewed data distributions and complicated correlation structures among genes and species. To address these challenges when evaluating methods in terms of false positive rate and sensitivity, we employed two unique simulation schemes to carefully reflect the distributional characteristics of metatranscriptomic data based on existing oral and gut microbial datasets. To understand the behavior of statistical association tests clearly, one is to simulate data from fully parametric models using the parameters estimated from real data. To better reflect the complex nature of real data, we also use a semi-parametric model via random sampling of genes or permutation of samples. To ensure the similarity between the original and parametric-simulated metatranscriptomic data, we assessed the goodness of fit of several statistical distribution to the real data (Section 3.2). Our simulations are more relevant than those of Weiss *et al*. [23], wherein most of the methods being compared do not reflect the higher sparsity of metatranscriptomics data than the metagenomics data.

We used metatranscriptomic datasets from two body sites for both simulation and application. Two of the datasets are from a study of early childhood oral health called ‘Zero-Out Early Childhood Caries (ZOE)’ [29, 30] wherein oral microbial metatranscriptomic data from over 400 children are analyzed to identify association with childhood dental disease (i.e. early childhood caries, ECC). The third dataset was used to study the association between gut microbiome and inflammatory bowel diseases (‘the IBD study’, [7]). In real-data applications, we sought to identify microbial gene-families whose expression is significantly associated with ECC or IBD. While the focus of our work is gene-wise DE methods’ evaluation, to augment the reach of this comparative evaluation we provide additional results from analyses at the taxon and pathway level in the supplement.

## MATERIAL AND METHODS

### DE analysis methods

The 10 DE analysis methods evaluated in this simulation study are Log-normal test (LN), Logistic Beta test (LB) [28], MAST [27], DESeq2 [31], DESeq2-ZINBWaVE (DESeq2ZI) [32], metagenomeSeq (MGS) [25], ANCOM-BC [33], Linear discriminant analysis Effect Size (LEfSe)[34], ANOVA-Like Differential Expression analysis (ALDEx2)[35], Kruskal–Wallis test (KW) and two-part Kruskal–Wallis test (KW-II) [36]. We selected these 10 DE methods previously used in bulk RNAseq, microbiome data and scRNAseq data analyses. They were selected based on the extent of their usage in the literature and the adequacy of their distributional assumptions given the sparsity of microbial data. Some methods previously applied/developed in microbiome data or scRNaseq data usually either use ranks (KW, KW-II, LEfSe and maybe ANCOM-BC) or models that can handle excess-zero (LB, KW-II, DEseq2ZI, MAST and MGS). LN and DESeq2 were developed for bulk RNAseq but are occasionally used in microbiome data analysis. LEfSe, ALDEx2, MGS and ANCOM-BC were developed for microbiome data. The general statistical methods LN, LB and KW have been used in microbiome. The methods MAST and DEseq2ZI were developed for scRNAseq data. Most methods are tailored so that they can control batch effects (including KW and KW-II) while testing associations between gene expression and phenotypes of interest. DESeq2-ZINBWaVE is an extension of DESeq2 and ZINB-WaVE [37] to allow excess zeros specifically for DE analysis in scRNAseq analyses. ANCOM-BC2 is a variation of ANCOM [38] that inherits the philosophy of differential ranking (DR) methods and ANCOM but also provides *P*-values. The functions in ANCOM-BC2 were used for simulations, to represent ANCOM-BC. LEfSe was included due to its popularity; however, it does not provide information regarding statistical significance. Details for all these evaluated methods are presented in Supplementary Section S1. For reliability of tests or quality control, before each test, genes are screened out if they are expressed in only a few participants (the smaller of n=10 or 2% of samples).

To properly evaluate DE analysis methods, it is important to carefully consider aspects of data scaling and transformation. To this end, we consider the three widely used methods [(reads-per-kilobase (RPK), transcript-per-kilobase-million (TPM) and arcsine transformation] that provide good distributional results in the example data (Section S1).

### Description of the three metatranscriptomics datasets

Distributional characteristics of the three aforementioned metatranscriptomics datasets are presented in the Supplement (Section S2). In the first two datasets (namely, ZOE2.0 with 297 subjects and ZOE-pilot with 116 subjects) [29, 30] the association between the supragingival oral microbiome and the prevalence of clinically determined ECC [39, 40] is investigated. ECC prevalence was similar in the two ZOE waves, i.e. ZOE2.0: 49% (147/297) and ZOE-pilot: 50% (58/116). RPK data were transformed to TPM and arcsine as shown in Section 3.3. The total number of gene-species combinations (wherein counts are recorded per gene and species) in the ZOE2.0 metatranscriptome is 535 299; there are 204 distinct species and 402 937 distinct genes. In the ZOE-pilot sample, there are 439 872 gene-species, 185 distinct species and 342 004 distinct genes. Total RPKs per sample are on average 13 053 428 in ZOE2.0 and 2815 749 in ZOE-pilot.

The third dataset (namely, the IBD data) is in a compositional form [7], and includes metatranscriptomes of fecal samples from 132 subjects for a 1-year period, with repeated measurements of the same participants over time. For our work, we only examined baseline (i.e. first visit) data, including a sample of 104 participants (52 male and 52 female). The dichotomized disease status of IBD (i.e. 50 Crohn’s disease and 26 ulcerative colitis cases) versus non-IBD (i.e. 28 ‘control’ participants) and the clinic location as a binary batch variable are included.

For all three datasets raw metagenomic (DNA) and metatranscriptomic (RNA) sequencing reads were submitted to Kraken2 [41] for taxa classification and host removal. Kraken2 estimates of taxa for each read were compiled into a single Bayesian estimate to sample taxa profile with Bracken [42]. Metagenomic reads identified as not being derived from host organisms were further processed by HUMAnN2 [43] to produce a second estimate of taxonomic profile (via MetaPhlAn2 [44]) and estimates of gene family abundance, path abundance and path coverage. Taking into account the abundance estimates derived from the metagenomic data, metatranscriptomic reads identified as not being derived from host were submitted to HUMAnN2 together with the metagenonmic estimates to produce estimates of gene family expression, path expression and path coverage. Gene family expression and abundance were estimated at the resolution of UniRef90 [45] clusters.

### Simulations

#### Simulation I: parametric simulations

In Simulation I, multiple scenarios are defined by the following generative models with a nested factorial design. Three data generative model classes were used: zero-inflated log-normal (ZILN), zero-inflated negative binomial (ZINB) and zero-inflated gamma (ZIG) models. On top of each generative model, three factors are further considered: (1) baseline distribution, (2) disease effects and (3) batch effects (without interaction with disease), each of which is further described in later sections. The parameters representing the three factors are selected after consideration of the estimated parameters of the gene expression in ZOE2.0, as well as the other datasets.

**Sets of parameters were chosen** for this simulation study so that they suitably represent distributions of parameter estimates in the example data. Once the data distribution is defined, we generate random samples to mimic a small ($n = 80$) study and a large ($n = 400$) study, where all four subgroups of disease-batch combinations are of equal size and there are $n_{\text{gene}} = 10\,000$ genes. Then we apply all tests listed in Section 2.1, and derive type I error, false discovery rate (FDR), sensitivity and accuracy. For most methods, type I error (sensitivity) is defined as how often the *P*-values are less than the 5% significance level, or ‘the rejection rate at 5%’, in the health (disease) group, and the Benjamini–Hochberg procedure-based adjusted *P*-values, or ‘$q$-values,’ are used to derive the FDR instead of type I error. Sensitivity is often called ‘power’ or ‘true positive rate’. Accuracy is given by the group-size weighted average of $1$—type I error and sensitivity. For LEfSe, however, because *P*-values are not available, we do not obtain its FDR; however, its type I error, sensitivity and accuracy are derived using the set of the genes declared significant instead of using the 5% cutoff. Because type I error, sensitivity and the proportion of the signal genes are sufficient to derive accuracy, and with a large proportion, it is mostly driven by type I error. In the following of Simulation I, we will mainly describe ZILN at the gene level based on ZOE2.0 and additional information is summarized at the end.

**Generative models** Three generative models are considered: zero-inflated log-normal (ZILN), zero-inflated gamma (ZIG) and zero-inflated negative binomial (ZINB). We do not include the zero-inflated beta (ZIB) distribution, as only a few methods, such as the LB test, model relative gene expressions or abundances rather than their absolute quantities. Furthermore, because ZIB can be considered a compositional transformation of independent ZIG distribution, ZIG-based results should serve as a good proxy for ZIB-based simulations. ZILN is a mixture of log-normal distribution and zeros, where the proportion of zeros is parametrized by $\pi $ and the nonzero values, after the log transformation, follow normal distribution with mean $\mu $ and variance $\sigma ^{2}$. Often in the literature, the model is parametrized using the overdispersion parameter, $\theta $, instead of the variance so that $\text{var}[Y|Y>0] = \mu ^{2}\theta $. ZILN is frequently used in practice both as a data generating model [23] and as a testing model (e.g. MAST and MGS). See ZINB and ZIG models in Section S4.

**Figure 1:**
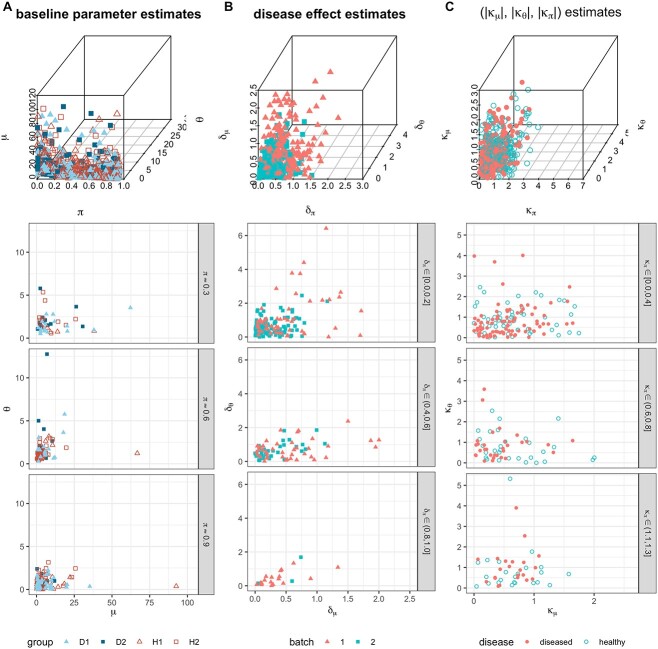
Column A: Parameter estimates of baseline ZILN distributions obtained from 300 randomly selected genes in ZOE2.0 with the three-dimensional scatter plot on the top row and each of the subsequent rows representing $\pi $ estimates being within 0.03 from 0.9, 0.6 and 0.3. Column B: Disease effect estimates based on ZILN models obtained from the ZOE2.0 data in absolute values $(|\delta _{\mu }|, |\delta _{\theta }|, |\delta _{\pi }|)$ Column C: Batch effect estimates based on ZILN models obtained from the ZOE2.0 data in absolute values $(|\kappa _{\mu }|, |\kappa _{\theta }|, |\kappa _{\pi }|)$.

**Baseline parameters** The ZILN parameters were estimated for the genes in the ZOE2.0 dataset for each of the disease and batch subgroups, using the method of moments (Figure 1A). The first, second and third quartiles of the $\mu $ estimates are 7.4, 19.7 and 52.6, respectively. Those for the $\theta $ estimates are 0.7, 1.2 and 1.8. Those for the $\pi $ estimates are 0.34, 0.64 and 0.83. Based on the parameter distribution, we selected sets of baseline parameters for the ZILN model (Table S2), which is a set of combinations of $\mu \in \{1, 5, 10\}, \theta \in \{0.5, 2.0\}, \pi \in \{0.3, 0.6, 0.65, 0.7, 0.75, 0.8, 0.85, 0.9, 0.95\} $.

**Disease effects** The disease effects are added in 10% of the genes with random perturbation of the direction—i.e. 5% of the genes have higher (lower) expressions for the disease group than for the healthy group, and 90% of the genes do not have any disease effects. Let $\mathbf \delta \equiv (\delta _{\mu }, \delta _{\theta }, \delta _{\pi })$ denote the disease effects such that $\log \mu _{D} = \log \mu + \frac 1 2 \delta _{\mu }$, $\log \theta _{D} = \log \theta + \frac 1 2 \delta _{\theta }$ and $\log \frac{\pi _{D}}{1-\pi _{D}} = \log \frac{\pi }{1-\pi } + \frac 1 2 \delta _{\pi }$, where $\mathbf \xi _{D} \equiv (\mu _{D}, \theta _{D}, \pi _{D})$ is the parameter for the diseased group. We simply denote such operation as $\mathbf \xi _{D} = g(\mathbf \xi , \delta )$. The parameter for the healthy group is $\mathbf \xi _{H} \equiv (\mu _{H}, \theta _{H}, \pi _{H}) = g(\mathbf \xi , -\mathbf \delta )$.

The disease effect estimates for ZILN are estimated from the genes from the ZOE2.0 dataset assuming that there are no batch effects (Figure 1B). The quartiles of the $\delta _{\mu }$ estimates are 0.1, 0.2 and 0.5 in the order. Those for the $\delta _{\theta }$ estimates are 0.3, 0.5 and 0.9. Those for the $\delta _{\pi }$ estimates with finite values are 0.2, 0.3 and 0.6. Based on the parameter estimates, we select sets of disease effects for ZILN model as in Table 1 LEFT. Note that the $\pi $ effect of scenario D4 is $-1$ to maintain consistency of the direction of the effects.

**Table 1:** Disease effects (LEFT) and batch effects (RIGHT). Additive effect size on log- (for $\mu $ and $\theta $) or logit- (for $\pi $) transformed scale; e.g. under $\mu \&\pi $ effect I (D6) without batch effects, the disease (healthy) group has one unit higher (lower) nonzero mean on the log scale and one unit lower (higher) zero proportion on the logit scale than the baseline. Under a large effect I (K3), within a disease group, a batch group has $2\times 1$ higher nonzero mean and dispersion on the log scale and $2\times 1$ unit lower zero proportion on the logit scale compared with the other batch group.

No.	$\mathbf \delta _{\mu }$	$\mathbf \delta _{\theta }$	$\mathbf \delta _{\pi }$	name	No.	$\mathbf \kappa _{\mu }$	$\mathbf \kappa _{\theta }$	$\mathbf \kappa _{\pi }$	name
D1	0	0	0	null effect	K1	0	0	0	null effect
D2	1	0	0	$\mu $ effect	K2	0.5	0.5	−0.5	small effect I
D3	0	1	0	$\theta $ effect	K3	1	1	−1	large effect I
D4	0	0	−1	$\pi $ effect	K4	0.5	−0.5	−0.5	small effect II
D5	1	1	0	$\mu \&\theta $ effect I	K5	1	−1	−1	large effect II
D6	1	0	−1	$\mu \&\pi $ effect I					
D7	0	1	−1	$\theta \&\pi $ effect I					
D8	1	0	1	$\mu \&\pi $ effect II					
D9	1	−1	0	$\mu \&\theta $ effect II					
D10	0	−1	−1	$\theta \&\pi $ effect II					

**Batch effects** The batch effects for the ZILN model are estimated for the genes in the ZOE2.0 and a random sample of these are presented in Figure 1 C. We considered binary batch effects as below. Let $\mathbf \kappa \equiv (\kappa _{\mu }, \kappa _{\theta }, \kappa _{\pi })$ denote the batch effects such that $\mathbf \xi _{d,1} = g(\mathbf \xi _{d}, \mathbf \kappa )$ and $\mathbf \xi _{d,2} = g(\mathbf \xi _{d}, -\mathbf \kappa )$ are the distribution parameters for disease group $d=D, H$ in batches 1 and 2, respectively. Alternatively, we denote $\mathbf \xi _{D,1} = g(\mathbf \xi , \mathbf \delta , \mathbf \kappa )$. The quartiles of the $\kappa _{\mu }$ estimates are 0.3, 0.6 and 1.0 in the order. Those for the $\kappa _{\theta }$ estimates are 0.3, 0.7 and 1.2. Those for the $\kappa _{\pi }$ estimates with finite values are 0.4, 0.8 and 1.4. Based on the ZILN parameter estimates, we select sets of batch effect parameters for the ZILN model as in Table 1 RIGHT.

**Other simulation scenarios** Summaries of the estimated parameters for simulations in other generative models (ZINB, ZIG), those for the validation datasets (ZOE-pilot, IBD) and those for the additional levels of measurement (gene-species, species marginal) are presented in Section S5. The estimated ZILN parameters of genes’ expression distribution in ZOE-pilot (Figure S5A) and the IBD data (Figure S6A) have ranges that overlap reasonably with Figure 1A and the sets of parameters (Table S2 and Table 1). Based on ZOE2.0, the distributions from the gene-species joint data and the species marginal data (Figure S5.6) are reasonably covered by the parameter sets chosen at gene level for both ZILN (Table 1) and ZINB, except the ZINB model for the marginal species data where the parameters are often either not estimable or outlying.

#### Simulation II: semi-parametric simulations

For each of the three datasets—ZOE2.0, ZOE-pilot and IBD—we randomly sample a large number of genes (1000 or 10 000) among the prevalence-filtered ($\ge $10%) genes in real data. We have two types of semi-parametric setups. One is to add synthetic disease effects to highlight the sensitivity of the tests and the other is to shuffle the disease labels among samples to evaluate the type I error. In the first setup, we added signals of synthetic disease effects to the 10% of genes, termed as ‘signal genes’, in the dataset for evaluation of FDR, sensitivity and accuracy. For each dataset, $n_{\text{genes}}$ = 1000, 10 000 genes were randomly selected among which $n_{\text{signal}}$ = 100, 1000 signal genes were further selected for differential $(\mu ,\pi )$ disease effects. Note that we did not consider the $\theta $ effects, as they are not of interest in general, and $\mu $ and $\pi $ are the parameters that quantify the overall mean expression levels. To this end, we utilize the set of disease effects estimates obtained in Section 2.3.1 and choose only the subset tuples $(\delta _{\mu }, \delta _{\pi })$ such that $|\delta _{\mu }|\ge 2$. From this large effect size tuples, $n_{\text{signal}}$ tuples were randomly selected and were assigned to each of the signal genes. These effect size tuples were then applied to the disease groups following a rule described in Section S3. Subsequently, each of the 10 analysis methods was applied to each of these semi-parametric simulated datasets. This was repeated 10 times. Method performance was evaluated using the same metrics as in Simulation I over the 10 replicates.

The second setup of the semi-parametric simulation is an alternative way of evaluating the methods in terms of Type I error. We generate the data under the global null hypothesis via permutation of the disease labels of samples. For each method, we calculated the number of rejections out of 10 000 genes averaged over 100 permutation replicates.

## RESULTS

### Difference between the metagenomics and metatranscriptomics data

There were high proportions of zero gene expressions in both the ZOE2.0 (80.4%) and ZOE-pilot (87.9%). These high zero proportions are comparable and actually higher than what is encountered in the corresponding metagenomics data (75% in ZOE2.0 and 68% in ZOE-pilot) (Figure S1). Specifically, 89% (83%) of genes have > = 80% zero proportions in metagenomics compared with 94% (97%) in the metatranscriptomics data of the ZOE2.0 (and ZOE-pilot) data. 54% (43%) of genes have $\ge 90\%$ zero proportions in metagenomics compared with 59% (71%) in the metatranscriptomics data of the ZOE2.0 (and ZOE-pilot) data. Similarly as in ZOE studies, IBD study has the consistent trend of higher zero proportions in metatranscriptomics over metagenomics data (Figure S1), as the average proportion of zeros per gene in the IBD metatranscriptomics data is 96.3%, while that in the metagenomics data is 87.8%, summarized in Web Table S1.

Overdispersion, at the gene level and at the species level, was also examined when exploring the differences between metagenomics and metatranscriptomics data in the three datasets (Figure S2 and Figure S3) under the ZILN model. In our example datasets, when proportion of zeros in metatranscriptomics data is slightly, not dramatically, higher than in the metagenomics data (such as for the species level in ZOE 2.0, the gene level in ZOE 2.0 and the species level in IBD), overdispersion is higher in metatranscriptomics than in metagenomics data. When the percentage of zeros is dramatically higher in metatranscriptomics data (at least 9 to 10% higher, as seen in Web Table S1) compared with in metagenomics data (such as for the species level in ZOE-pilot, the gene level in ZOE-pilot and the gene level in IBD), overdispersion is lower in metatranscriptomics than in metagenomics data.

The effects of the above difference of choice of methods are reflected in the comprehensive simulation scenarios, which allow for different values of proportion of zeros $\pi $ and overdispersion $\theta $ that covers the range and variation of these two parameters.

### Goodness of fit of the generative models for metatranscriptomics data

**Figure 2:**
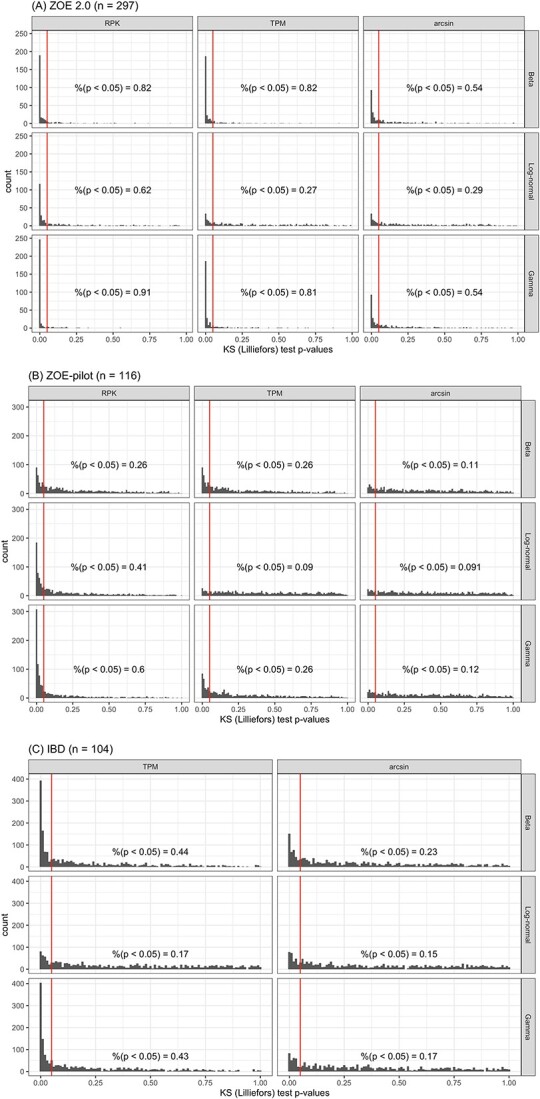
Goodness of fit (Kolmogorov–Smirnov, KS) test results for Beta, Log-normal and Gamma distributions (rows) with different scaling/transformation methods (columns). Histograms of the number *P*-values of the KS test, based on randomly select 300 genes in each of the three datasets (A) ZOE2.0, (B) ZOE-pilot, (C)IBD. The IBD data are available only in a compositional form, and thus we do not consider RPK in IBD. Lower rejection rate suggests better model fitting.

We evaluate the goodness of fit of the three statically generative models (ZILN, ZIG and ZINB) in the three real datasets with consideration of three transformation methods, to ensure a proper distribution was used for the parametric simulation. As another reference distribution and the one that the discrete part of the LB test is based on, Beta distribution is also included. For the continuous values (in ZILN, ZIG and ZIB), we applied the Lilliefors procedure [46], a data-adaptive version of the Kolmogorov–Smirnov (KS) test [47] to evaluate the similarities. The log-normal model shows a consistently good fit in all datasets (Figure 2). The rejection rate in ZOE2.0 (Figure 2A) is overall higher than in ZOE-pilot (Figure 2B), due to the redbetter power in larger sample sizes. TPM normalization provides a better fit compared with RPKs (Figure 2A–B). For the log-normal distribution, almost identical results between TPM and arcsine transformations are observed (Figure 2). This is because, with a large number of genes in the data ($n_{\text{genes}}> 300\,000$ for ZOE2.0), most compositional ($TPM/c$) values are close to zero and, consequently, the arcsine transformation is essentially equivalent to the square root transformation with scaling: 


\begin{align*} &arcsin(\sqrt{x}) = \sqrt{x} + O(x^{\frac32}), \ \text{ as}\ x \to 0^+.\end{align*}

This implies that the arcsine transformation is merely a location-shift transformation in the log-normal model for most of compositional values. LB showed slightly worse fit than ZILN. Meanwhile, the ZINB distribution has overall a reasonable fit to the RPK or the TPM transformed data, but has a poor fit to the arcsine transformed data (Section S6.1). Overall, ZILN and ZINB with TPM have better fit of data and will be used for parametric simulation of data. We still keep ZIG as a reference distribution in parametric simulation.

### Simulation I (parametric simulations) results

#### Type I error and FDR

**Figure 3:**
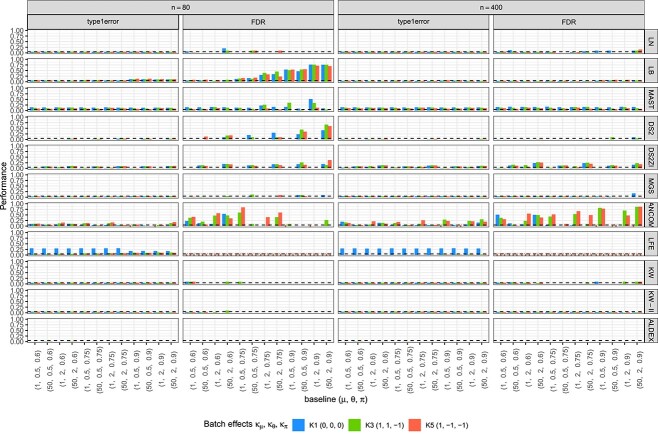
Type I error rates (under the Null D1) and FDR (Under the Alternative of mean, D2) for ZILN models. Columns correspond to sample sizes and evaluation criteria, rows are different tests, the $X-$axis represents baseline distributions and colors indicate batch effects. The dotted horizontal lines denote the significance level (5%). A failure in evaluation is marked as $\times $ to be discerned from zero. DS2 = DESeq2, DS2ZI = DESeq2-ZINBWaVE, ANCOM = ANCOM-BC2, LFE = LEfSe, ALDEX = ALDEx2.Because p-values of LEfSe are not available, we do not obtain its FDR, also seen in Methods for Simulation I.

We compared the performance of the 10 methods using the ZILN-based simulated data for the null hypothesis of mean shift (D1 in Table 1) or the $\mu $ effects ($\mu _{D} = \mu _{H})$ for both sample sizes in Figure 3. Overall, type I error and FDR are well or reasonably controlled in LN, MGS, KW, KW-II and ALDEx2. Contrary, LEfSe with its default test thresholds—0.05 for Kruskal–Wallis and 2 for LDA—has higher than 5% type I error frequently, implying that LEfSe requires tuning (i.e. lowering) of the thresholds to avoid high type I error. MAST has type I error and FDR that are often higher than the nominal significance level even for a larger sample size. ANCOM-BC2 also frequently has inflated type I error and FDR, which is more evident under the batch-effects scenarios possibly due to model mis-specification. Type I error and FDR in ANCOM-BC2 are often amplified with a larger sample size, indicating that the error is not due to a finite sample size but could be a systematic bias. For the other two-part models (LB and DESeq2-ZINBWaVE), type I error is less stably controlled, especially when the zero-inflation (or zero proportion) parameter is high as a finite sample bias. For example, for a ZILN sample of size $n=80$, $\pi =0.9$ means that there are only eight nonzero values on average and that large sample theory may not be applicable. Thus, when the sample size is not large, and proportion of zeros is high, two-part models are not recommended without knowledge that the posited distribution of the test agrees with the true underlying distribution. As expected, DESeq2, originally designed for negative binomial distributions without zero-inflation, has a very low type I error when used to model these zero-inflated data. On the other hand, DESeq2-ZINBWaVE has on average higher type I error than DESeq2. However, it has higher-than-nominal type I errors for larger baseline nonzero mean values, and the inflation becomes even larger for a large sample, implying that the aberration may not be attributable to the finite sample bias. Designed for the scRNAseq and with its unstable control of type I error, the ZINB-WAVE extension of DESeq2 should be used with caution. Type I error and FDR results under ZINB and ZIG models are presented in Figures S14 and S17.

To connect with the difference between metagenomics and metatranscriptomics data, we find that modifying overdispersion has larger effects on FDR of ANCOM-BC2 and LB in simulations. ANCOM-BC2 with a large sample size has high FDR when overdispersion $\theta $ is 2, while the FDR is very small when $\theta $ is 0.5. LB with a sample size has overall larger FDR than other methods when the proportion of zeros increases, and this inflated FDR is enhanced by high overdispersion.

#### Sensitivity in a small sample ($n=80$)

**Figure 4:**
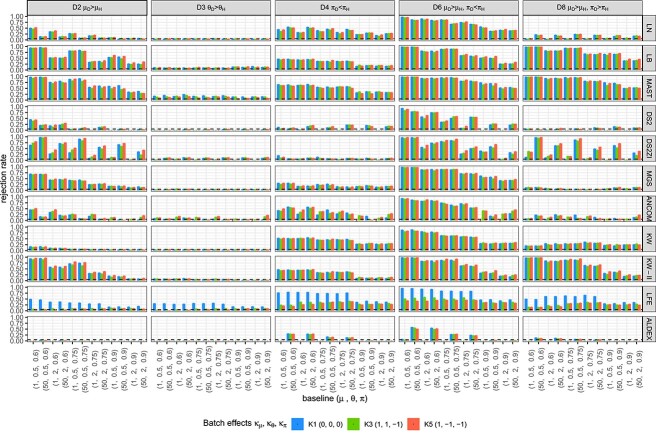
Sensitivity under alternative ZILN distributions for a small sample ($n = 80$). Columns and rows correspond to tests and alternative distributions, respectively, the $X-$axis represents baseline distributions and colors represent batch effects. The dotted horizontal lines denote the significance level (5%). A failure in evaluation is marked as $\times $ to be discerned from zero. DS2 = DESeq2, DS2ZI = DESeq2-ZINBWaVE, ANCOM = ANCOM-BC2, LFE = LEfSe, ALDEX = ALDEx2.

The rejection rates at 5% cutoff for the signal genes under alternative distributions, or the sensitivity of the tests, are illustrated in Figure 4 for the ZILN model and sample size of $n = 80$. To connect with the difference between metagenomics and metatranscriptomics data when the sample size is small, we find that modifying overdispersion has effects on the power of almost all of the methods except LN in D2, which is the most importance scenario for detecting mean shift. This mostly holds true for D6, which has one component similar to D2. Methods that do not have this pattern, such as LEfSe and ALDEx2, hardly have good power in D2.

**D2 ($\mu _{D}>\mu _{H}$)**. When the disease status is only associated with the difference in nonzero means ($\mu $), many methods have high sensitivity for most of the baseline scenarios—LB, MAST, DESeq2-ZINBWaVE, MGS, LEfSe, KW-II—and methods such as LN and ANCOM-BC2 have moderate level of sensitivity over various settings. However, while the favorable sensitivity of MAST, DESeq2-ZINBWaVE, LEfSe and ANCOM-BC2 comes at a cost of inflated type I error (and FDR), the type I error of LB is relatively reasonably controlled for large sample sizes, and that of MGS is well controlled. KW-II, which often has one of the highest sensitivities, has relatively weak sensitivity in high zero-proportion scenarios. LN has good sensitivity for many baseline scenarios but lacks sensitivity when the zero-proportion is high ($\pi = 0.9$). This is due to the bias from model misspecification of the LN model. KW suffers from low sensitivity with even smaller $\pi $. DESeq2 has a reasonably good sensitivity when the zero inflation is not high ($\pi \le 0.6$). However, it often has lower than 5% sensitivity when the data are sparse. This again can be explained by DESeq2’s inability to model zero-inflation. Finally, ALDEx2 suffers from lack of power, sensitivity lower than 5%, which can be explained in part by the Dirichlet prior $\text{Dir}(\frac 12, \frac 12,..., \frac 12)$ unduly dominating the expression levels of the relatively sparse and low-expressed compositional data.

**D3 ($\theta _{D}> \theta _{H}$)**. Most tests lack power in detecting difference in $\theta $ (D3), which is expected as all the tests considered in this paper detect the marginal or conditional mean differences and $\theta $ difference alone does not affect the mean. However, there are quite a few methods that have sensitivity greater than 5%. Methods with inflated type I error, such as MAST, are expected to have rejection rates higher than 5%. The equal variance assumption that is implied in methods could also be a source of the inflation.

**D4 ($\pi _{D}> \pi _{H}$)**. Differential disease effects only in $\pi $ are captured by methods such as LB, LN, KW, LEfSe and MAST. MAST and LEfSe’s higher sensitivity compared with other methods is counterbalanced by inflated type I error rates. Under the setting D4, relatively low sensitivity for baseline $\pi = 0.9$ and high sensitivity for baseline $\pi = 0.6$ can be explained by the design of the experiments. When the baseline $\pi $ is close to 1 or 0, the absolute difference $(\pi _{D} - \pi _{H})$ between two groups is relatively smaller than that when the baseline $\pi $ is close to 0.5.

**D6 ($\mu _{D}> \mu _{H}, \pi _{D} < \pi _{H}$)**. Sensitivities are higher for D6 ($\mu _{D}> \mu _{H}, \pi _{D} < \pi _{H}$) than for both D2 ($\mu _{D}> \mu _{H}$) and D4 ($\pi _{D} < \pi _{H}$), as D6 is expected to have larger marginal mean differences than D2 and D4. As a result, most tests have sensitivities $\ge 0.50$ for $\pi \le 0.9$ including LN and KW.

**D8 ($\mu _{D}> \mu _{H}, \pi _{D} > \pi _{H}$)**. The disease effect scenario D8 is complicated, as the signal from $\mu $ difference and that from $\pi $ difference offset the effect on the marginal mean. Thus, tests based on marginal models, i.e. single-part models such as LN, KW, MGS, DESeq2 and ANCOM-BC2, inherently cannot avoid low sensitivity under this scenario, because they do not separate two opposite signals from two parts. Consequently, they have lower rejection rates under D8 than under either D2 or D4. In contrast, two-part models (LB, MAST, KW-II) entertain the two distinct signals resulting in almost the same sensitivity as in D6.

**Other scenarios involving $\theta $ (D5, D7, D9, D10)**. Other scenarios involving $\theta $ such as D5 ($\mu _{D}> \mu _{H}, \theta _{D} > \theta _{H}$), D7 ($\theta _{D}> \theta _{H}, \pi _{D} < \pi _{H}$), D9($\mu _{D}> \mu _{H}, \theta _{D} < \theta _{H}$) and D10($\theta _{D} < \theta _{H}, \pi _{D} < \pi _{H}$) do not result in differences compared with scenarios without $\theta $ effects, i.e. D2, D4, D2 and D4, respectively (Section S7.1). This is expected because $\theta $ differences do not affect the marginal mean difference and the methods considered in this paper treat $\theta $ as a nuisance parameter.

**Sensitivity in the presence of batch effects.** The presence of batch effects affects sensitivity even when the batch information is incorporated in the tests. This could be due to the fact that batch effects are made multiplicatively in the generative models, while the tested models only consider main disease and batch effects without interactions. Nevertheless, the unevenness of sensitivity across different batch-effect scenarios is neither dramatic nor systematic. The patterns, e.g. higher sensitivity for MAST and LB, lower sensitivity for large $\pi $ values and so forth, discussed in earlier sections, still hold across different batch-effect scenarios.

#### Sensitivity in a large sample ($n=400$) and in other scenarios

**Figure 5:**
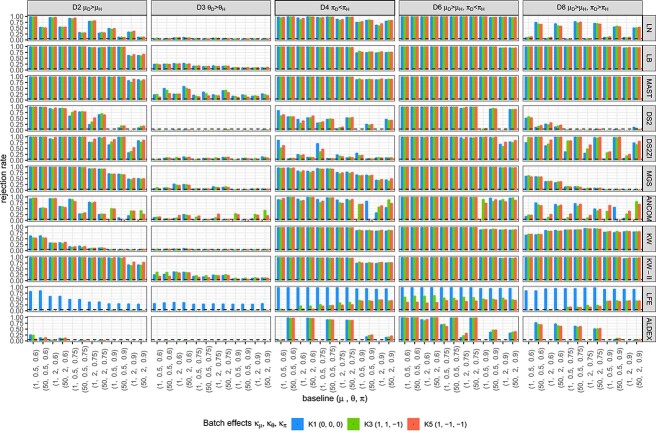
Sensitivity under alternative ZILN distributions for a large sample ($n = 400$). Columns and rows correspond to tests and alternative distributions, respectively, the $X-$axis represents baseline distributions and colors represent batch effects. The dotted horizontal lines denote the nominal significance level (5%). A failure in evaluation is marked as $\times $ to be discerned from zero. DS2 = DESeq2, DS2ZI = DESeq2-ZINBWaVE, ANCOM = ANCOM-BC2, LFE = LEfSe, ALDEX = ALDEx2.

Higher power is achieved in a larger sample size (Figure 5). The performance patterns are mostly the same for both sample sizes; MAST, LB and MGS have the highest sensitivity under most scenarios, two-part models have higher sensitivity than single-part models when signals are in the opposite directions as in D8 and $\theta $ difference (D3) is not properly detected for most of the tests.

The patterns of rejection rates under ZINB and ZIG models are not very different from those under ZILN models (See full results in Figures S12–S16).

**Figure 6:**
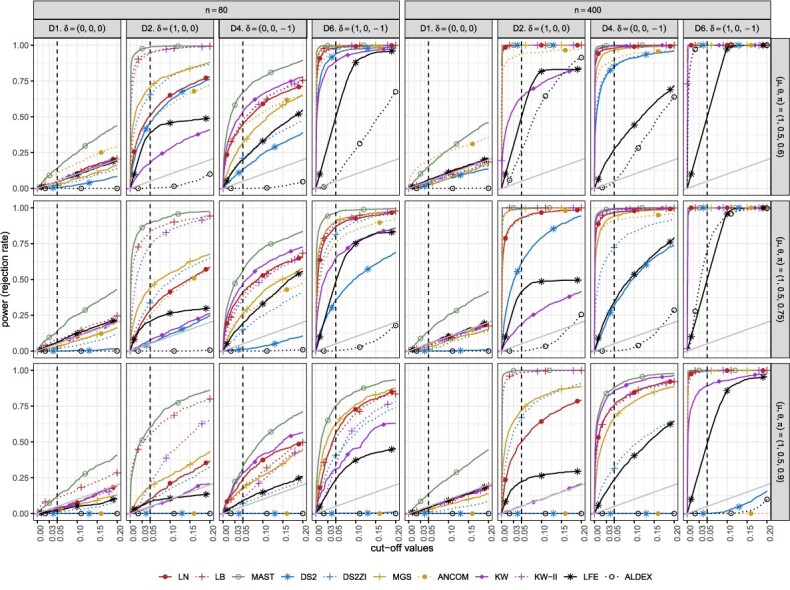
Sensitivity curves of DE tests according to different cutoff values ranging from 0 to 0.2 and a few baseline and disease-effects scenarios of the ZILN model. No batch effects are simulated in these scenarios. The gray solid diagonal lines denote the nominal significance level.

To provide a broader view of the sensitivity of the tests we vary the significance levels and the disease effect sizes, respectively. On one hand, the pattern of the previous results is mostly preserved with a different choice of cutoff values, as each curve either dominates or is dominated by the others for most of the settings uniformly over varying cutoff values (Figure 6).

**Figure 7:**
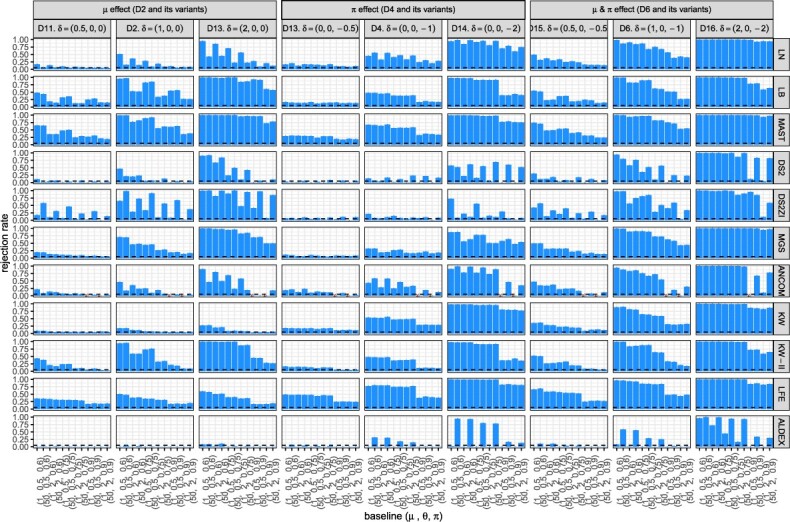
Sensitivity of the DE tests according to different effect sizes for a subset of the baseline scenarios and $n=80$. No batch effects are simulated. A failure in evaluation is marked as $\times $ to be discerned from zero. DS2 = DESeq2, DS2ZI = DESeq2-ZINBWaVE, ANCOM = ANCOM-BC2, LFE = LEfSe, ALDEX = ALDEx2.

The pattern of higher sensitivity for larger disease effect sizes was expected (Figure 7). However, it is noteworthy that when there are only small disease effects on the nonzero mean (i.e. Scenario D11), some methods have virtually zero sensitivity (DESeq2 and KW) or very low sensitivity (LN), suggesting that to compensate for the lack of fit, the effect size must be sufficiently large.

#### Computation time

The average computation time for testing 1000 genes with sample size 400 under D2 was 121, 194, 121, 482, 283 and 93 min for MAST, DESeq2, DESeq2-ZINBWaVE, ANCOM, LEfSe and ALDEX, respectively, less than 10 min for LB and MGS, and less than a minute for LN, KW and KW-II.

### Simulation II (semi-parametric simulations) results

**Figure 8:**
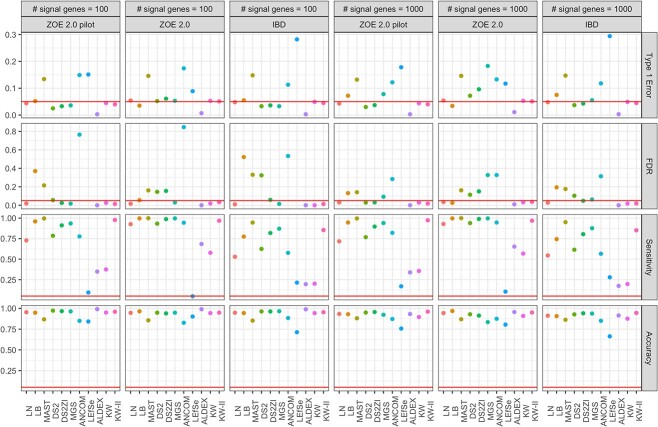
Performances of the analysis methods under semi-parametric simulations. 1000 (first three columns) or 10 000 genes (last three columns) were randomly selected for simulation. 10% of these genes, as specified in the panel heads, were given artificial disease effects. DS2 = DESeq2, DS2ZI = DESeq2-ZINBWaVE, ANCOM = ANCOM-BC2.

**Figure 9:**
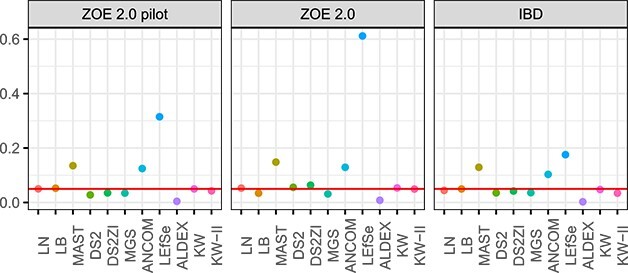
Type I error rates under the global Null condition generated by permuting the disease labels of samples in each of the three studies. 10 000 genes were tested. 100 permutations were generated. DS2 = DESeq2, DS2ZI = DESeq2-ZINBWaVE, ANCOM = ANCOM-BC2.

The semi-parametric simulation results mostly confirm the parametric data simulation results. In the first setup where a proportion of genes have signals (Figure 8), MAST, ANCOM-BC2 and LEfSe have high type I error and FDR, and those for LB are high for small sample sizes (ZOE-pilot and IBD) but are well controlled in the larger datasets (ZOE2.0). Type I error and FDR for the other methods are reasonably controlled, and especially those for ALDEx2 are lower than the threshold. Sensitivity is often high for most of the methods except KW, LEfSe and ALDEx2. When the sample size is relatively large (e.g. ZOE2.0) and the proportion of the signal genes is large, higher sensitivity is observed for most of the methods—LB, MAST, DESeq2, DESeq2-ZINBWaVE, MGS, ANCOM-BC2, LN. Among those, LB and LN have type I error and FDR less than or equal to the nominal level (5%), and DESeq2 and DESeq2-ZINBWaVE have type I error $\le 10\%$ and FDR $\le 20\%$. Interestingly, MGS, which had good control of type I error, has type I error greater than 10% in this specific setting. In the second setup under the global Null generated by sample permutation, LEfSe, MAST and ANCOM hardly controls the type I error (Figure 9), also consistent with the parametric simulation and above semi-parametric simulation results.

### Application in DE analysis

We applied the highest performing statistical analysis methods—LN and LB—to the sizeable metatrascriptomics datasets generated in the two aforementioned microbiome studies, ZOE2.0 (oral) and IBD (gut), to identify microbial genes whose expression is significantly associated with ECC or IBD, respectively.

#### ZOE2.0

**Figure 10:**
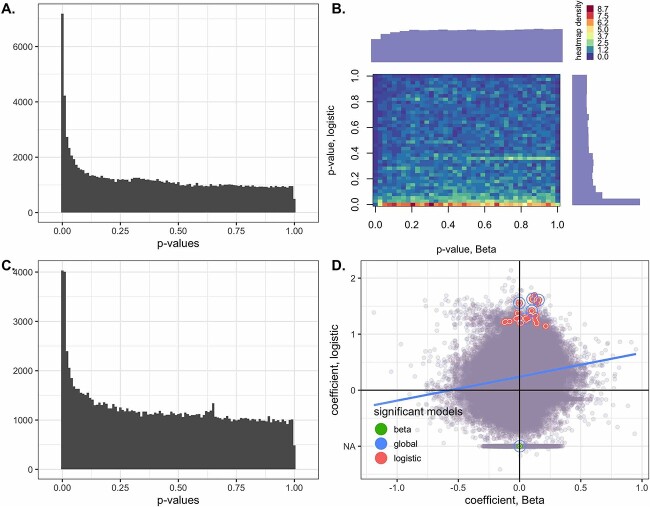
Application to the ZOE2.0 data analysis results. A. Histogram of the *P*-values of the log-normal models. B. Histogram of the joint *P*-values of the logistic Beta models (x-axis is for the Beta part and y-axis is for the logistic part). C. Histogram of the global *P*-values of the logistic Beta models (Wald test statistics). D. Scatter plot of the coefficients (disease effects on nonzero proportions and nonzero means) of the LB models, with the circled dots representing the most significant genes—Wald test statistic $P < 10^{-5}$ for the three types of Wald tests. Blue is for the global test, red is for the logistic part and green is for the Beta part. NA on the y-axis indicates that the logistic part was not estimated.

In ZOE2.0, out of 402 937 microbial genes, after filtering out low-expressed genes with prevalence rate $<0.1$ or average TPM $<0.2$, 157 113 microbial genes were tested for comparison between ECC and non-ECC, including age (in months) and batch effects as covariates. For the LN test, the minimum positive value (0.007) was uniformly added to the TPM values. Nominal *P*-values are reported for illustration purposes (Figure 10). For each gene in LB, two Wald test *P*-values of the disease effect for the two parts, as well as the global *P*-value, are reported. Genes differentially expressed (among a total of 157 113 genes) between ECC and non-ECC participants were evidenced by the high peak on the left side of the histogram of nominal *P*-values emanating from both LN and LB (Figure 10A,C). We also found that (1) no genes were significantly differentially expressed in both parts of LB and that (2) significant genes based on the global *P*-value of LB have same-sign coefficients in both parts (Figure 10D). Whether these two observations are typical in metatranscriptomics DE analyses needs to be further investigated in future studies.

Most of the significant DE genes in the LB models are also reported as significant in the LN models (Table S12). Similar results are obtained for the gene-species level of analysis (Section S8). Biological annotations and interpretations of these genes and species are available using UniRef90 [45](Section S8.4). In both the LN and LB tests, we found that genes C8PHV7 and C8PEV7, harbored by the lactate-producing *Campylobacter gracilis*, had the strongest association with childhood dental disease and may contribute to the development and establishment of ECC [48].

#### IBD study

In the IBD study, out of 1119 472 genes, 103 966 genes with prevalence rate $>0.1$ and mean expression level $>10^{-8}$ in the relative RPKs were tested between IBD and non-IBD, using gender and batch effect as covariates. For the LN test, the minimum positive value ($5\times 10^{-10}$) was uniformly added to the TPM values. Nominal *P*-values are reported. Similarly to the ZOE data analysis results, the existence of DE genes is evident (i.e. high peak on the left in Figure 12A and C, and Figure 13). Only the continuous part has a conspicuous hike, while the discrete part is mostly flat (12B). This indicates that the association signal is dominated by the continuous part, which is also confirmed in the scatter plot (Figure 12D). It is noteworthy that about 4% of genes have very high discrete model coefficients but are insignificant in two clusters around either $\pm 26$ on the y-axis.

**Figure 11:**
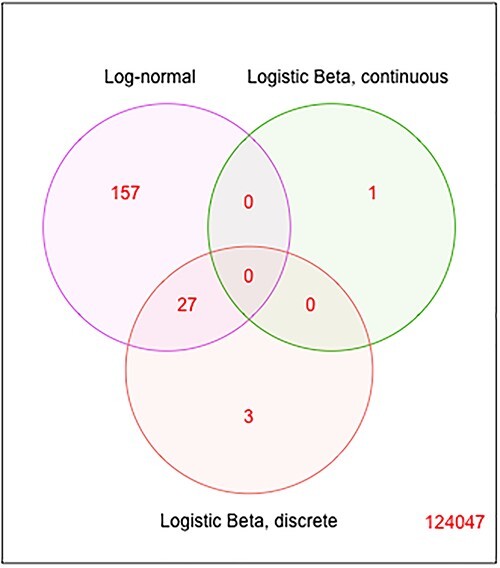
Venn diagram of DE genes in LN and the two parts of LB, at a *P*-value cutoff of $10^{-5}$ in ZOE2.0 metatranscriptome data.

**Figure 12:**
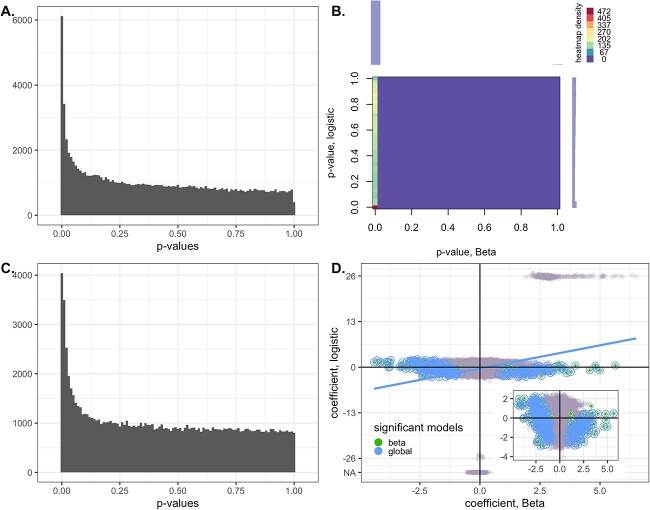
Application to the IBD data analysis results. A. Histogram of the *P*-values of the log-normal models. B. Histogram of the joint *P*-values of the logistic Beta models (logistic and Beta parts). C. Histogram of the global *P*-values of the logistic Beta models (Wald test statistics). D. Scatter plot of the coefficients of the LB models, with the circled dots representing the most significant genes—Wald test statistic $P < 10^{-5}$. The NA results around $y = \pm 26$ indicate that the logistic part was not estimable.

**Figure 13:**
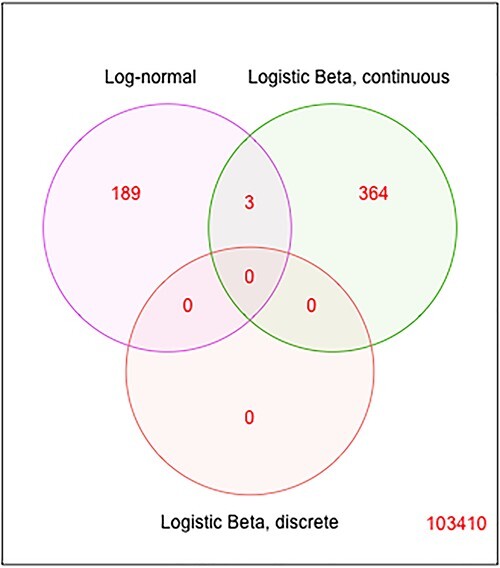
Venn diagram of genes with *P*-values less than $10^{-5}$ for each evaluated model in the IBD data.

With regards to the microbial genes significantly associated with IBD in the LN model, we present their corresponding proteins, functions and species in Table S13 in Section S9. Most of the top 20 genes in the LN tests correspond to *Sutterella wadswothensis* [7]. However, the most significant genes in LN are very different to the most significant genes identified using the LB models (Figure 13). Biological annotations and interpretations of these genes and species are available in (Section S9.3).

## DISCUSSION

We have provided a comprehensive evaluation of the 10 main analysis methods for differential gene expression of metatranscriptomics data as summarized in Table 2. The simulation study design was inspired by actual human oral and gut microbiome sequencing data, to which we investigated the goodness of fit of the three generative models after transformations, to select the proper distributions for simulation. The 10 DE methods were evaluated in terms of control of type I error, sensitivity and FDR under the simulation of three parametric models, ZILN, ZINB and ZIG. The semi-parametric simulations using both the oral and gut microbiome sequencing data were further performed as a complement to the parametric simulations. The simulation study offers guidance to microbiome investigators for choosing appropriate DE analysis methods. It suggests that the log-normal distribution fits reasonably well to the oral microbiome data after the TPM transformation. In both the parametric simulations and the semi-parametric simulations, the LB tests showed the highest sensitivity while controlling for the type I error in a large sample. In addition, MGS has comparably high sensitivity with a well-controlled type I error but does not allow batch effects. MAST and ANCOM-BC often suffer from inflated type I error even with large sample size. In reality, DE only in nonzero mean (D2), only in zero proportion (D4) or in both nonzero mean and zero proportion with the opposite direction (D6) is of most interest and is more feasibly observed than the others. For details, the LB and MGS tests that have high sensitivity under D2 and D6 and the LN test that has high sensitivity under D4 and D6 are noteworthy. KW and KW-II tests have high sensitivity under D6. However, KW has very low sensitivity for most of the settings of D2 and KW-II suffers from low sensitivity when $\pi =0.9$ under all of D2, D4 and D6. MGS does not have good sensitivity under D4 ($\pi $-differences) and LB needs to be used with caution as it may have an inflated type I error for a high zero proportion with small sample size.

**Table 2:** Summary of performances. DESeq2ZI is the abbreviation of DESeq2-ZINBWaVE. Sensitivity (I) is sensitivity in parametric simulations; Sensitivity (II) is sensitivity in semi-parametric simulations. $\bigcirc $ models zero counts and considers the differential effects on zeros in the tests. $\triangle $ models zero counts but the tests only the marginal or nonzero mean differences. $\times $ does not model zeros.

method	type I error & FDR	sensitivity (I)	sensitivity (II)	zero model	input data	computation	other
LB	fair	high (D2, D4, D6, D8)	high	$\bigcirc $	TPM or proportion	moderate	Inflated type I error for high $\pi $ and small $n$ in D2.
MGS	good	high (D2, D6)	high	$\triangle $	TPM	moderate	No batch control available.
LN	good	good (D4, D6)	mid-high	$\times $	TPM	light	Low-powered for high $\pi $ in D2.
MAST	not controlled	high	high	$\bigcirc $	TPM	heavy	type I error not controlled.
ANCOM-BC	not controlled	good (D4, D6)	mid-high	$\triangle $	TPM	heavy	type I error not controlled.
DESeq2	good	low	mid-high	$\times $	counts	heavy	
DESeq2ZI	unstable	good	high	$\triangle $	counts	heavy	type I error not controlled
LEfSe	not controlled	good	low	$\times $	TPM	heavy	$p-$ value is not provided.
ALDEx2	good	good (high $\mu $)	low	$\times $	TPM	heavy	Fine tuning of the scale ($\mu $) is required.
KW	good	good (D4, D6)	low	$\times $	TPM	light	
KW2	good	good (low $\pi $)	high	$\bigcirc $	TPM	light	Low-powered when $\pi \ge 90\%$.

Based on the figures from parametric simulation where the simulated data were generated in ZILN distribution (Figure 3, 4, 5 for type I error, FDR, sensitivity), we provide the following three recommendations when choosing DE methods. First, when sample size is relatively small (n=80) and zero proportion is high, this is a very difficult condition for tests. MAST performs well though it has an overall slightly inflated type I error. ANCOM-BC sometime works fine but not very stable. Second, when sample size is relatively small (n=80) and zero proportion is not high, LB and KW-II perform well. MAST and MGS both are decent but MAST has slightly inflated type I error, while MGS does not perform well when one sample group has higher nonzero average but also higher zero proportion, which is possible in real data. Last, when sample size is relatively large (n=300) like in many large cohort studies, zero proportion has less effects for choosing the better DE testing methods. Overall, MAST, LB, KW-II, MSG and DESeq2-ZINBWaVE all perform acceptable. However, LB is the preferred one, followed by KW-II and MAST. These three methods perform similarly, except that KW-II only allows one covariate (batch) and MAST still has slightly inflated type I error in all simulations. MGS is still a good option if this scenario is not a concern ‘when one sample group has higher nonzero average but also higher zero proportion’, same as LN. In addition, Figure 6 and 7 under the same simulations, as well as Figures 8 and 9 from the semi-parametric simulation (sample-permutation based in real data), provide further confirmation of the above conclusions. Supplementary figures 10–15 under the distribution of ZINB and ZIG have similar conclusions but with some variation. For other methods, DESeq2 without ZINBWave has both low type I error and sensitivity for metatranscriptomics data, and the one with ZINBWave shows unstable control of type I error. LEfSe, which does not provide *P*-value, needs to be used with carefully selected thresholds to avoid inflated type-I error. The best-performing methods were further used for detecting the DE genes in the childhood dental disease metatranscriptomics data and the IBD gut metatranscriptomics data.

In the oral microbiome application, we found the relationship between the two parts in the LB test may strengthen or weaken the justification for the global Wald statistic. In rare events where the signals from the two parts are both strong and with opposite signs, the Wald statistic can detect the signals that would have vanished if the two effects were marginalized. On the other hand, when the signal from only one part is strong, while the other is not, the Wald statistic may not be able to detect the strong signal after being diluted by the weak one, resulting in low sensitivity. In this case, using the minimum *P*-values from both parts with an adjusted significance level, i.e. twice the nominal values for the Bonferroni-type adjustment, could be an alternative strategy.

In the application to IBD, we observed the unexpected manifestation of the undesirable feature (Figure 12D) of Wald statistics called the Hauck–Donner effects, where larger disease effects may not always result in a larger statistic and, as a consequence, may yield lower sensitivity [49]. This occurs when a phenotype group has a prevalence rate of exactly zero or one, while the other group has a prevalence rate away from zero and one. The likelihood-ratio test, permutation tests, Fisher’s exact test, regularization and Bayesian approaches are the alternatives to the Wald test. Among the 523 candidate genes with such prevalence rate pattern, no genes were found significant at *P*-value $10^{-5}$ nominal statistical significance threshold by the likelihood-ratio test and the Fisher’s exact test of which *P*-values are presented in Figure S24.

**Key Points**

- The choice of optimal gene-level DE analysis methods in metatranscriptomics data is a key question, to best account for the high percentage of zeros, overdispersion and possibly compositional data structures. Sample size, the existence of covariates or batch-effects variables and the data distribution are all characteristics affecting the choice of optimal analysis methods.
- We systematically evaluated 10 methods with a focus on statistical models that were specifically designed for zero-inflated overdispersed counts or compositional data and particularly for DE analysis at the most sparse and challenging microbial gene (versus species) level.
- LB and MGS have the highest sensitivity among others, with inflated type-1 error for sparse, small-sized data in LB and without the capacity of batch effects control for MGS. LN has reasonably good sensitivity and type I error control under various settings. Although it is the most comprehensive evaluation of DE analysis for the metatranscriptomics gene level, there are reasonable limitations of recommending DE methods due to the number of available datasets.
- Based on the LN and LB tests, genes C8PHV7 and C8PEV7, harbored by the lactate-producing *Campylobacter gracilis*, have the strongest association with childhood dental disease.
- We have focused primarily on statistical significance largely because statistical significance is more suitable to quantitative analysis, like DE analysis. The most significant microbial DE genes are expected to have enriched with functional activities. We have explored the biological interpretation of the DE genes in the real data application. However, we acknowledge interpretation of biological importance of the DE genes has a limitation in this paper.

## Supplementary Material

Microbiome_simulation_Appendix_bbad279

## Data Availability

The reference datasets analyzed in this manuscript are publicly available. ZOE2.0 data are available in the dbGaP repository https://www.ncbi.nlm.nih.gov/gap under the umbrella study name Trans-Omics for Precision Dentistry and Early Childhood Caries or TOPDECC (accession: phs002232.v1.p1) and as BioProject 671299 (PRJNA671299; dbGaP: phs002232; https://www.ncbi.nlm.nih.gov/bioproject/671299). The ZOE-pilot data are available as part of BioProject 843091 “ZOE 2.0 pilot study” (PRJNA843091). The IBD data are available at https://ibdmdb.org. The simulation R code is at https://github.com/Hunyong/microbiome2020. The code for the KS procedure is available at https://github.com/Hunyong/microbiome2020/blob/master/Readme_KS_test.Rmd.

## Author Biographies

**Hunyong Cho** was a PhD student at the Department of Biostatistics, Gillings School of Global Public Health at University of North Carolina at Chapel Hill, currently is at Amazon.

**Yixiang Qu** is a PhD student at the Department of Biostatistics, Gillings School of Global Public Health at University of North Carolina at Chapel Hill.

**Chuwen Liu** is a PhD student at the Department of Biostatistics, Gillings School of Global Public Health at University of North Carolina at Chapel Hill.

**Boyang Tang** is a PhD student at the Department of Statistics, University of Connecticut.

**Ruiqi Lyu** is a PhD student at the School of Computer Science, Carnegie Mellon University.

**Bridget Lin** is a PhD student at the Department of Biostatistics, Gillings School of Global Public Health at University of North Carolina at Chapel Hill.

**Jeffrey Roach** is a Senior Scientific Research Associate in Research Computing at University of North Carolina at Chapel Hill.

**M. Andrea Azcarate-Peril** is an Associate Professor of in School of Medicine, and the Director of Microbiome Core at University of North Carolina at Chapel Hill.

**Apoena de Aguiar Ribeiro** is an Associate Professor of Oral Microbiology in the Adam School of Dentistry at University of North Carolina at Chapel Hill.

**Michael I. Love** is an Associate Professor of Genetics, Biostatistics, and Computer Science at the University of North Carolina at Chapel Hill.

**Kimon Divaris** is a Professor in the Division of Pediatric and Public Health the Adam School of Dentistry and the Department of Epidemiology, at the University of North Carolina at Chapel Hill.

**Di Wu** is an Associate Professor in the Department of Biostatistics and Division of Oral Craniofacial Health Science at the University of North Carolina at Chapel Hill.
