# Supplementary material for: Comprehensive evaluation of methods for differential expression analysis of metatranscriptomics data
Source: Brief Bioinform. 2023 Aug 9;24(5):bbad279. doi: 10.1093/bib/bbad279 (PMC10516371; doi:10.1093/bib/bbad279)
Supplement: Microbiome_simulation_Appendix_bbad279 [file microbiome_simulation_appendix_bbad279.pdf]

Supplementary Material for "Comprehensive evaluation of methods for differential expression analysis of metatranscriptomics data" by Hunyong Cho, Yixiang Qu, Chuwen Liu, Boyang Tang, Ruiqi Lyu, Bridget M. Lin, Jeff Roach, M. Andrea Azcarate-Peril, Apoena de Aguiar Ribeiro, Michael I. Love, Kimon Divaris, and Di Wu

June 23, 2023

Contents

S1 Differential expression analysis methods **3**
- S1.1 Log-normal test . . . 3
- S1.2 Logistic Beta test . . . 4
- S1.3 MAST . . . 4
- S1.4 DESeq2 . . . 5
- S1.5 metagenomeSeq . . . 6
- S1.6 ANCOM-BC . . . 7
- S1.7 LEfSe . . . 8
- S1.8 ALDEx2 . . . 8
- S1.9 Kruskal-Wallis test . . . 9
- S1.10 two-part Kruskal-Wallis test, KWII . . . 10
- S1.11 Data scaling and transformation . . . 11

S2 Full description of the three metatranscriptomics datasets **11**

S3 Technical details in semi-parametric simulation **15**

S4 Data generative models **15**

S5 Model-based simulation setup **16**
S5.1 Overview . . . 16
S5.2 Baseline parameters for ZILN-based simulation from ZOE2.0 at the gene level . . . 17
S5.3 Baseline parameters for ZIG-based simulation from ZOE2.0 at the gene level . . . 17
S5.4 Parameter estimation and baseline parameters for ZINB-based simulation from ZOE2.0 at the gene level . . . 17
S5.5 Estimated parameters at the gene level from the two validation datasets 19
S5.5.1 Estimated parameters from the ZOE-pilot data. . . . 19
S5.5.2 Estimated parameters from the IBD data . . . 19
S5.6 Estimated parameters at the level of other microbial features . . . 21
S5.6.1 Parameters for the gene expression of each gene-species combination as the joint gene-species level . . . 21
S5.6.2 Estimated parameters for the total gene expression of each species, at the species marginal level . . . 21

S6 Goodness of fit results **30**
S6.1 Goodness of fit for the ZINB models . . . 30

S7 Parametric model-based simulation results **32**
S7.1 Full results under ZILN models . . . 32
S7.2 Full results under ZINB models . . . 32
S7.2.1 Full results under ZINB models—Sensitivity . . . 32
S7.2.2 Type I error and FDR under ZINB model for mean shift (alternative hypothesis D2) . . . 32
S7.3 Full results under ZIG models . . . 36
S7.3.1 Full results under ZIG models—Sensitivity . . . 36
S7.4 Type I error and FDR results under ZIG model for mean shift (alternative hypothesis D2) . . . 36

S8 Application to the ZOE2.0 data **40**
S8.1 Application to the ZOE2.0 data - gene-species . . . 40
S8.2 Application to the ZOE2.0 data - species . . . 41
S8.3 Application to the ZOE2.0 data - gene profiles . . . 41
S8.4 Summary for the ZOE2.0 results . . . 43

S9 Application to the IBD data **47**
S9.1 Application to the IBD data - gene profiles . . . 47
S9.2 Application to the IBD data - alternative tests . . . 49
S9.3 Summary for the IBD results . . . 50

Details of differential expression analysis methods and data generative models are provided in Supplementary Sections S1–S4, respectively. Supplementary Section S5 entails supplementary information about the parametric simulation set-ups, and Supplementary Section S6 includes the goodness of fit results for the ZINB model. Extensive simulation results are provided in Supplementary Section S7. Additional results of methods' application to the ZOE2.0 data and the IBD data are contained in Supplementary Sections S8 and S9, respectively.

S1 Differential expression analysis methods

In this section, we present each of the DE analysis methods in detail. We first introduce notational conventions. Throughout this material, $Y_{i,g}$ denotes the expression level for the gth gene in the ith cell, $X_i \equiv (1, X_i^D, X_i^B)$ denotes the ith row of the design matrix, or a vector containing the intercept term, a binary disease status, and a binary batch indicator. Different models abusively use the same notation for parameters, as long as there is no confusion; e.g. regression coefficients β_g are commonly used either in the LN model or the LB model, but they are shorthand for β_g^{LN} and β_g^{LB}, respectively.

S1.1 Log-normal test

The Log-normal (LN) test relies on the assumption that the log-transformed expression is normally distributed as in (1). A small positive constant (c) is added to the gene expression to ensure that the log-transformed values are within a feasible range. In this simulation study, 1 is uniformly added to expression levels ($c = 1$).

$$\log_2(Y_{i,g} + c) \sim N(\mu_{i,g}, \sigma_g), \tag{1}$$

where $\mu_{i,g} \equiv X_i^\top \beta_g$ with $\beta_g \equiv (\beta_g^0, \beta_g^D, \beta_g^B)^\top$.
The null and the alternative hypotheses for the gth gene are

- H_0: $\beta_g^D = 0$ and
- H_1: $\beta_g^D \neq 0$.

The test statistic for the gth gene is $T_g^{LN} = \left(\frac{\hat{\beta}_g^D}{se(\hat{\beta}_g^D)}\right)^2$ and follows a χ_1^2 distribution under the null hypothesis asymptotically. The test rejects the null hypothesis if the test statistic is larger than $\chi_1^2(1-\alpha)$, or the $(1-\alpha)$th quantile of the χ^2 distribution with one degree of freedom, where α is the significance level. Alternatively, the individual p-values are obtained as $p_g = 1 - F_{\chi_1^2}(T_g^{LN})$, where $F_d(t)$ is the distribution function of d evaluated at t. The genes with p-values less than α are declared to have a statistically significant association with disease. This test is simply an analysis of covariance (ANCOVA) with an appropriately log-transformed dependent variable, and is easily implemented in most statistical software packages. The testing procedure, after obtaining a test statistic and the corresponding null distribution (e.g., p-values and rejection regions), is identical for all other tested methods, hence will be omitted unless needed.

S1.2 Logistic Beta test

The Logistic Beta model (LB) models relative expressions, $R_{i,g} \equiv Y_{i,g}/\sum_{h=1}^{G} Y_{i,h}$, instead of absolute expressions, $Y_{i,g}$. Because of the sum-to-one constraint of relative expressions, these tests are structurally dependent. However, in microbiome data analyses, the number of tested genes is usually large enough and thus the dependence induced by the compositional structure is negligible.

The LB model is formulated [1] as:

$$R_{i,g} \sim LB(\pi_{i,g}, \mu_{i,g}, \phi_g), \tag{2}$$

where $\pi_{i,g} = \text{expit}(X_i^\top \gamma_g)$ with $\gamma_g = (\gamma_g^0, \gamma_g^D, \gamma_g^B)^\top$, $\mu_{i,g} = \text{expit}(X_i^\top \beta_g)$ with $\beta_g \equiv (\beta_g^0, \beta_g^D, \beta_g^B)^\top$, ϕ_g denotes the dispersion parameter such that $var(R_{i,g}|R_{i,g} > 0) = \mu_{i,g}(1-\mu_{i,g})\phi_g$, and $\text{expit}(\cdot) := \frac{\exp(\cdot)}{\exp(\cdot)+1}$.

Note that this model can be decomposed into two orthogonal models:

$$1(R_{i,g} = 0) \sim \text{Bernoulli}(\pi_{i,g}), R_{i,g}|R_{i,g} > 0 \sim \text{Beta}(\mu_{i,g}, \theta_g), \tag{3}$$

where $1(\cdot)$ is the indicator function, $\mu_{i,g}$ is the mean of the Beta random variable and θ_g is the dispersion parameter. Orthogonality means that the estimate of $\pi_{i,g}$ and those of $\mu_{i,g}$ and θ_g are independent. Consequently, the test statistic can be obtained from these two separately estimated models. The maximum likelihood estimators (MLE) are used for estimation and the `R` package `gamlss` [2] was used to carry out simulations in this study.

The null and the alternative hypotheses for the gth gene are

- H_0: $\beta_g^D = \gamma_g^D = 0$ and
- H_1: Either $\beta_g^D \neq 0$ or $\gamma_g^D \neq 0$.

Either a Wald-type or a likelihood test statistic can be used to test these hypotheses. Because they are asymptotically equivalent, here we only present a Wald test statistic:

$$T_g^{LB} = \left(\frac{\hat{\beta}_g^D}{se(\hat{\beta}_g^D)}\right)^2 + \left(\frac{\hat{\gamma}_g^D}{se(\hat{\gamma}_g^D)}\right)^2, \tag{4}$$

which follows a χ_2^2 distribution under the null hypothesis asymptotically.

If only one of the two parts of LB is estimable, the test statistic is constructed based on the estimable component only and the reference distribution is χ_1^2; e.g., when only the logistic model is estimable, $T_g^{LB} = \left(\frac{\hat{\gamma}_g^D}{se(\hat{\gamma}_g^D)}\right)^2$. The same approach was followed for the other two-part tests, including MAST and the two-part Kruskal-Wallis test.

S1.3 MAST

The "Model-based Analysis of Single-cell Transcriptomics" (MAST) [3] was proposed specifically for differential expression analysis of scRNAseq data. This model, composed of a logistic regression model and a conditional log-normal model, regularizes

parameter estimation and utilizes estimated cellular detection rates (CDR) as covariates as defined below. The model was designed to deal with zero-inflation which is driven by both technical and biological variabilities in scRNAseq data. Though zeros in microbiome sequencing data are believed to be generated mostly by biological reasons, the proportion of zeros is usually greater than that of conventional single-part parametric models such as Poisson, negative binomial, and log-normal. Thus, it is feasible to interrogate the performance of MAST in the context of microbial transcriptomics analysis.

The models in MAST can be summarized as

$$1(Y_{i,g} = 0) \sim \text{Bernoulli}(\pi_{i,g}),\ \log_2(Y_{i,g} + 1) \mid Y_{i,g} > 0 \sim N(\mu_{i,g}, \sigma_g^2), \tag{5}$$

where $\pi_{i,g} = \text{expit}(X_i^\top \gamma_g)$ with $\gamma_g \equiv (\gamma_g^0, \gamma_g^D, \gamma_g^B, \gamma_g^C)^\top$, $\mu_{i,g} = (X_i^\top \beta_g)$ with $\beta_g \equiv (\beta_g^0, \beta_g^D, \beta_g^B, \beta_g^C)^\top$, $X_i \equiv (1, X_i^D, X_i^B, X_i^C)$, and $X_i^C = \frac{1}{n}\sum_{i=1}^n 1(Y_{i,g} > k)$ is the CDR of the ith subject for background expression level k. In this simulation we set $k = 0$.

The parameters are estimated using a Bayesian framework, where γ_g is regularized under a weak informative prior and $1/\sigma_g$ is regularized using an empirical Gamma prior. The `R` package `mast` is available [4].

The null and the alternative hypotheses for the gth gene are

- H_0: $\beta_g^D = \gamma_g^D = 0$ and
- H_1: Either $\beta_g^D \neq 0$ or $\gamma_g^D \neq 0$.

Either a Wald-type or a likelihood test statistic can be used to test these hypotheses. The Wald statistic is

$$T_g^{MAST} = \left(\frac{\hat{\beta}_g^D}{se(\hat{\beta}_g^D)}\right)^2 + \left(\frac{\hat{\gamma}_g^D}{se(\hat{\gamma}_g^D)}\right)^2, \tag{6}$$

with χ_2^2 as its asymptotic null distribution. The testing procedure is exactly the same as that of the LB test once the coefficients and their standard errors are estimated.

S1.4 DESeq2

The DESeq2 [5] method is widely used for differential expression of RNAseq data. The underlying model of DESeq2 is a negative binomial distribution and it uses empirical Bayes for regularization.

The DESeq2 model can be summarized as

$$Y_{i,g} \sim NB(\mu_{i,g}, \theta_g), \tag{7}$$

where $\mu_{i,g} = s_{i,g}\nu_{i,g}$ is the mean parameter, θ_g is the dispersion parameter, $s_{i,g}$ is the size factor, $\nu_{i,g} = \exp(X_i^\top \beta_g)$ with $\beta_g \equiv (\beta_g^0, \beta_g^D, \beta_g^B, \beta_g^C)^\top$, and $X_i \equiv (1, X_i^D, X_i^B, X_i^C)$. The size factor is the parameter with which we adjust the sequencing depth. For our simulation work, we use the median-of-ratios method [6].

The parameters are estimated using maximum likelihood estimation and then θ_g and β_g^D are regularized using an empirical Bayes approach. The R package DESeq2 is available.

The null and the alternative hypotheses for the gth gene are

- H_0: $\beta_g^D = \gamma_g^D = 0$ and
- H_1: Either $\beta_g^D \neq 0$ or $\gamma_g^D \neq 0$.

A Wald test is used to test these hypotheses. The Wald statistic is given as

$$T_g^{DESeq2} = \left(\frac{\hat{\beta}_g^D}{se(\hat{\beta}_g^D)} \right)^2, \tag{8}$$

with χ_1^2 as its asymptotic null distribution. The testing procedure is exactly the same as that of LB test, once the coefficients and their standard errors are estimated. Because DESeq2 cannot accomodate high zero proportions, an extension was recently developed to enable the modeling of a greater number of zeros in the scRNAseq context [7]. In this modified DESeq2 method, namely DESeq2-ZINBWaVE, first the zero-inflation parameter is estimated using the model,

$$Y_{i,g} \sim ZINB(\mu_{i,g}, \theta_g, \pi_{i,g}), \tag{9}$$

and each observation is assigned a weight of the posterior probability of non-zero-inflation,

$$\frac{(1 - \pi_{i,g}) f_{ZINB}(y_{i,g}; \mu_{i,g}, \theta_g, 0)}{f_{ZINB}(y_{i,g}; \mu_{i,g}, \theta_g, \pi_{i,g})},$$

where f_{ZINB} is the corresponding density of the ZINB distribution. For the size factor estimation in DESeq2-ZINBWaVE, we use the positive counts method. Then, the conventional DESeq2 method is applied, as described earlier, including the weights. Whenever there is no ambiguity, "DESeq2" refers to the original method and "DESeq2-ZINBWaVE" to its extension.

S1.5 metagenomeSeq

MetagenomeSeq (MGS) is a differential abundance analysis method for metagenomics data [8] and the corresponding bioconductor package, metagenomeSeq is available. MGS assumes zero-inflated log normal distribution. Furthermore, MGS uses an empirical Bayes shrinkage method for parameter estimation. Hence, MGS shares common modeling approaches with MAST; however, the two approaches are different in a few aspects. First, MAST uses CDR as a controlling factor in the model while MGS does not. Second, MAST provides tests on two parts of the model; i.e., two p-values are obtained from the zero-inflation part and the log-normal part in MAST. However, in MGS, after estimating the two-part model parameters, only the log-difference of the marginal mean is tested and a single p-value is given [9]. In our simulations, the

`fitFeatureModel` function in the `R` package `metagenomeSeq` is used for implementation [10]. Although the MGS test can account for batch effects mathematically, the current `metagenomeSeq` software does not allow batch variables in the model. Thus, only results without batch effects will be reported in the simulation study in Section 4.

S1.6 ANCOM-BC

ANCOM-BC2 is used as a variation of ANCOM [11] that inherits the philosophy of differential ranking (DR) methods and ANCOM but also provides p-values with computationally efficiency. Comparing to an earlier version ANCOM-BC1, this version omits the structural zero detection procedure to avoid known inflated Type I errors.

ANCOM-BC is a differential abundance analysis method for metagenomics data [12]. It shares the philosophy of its predecessor, ANCOM [11], in that it models the ratio of abundances between taxa. However, unlike ANCOM which is a rank-based approach, ANCOM-BC specifies the test statistic and its associated p-value for a large sample. In ANCOM-BC, the observed abundance $Y_{i,g}$ is assumed to be a realization of the unknown abundance $U_{i,g}$ of the whole ecosystem from where the sample is taken with possibly different sampling fraction η_i for each sample. In other words, $E[Y_{i,g}|U_{i,g}] = \eta_i U_{i,g}$, where $U_{i,g}$ is a random variable with mean θ_g^D or θ_g^H, depending on the membership of the sample i to the disease (D) or health (H) group. Of note, ANCOM-BC is not limited to two-group problems and can accommodate multi-group problems. Then it formulates $\log Y_{i,g} = \log \tilde{\eta}_i + \log \theta_{i,g} + \epsilon_{i,g}$, where $\tilde{\eta}_i$ is a slightly-redefined sampling fraction parameter due to the log-transformation, and $E[\epsilon_{i,g}] = 0$.

The hypotheses of ANCOM-BC are

- H_0: $\log \theta_g^D = \log \theta_g^H$ and
- H_1: $\log \theta_g^D \neq \log \theta_g^H$,

which are tested by the test statistic,

$$T_g^{ANCOM-BC} = \frac{\widehat{\log \theta_g^D} - \widehat{\log \theta_g^H} - \widehat{\log \tilde{\eta}}}{\sqrt{\{\hat{\sigma}_g^D\}^2 + \{\hat{\sigma}_g^H\}^2}},$$

where $\widehat{\log \theta_g^A}$ is the estimates of $\log \theta_g^A$, $\{\hat{\sigma}_g^A\}^2$ is the mean squared error for each group $A = D, H$, and $\widehat{\log \tilde{\eta}}$ is the estimate of the bias, $\log \tilde{\eta} \equiv E[\log \theta_g^D - \log \theta_g^H]$. The statistic follows the standard normal distribution per the large sample theory, whereas the authors defined a small sample version without defining the test statistic distribution.

ANCOM-BC does a further procedure of detecting "the structural zero" which is defined to be the absence of a certain taxon in a specific group that is present in another group. Once the structural zero is detected, ANCOM-BC declares that the taxon is differentially abundant, giving $T_g^{ANCOM-BC} = \infty$ and p-value $= 0$. However, since such a procedure often inflates the type-I error significantly in this simulation study, we add another version of ANCOM-BC that declares those sturctural zeros

inconclusive (i.e., p-value = NA). We report the simulations results for second version as "ANCOM-BC2".

S1.7 LEfSe

LEfSe (Linear discriminant analysis Effect Size) is commonly used for differential analysis of metagenomic biomarkers [13]. It assumes that the samples are labeled with a certain group that represents the main biological comparison class of interest. They may also include one or more subgroup labels that indicate within-group classifications where batch effects can be accounted for in simulations. By combining standard tests for statistical significance, with extra tests encoding biological consistency and effect relevance, the method determines the features most likely to explain variations across groups.

There are three steps performed in order: the KW rank-sum test on groups, the pairwise Wilcoxon test between subgroups of different groups, and the LDA on the relevant features. In the first step, the factorial KW rank-sum test is applied to each feature according to the group label; the subgroup label is utilized for further stratifying when that information exists. As a result, only the features that reject the null hypothesis of identical value distribution among groups could be analyzed further. In the second step, the pairwise Wilcoxon test is applied to the extracted features belonging to subgroups of different groups, for testing whether all pairwise comparisons between subgroups in different groups significantly agree with the group level trend. If at least one comparison between subgroups has a p-value greater than the specified threshold, or if the sign of variation is not equal across all comparisons, the pairwise Wilcoxon test is not satisfied for that feature. The first two steps employ non-parametric tests because they are distribution-free approaches and much more robust to the underlying distribution of the data: the only assumption of the Wilcoxon and KW tests is that the distributions in each group are identically shaped with possible differences in the medians. Finally, in the third step, an LDA model is generated, by assigning the remaining features and subgroup labels as the independent variable and the group label as the dependent variables. This step is used to estimate their effect sizes, which are obtained by averaging the differences between group means with the differences between group means along the first linear discriminant axis, which equally weights features' variability and discriminatory power. The LDA score for each biomarker is obtained by computing the logarithm of this value after being scaled and induces the ranking of biomarker relevance, regardless of the absolute values of the LDA score. For robustness, LDA is additionally supported by bootstrapping and subsequent averaging. For implementation of this test, an R function lefser::lefser() is available.

S1.8 ALDEx2

ALDEx2 is a differential abundance analysis method for RNA-seq, 16S rRNA gene sequencing and differential growth datasets [14]. Instead of proposing new model, this

method improved on the data preprocessing, and it uses multiple instances to generate p-values.

If $Y_{i,g}$ denotes the read counts for the gth gene in the ith cell, suppose the intention is to use K instances to generate the p-value. For each sample i, to generate the kth instance, ALDEx2 uses posterior Dirichlet distribution with an uninformative prior of $\frac{1}{2}$ to model the frequency of features with zero counts.

$$\left(Y_{i,1}^{(k)}, Y_{i,2}^{(k)}, \ldots, Y_{i,G}^{(k)}\right) = Dir\left(Y_{i,1} + \frac{1}{2}, Y_{i,2} + \frac{1}{2}, \ldots, Y_{i,G} + \frac{1}{2}\right) \tag{10}$$

where $Dir(\alpha_1, \alpha_2, \ldots, \alpha_G)$ denotes the Dirichlet distribution with paramter $\boldsymbol{\alpha} = (\alpha_1, \ldots, \alpha_K)$. Then, ALDEx2 uses CLR method to centralize the input data.

$$c_{i,g}^{(k)} = log_2\left(Y_{i,g}^{(k)}\right) - \frac{1}{G} log_2\left(\prod_{g=1}^{G} Y_{i,g}^{(k)}\right) \tag{11}$$

Then different models can be applied to the certralized data. Since we have two covariates in the model, we can use the linear model which is formulated as:

$$c_{i,g}^{(k)} \sim N\left(\mu_{i,g}^{(k)}, \sigma_g^{(k)}\right) \tag{12}$$

Where $\mu_{i,g} \equiv X_i^\top \boldsymbol{\beta}_g^{(k)}$ with $\beta_g^{(k)} \equiv \left(\beta_g^{0,(k)}, \beta_g^{D,(k)}, \beta_g^{B,(k)}\right)^\top$.

The null and the alternative hypotheses for the gth gene with kth instance is

- H_0: $\beta_g^{D,(k)} = 0$ and
- H_1: $\beta_g^{D,(k)} \neq 0$.

The test statistic for the gth gene with kth instances $T_g^{LN,(k)} = \left(\frac{\hat{\beta}_g^{D,(k)}}{se(\hat{\beta}_g^{D,(k)})}\right)^2$ and follows a χ_1^2 distribution under the null hypothesis asymptotically. The test rejects the null hypothesis if the test statistic is larger than $\chi_1^2(1-\alpha)$, or the $(1-\alpha)$th quantile of the χ^2 distribution with one degree of freedom, where α is the significance level. Alternatively, the individual p-values with kth instance are obtained as $p_g^{(k)} = 1 - F_{\chi_1^2}(T_g^{LN,(k)})$, where $F_d(t)$ is the distribution function of d evaluated at t. The final p-value for gene g is defined using $p_g = \frac{1}{K}\sum_{k=1}^{K} p_g^{(k)}$. And the genes with p-values less than α are declared to have a statistically significant association with disease.

S1.9 Kruskal-Wallis test

The Kruskal-Wallis (KW) test is equivalent to one-way analysis of variance (ANOVA) on ranks. KW is equivalent to Wilcoxon's rank sum (WRS) test, or Wilcoxon-Mann-Whitney test, for two sample problems and can accomodate comparisons of more than two samples [15]. Although the prototypical KW test was designed without

consideration of covariates, it can be modified to account for possible batch effects [16]:

$$T_g^{KW} \equiv (n-1)\frac{\sum_{i=1}^{n}(\bar{r}_g^{db} - \bar{r}_g^{\cdot b})^2}{\sum_{i=1}^{n}(r_{i,g} - \bar{r}_g^{db})^2}, \tag{13}$$

where $r_{i,g} := \sum_{j=1}^{n}\{1(Y_{i,g} > Y_{j,g}) + \frac{1}{2}(Y_{i,g} = Y_{j,g})\} + 1$ is the rank of the ith subject's gth gene among n subjects, $\bar{r}_g^{db} := \frac{\sum_{i=1}^{n} r_{i,g}1(X_i^D=d, X_i^B=b)}{\sum_{i=1}^{n} 1(X_i^D=d, X_i^B=b)}$, $\bar{r}_g^{d\cdot} := \frac{\sum_{i=1}^{n} r_{i,g}1(X_i^D=d)}{\sum_{i=1}^{n} 1(X_i^D=d)}$, and $\bar{r}_g^{\cdot\cdot} := \frac{1}{n}\sum_{i=1}^{n} r_{i,g}$.

The null and the alternative hypotheses for the gth gene are

- H_0: The ranked expression levels are independent of the phenotypic outcome controlling for batch effects,
- H_1: The complement of H_0.

The exact and approximate distributions of the statistic under the null hypothesis can be obtained through analysis or resampling [16]. However, when disease and batch strata are large, the statistic converges to χ_1^2. Based on the null distribution, p-values are obtained for each gene.

For implementation of this test, the `R` function `coin::kruskal_wallis()` [17] is available. The `coin` package function allows only a single batch variable.

S1.10 two-part Kruskal-Wallis test, KWII

Nonparametric tests such as KW and Wilcoxon's rank-sum (WRS) test have minimal distributional assumptions. The lack of model-induced information often results in lack of power. While zero-inflation is a well-known characteristic of microbiome sequencing data, explicitly modeling the proportion of zeros can enhance the power in detecting differential expression. This additional assumption can be integrated into the nonparametric models using a two-part model framework [18]. Of note, the LB test and the MAST are also two-part models but they are fully parametric. Nonparametric two-part models have been used in other 'omics applications [19] and microbiome [20] data analyses. The binary part of these nonparametric models has been modeled using a conventional proportion test where no covariates are allowed. To incorporate covariate information in the binary model, a logistic regression model can be used. The KW or the WRS test can be used as the nonzero model–to allow for the inclusion of covariates in the model, the modified KW test can be used. In this paper we combine a logistic regression model and a KW test and name it two-part KW test. The binary component of the model is the same as that of LB model, i.e. Equation (3). The nonzero component's test statistic is derived based only on subjects with non-zero gene expressions and has the same formula given in KW test, i.e. Equation (13).

The p-values can be obtained by combining two χ_1^2 statistics derived from each component:

$$T_g^{KWII} := W_g^a + W_g^b,$$

where W_g^a is either a Wald test statistic ($\left(\frac{\hat{\beta}_g^D}{se(\hat{\beta}_g^D)}\right)^2$) or a likelihood ratio statistic (two times the difference of log-likelihood of logistic regression models), and W_g^b has the same form as Equation 13. The test statistic, T_g^{KWII} follows a χ_1^2 distribution under the null hypothesis asymptotically.

S1.11 Data scaling and transformation

TPM is defined as RPK divided by the sample sum of RPKs times a constant ($TPM_{i,g} = c\frac{RPK_{i,g}}{\sum_{g=1}^{n_{genes}} RPK_{i,g}}$) where the constant, c, was chosen to reflect the actual scale of the RPKs, or $c = 5(20)$ million in the ZOE-pilot and ZOE2.0 studies. Arcsine-transformation is defined as $c\frac{2}{\pi}\arcsin(\sqrt{TPM/c})$. Note that the Beta distribution is applied to compositional data and thus RPK and TPM data are equivalent in this context.

S2 Full description of the three metatranscriptomics datasets

To understand and characterize the distributional features of metatranscriptomics data, we leverage three datasets generated in two recent studies involving the human microbiome. We follow a systematic data harmonization approach to obtain gene-level expression data in RPKs as the summed (or marginalized) RPK over all species per gene before DE analysis. To expand the relevance of this comparative model evaluation, we consider additional aspects of microbial activity—i.e., expression at the gene-species level and the species level (“gene-species data”) as aggregation over all genes in individual species (“species marginal data”).

The first two datasets (namely, ZOE2.0 including 297 participants and ZOE-pilot including 116 participants) were generated in a molecular epidemiologic study of early childhood caries (ECC; defined as dental cavities in children under the age of 6) [21] called Zero-Out Early Childhood Tooth Decay (ZOE) [22, 23]. In that study, investigators tested the associations between features of the supragingival oral microbiome (i.e., “dental plaque”) and the prevalence of clinically-determined ECC. Of note, ECC prevalence was similar in the two ZOE waves, i.e., ZOE2.0: 49(147/297) and ZOE-pilot: 50The study included known batch effects due to the process of clinical sample collection and microbiome sequencing at different dates. For details, estimates of taxonomic composition, gene family, path abundance, and path coverage were produced from the obtained and filtered reads using HUMAnN2 [24]. The resulting reads were scaled into reads-per-kilobase (RPK). We considered additional pre-processing methods including transcript-per-kilobase-million (TPM) and arcsine in Section 5. The total number of gene-species combinations (in that counts are at the per-gene-per-species level, also called joint ”gene-species”) in ZOE2.0 metatranscriptomics is 535,299; there are 204 distinct species, and 402,937 distinct genes. In the ZOE-pilot dataset, there are 439,872 gene-species, 185 distinct species, and 342,004 distinct genes.

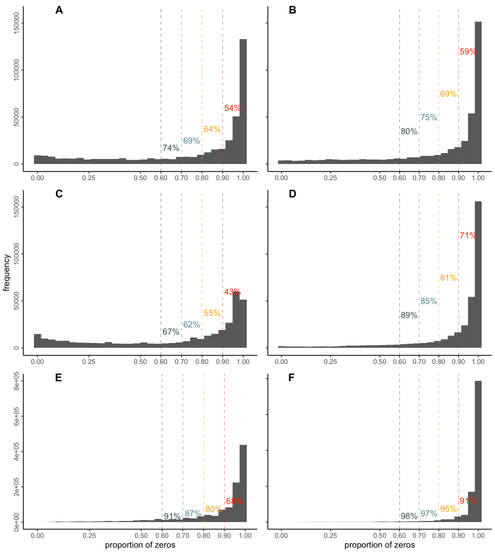

Fig. S1: Histogram of zero-proportions at the gene-level in the metagenomics (LEFT) and the metatranscriptomics (RIGHT) data of the ZOE2.0 (Row 1), the ZOE-pilot (Row 2), and the IBD (Row 3) studies. A. ZOE2.0 metagenomics; B. ZOE2.0 metatranscriptomics; C. ZOE-pilot metagenomics; D. ZOE-pilot metatranscriptomics; E. IBD metagenomics; F. IBD metatranscriptomics; Numbers on the histogram represent the proportion of genes of which the zero proportion is greater than or equal to the cutoff values, or the vertical bars left to the numbers.

Total RPKs per sample is on average 13,053,428 in ZOE2.0 and 2,815,749 in ZOE-pilot. The RPKs are rescaled by dividing by the total RPKs per sample and then multiplying by 4.0 million in ZOE2.0 and 3.4 million in ZOE-pilot, to make the total expression level for each subject to be 10 times the number of genes. This is a scaled version of TPM-normalized data. In this article, for notational convenience, this scaled version of TPM is referred to as TPM. ZOE2.0 data are available via (https://www.ncbi.nlm.nih.gov/bioproject/671299 and ZOE-pilot data are available via (https://www.ncbi.nlm.nih.gov/bioproject/843091.

		ZOE-pilot	ZOE2.0	IBD
genes	RNA	87.9%	80.4%	96.3%
	DNA	68.0%	75.0%	87.8%
species	RNA	80.6%	73.8%	82.8%
	DNA	66.0%	68.8%	82.7%

Tab. S1: Percentage of zeros at the entry level

The third dataset (namely, the IBD data) was generated in the context of a

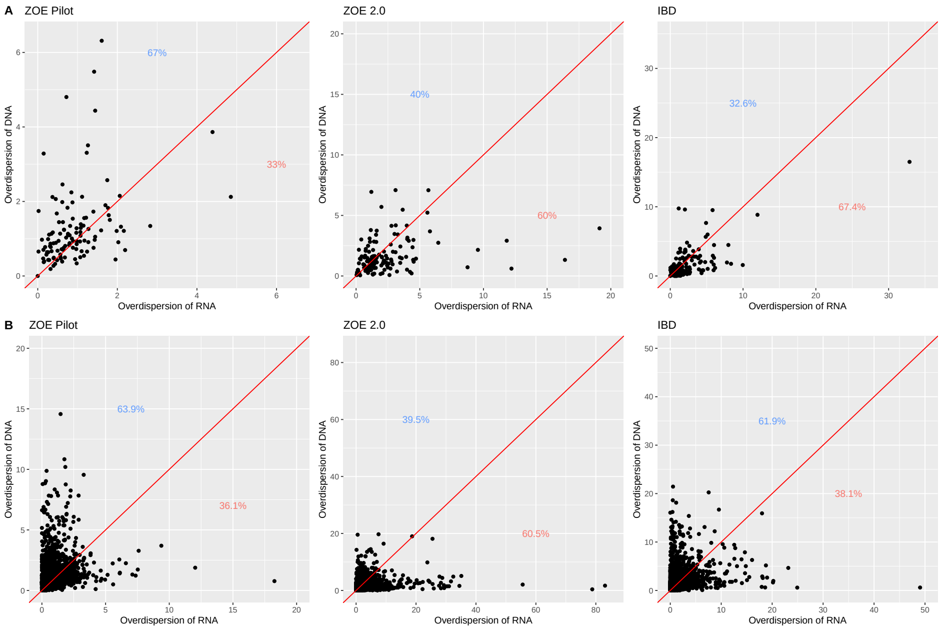

Fig. S2: Scatterplot of overdispersion (θ, defined as the variance divided by the square of the mean) for the non-zero values in the metagenomics (DNA, y axis) and metatranscriptomics (RNA, x axis) data from ZOE-pilot, ZOE2.0 and IBD at both the A) species and the B) genes level. The red line denotes the diagonal line $y = x$. Numbers on the upper and lower side of diagonal line in A (and B) represent the proportion of species (or genes in B) for which overdispersion in DNA is higher/lower than that in RNA. The genes showed in the plot are 1% of the total genes, randomly selected. Each dot is a feature, that is, a species in A, and a gene in B. Overdispersion parameters were estimated under the ZILN model.

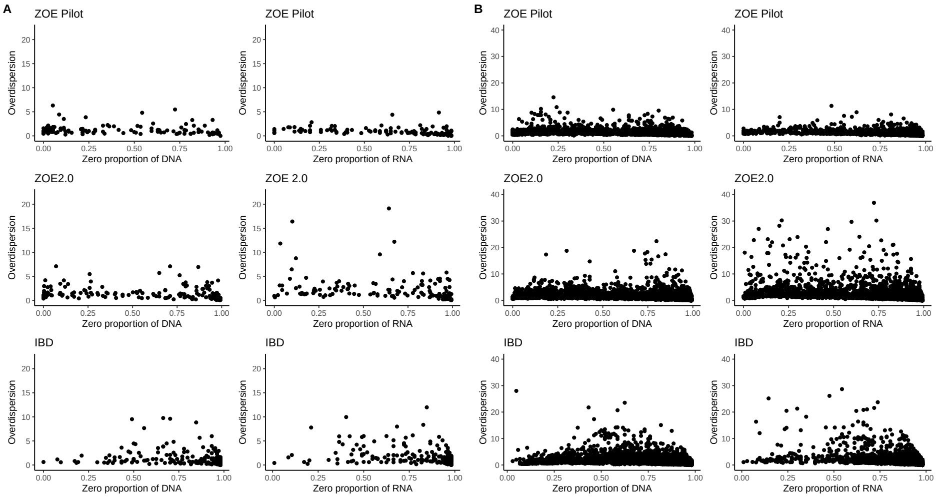

Fig. S3: Scatterplot of zero proportions and overdispersion in the metagenomics and metatranscriptomics data in the ZOE-pilot, ZOE2.0 and IBD studies at the A) species and B) gene level. Each dot is a feature, that is, a species in A, and a gene in B. The genes shown in the plot are 1% of the total genes, randomly selected.

recent study of the gut microbial ecosystem and its association with Inflammatory Bowel Diseases (IBD) [25], including metatranscriptomes obtained from fecal samples of 132 subjects during a one-year period, with repeated measurements of the same participants. The generated taxonomic and functional profiles are publicly available (`http://huttenhower.sph.harvard.edu/biobakery`). The dataset includes a total of 1,595 metagenomic and 818 metatranscriptomic samples. We focus on the cross-sectional features of the metatranscriptomics data distribution, and thus we only examined the baseline information, or the first visit data, including a sample of 104 participants. We further dichotomized participants' disease status as IBD (i.e., 50 Crohn's disease and 26 ulcerative colitis cases) versus non-IBD (i.e., 28 'control' participants). Clinic location was considered as a batch effect in that study, and thus was employed in our analyses after dichotomization (the pediatric versus the adult cohorts). The average proportion of zeros per gene in the IBD metatranscriptomics data is 96.3%, while that in the metagenomics data is 87.8% consistent with the trend of higher zero proportions in metatranscriptomics comapred to metagenomics data. Web Figure 1 illustrates the higher zero proportion in the metatranscriptomes over the metagenomes: 91% of genes have zero proportion $\geq$ 90% in the metatranscriptomics data, compared to 69% in the metagenomics data. These data are available in a compositional format, wherein gene expression data sum up to one over genes and species for each participant.

The overall proportion of zeros in these three datasets at the matrix level is summarized in Table S1. Metatranscriptomics data at the gene level has an overall higher

proportion of zeros compared to metatranscriptomics data at the species level and metagenomics data.

Regarding the overdispersion of data, Web Figure S2 illustrates the relationship between the estimated overdispersion of the metagenomics data and that of the metatranscriptomics data, at the species and the gene levels, for each of the three datasets. Overdispersion in metatranscriptomics data is on average lower than in metagenomics data in (1) ZOE-pilot species level, (2) ZOE-pilot gene level, and (3) IBD gene level data. However, overdispersion in metatranscriptomics data is higher than in metagenomics data in the other three combinations, which are (1) ZOE2.0 species level (2) ZOE2.0 gene level, and (3) IBD species level. Web Figure S3 illustrates the relationship between the overdispersion and the proportion of zeros for a set of randomly selected genes.

S3 Technical details in semi-parametric simulation

In the semi-parametric simulation, gene effect sizes were introduced to participants in the disease groups only, as follows:

Let $(\delta_{\mu,g}, \delta_{\pi,g})$ be the effect size assigned to a signal gene g. Then $\delta_{\pi,g}$ is first applied to flip a certain number of zeros to non-zeros or non-zeros to zeros, and then $\delta_{\mu,g}$ is applied to rescale the non-zero values. Let $\hat{\pi}_{g,A}$ denote the sample zero proportion of the disease group $A =$D, H. Also, let $\hat{\pi}_{g,D}(\delta_\pi) = (\text{expit}(\text{logit}\hat{\pi}_{g,D} + \delta_\pi)$ denote the differentiated zero proportion of gene g in the disease group D, where $\text{expit}(x) = \frac{\exp(x)}{\exp(x)+1}$ and $\text{logit}(x) = \log(\frac{x}{1-x})$. Let $Z_{g,D}$ be a random realization from the binomial distribution with n_D trials and the success probabilities $\hat{\pi}_{g,D}(\delta_\pi)$, where n_D is the sample size of the disease group D. The health group counterparts are defined similarly: $\hat{\pi}_{g,H}(\delta_\pi) = (\text{expit}(\text{logit}\hat{\pi}_{g,H} - \delta_\pi)$ is the differentiated zero proportion, and $Z_{g,H}$ is a binomial random draw with n_H trials. If $Z_{g,D} >= n_A\hat{\pi}_{g,A}$, $Z_{g,D} - n_A\hat{\pi}_{g,A}$ many non-zero-expressed subjects are randomly chosen and are replaced with zeros. Contrary, if $Z_{g,D} < n_A\hat{\pi}_{g,A}$, each of the randomly chosen $n_A\hat{\pi}_{g,A} - Z_{g,D}$ many zero-expressed subjects are given the expression values of one of the randomly chosen non-zero subjects. Once the π-disease effects are manipulated, the non-zero expression values, $Y_{g,i} > 0$, are transformed into $\exp(\log(Y_{g,i}) \pm \delta_\mu)$ with $+$ $(-)$ for i in group D (H). Note that the signs of (δ_μ, δ_π) are preserved, so that for some genes δ_μ (δ_π) is positive while for other genes it can be negative.

S4 Data generative models

The zero-inflated log-normal model is a mixture of log-normal distribution with a point mass at zero. The density is given as

$$f_{ZILN}(y) = \pi \ 1(y = 0) + (1 - \pi) \ \varphi(y, \mu, \mu\theta)1(y > 0), \tag{14}$$

where $\varphi(x, \mu, \sigma^2)$ is the log-normal density at x with mean μ and variance σ^2, π is the zero-inflation parameter, or $\pi \equiv \Pr(Y = 0)$, μ is the non-zero mean parameter (i.e.

$\mu \equiv E[Y|Y>0]$), and θ is the over-dispersion parameter so that $var[Y|Y>0] = \mu^2\theta$

ZINB is an extension of the negative binomial distribution and is widely used to model count data with excess zeros. In many real world applications, if more zeros are observed than the negative binomial distribution assumes, the zero-inflated negative binomial distribution is suitable and has in fact become one of the most commonly used methods in count data analysis [26], including omics data analyses involving scRNAseq [27, 7] and microbiome [28, 29].

ZINB without covariates has three parameters, $(\mu, \theta, \pi)^\top$, with the following density:

$$f_{ZINB}(y) = \pi\ 1(y=0) + (1-\pi) \binom{y + \frac{1}{\theta} - 1}{y} \frac{(\mu\theta)^y}{(1+\mu\theta)^{y+1/\theta}}, \tag{15}$$

$y = 0, 1, 2, ...$, where π is the zero-inflation parameter, μ is the mean parameter assuming no zero-inflation (i.e. $E[Y] = \mu(1-\pi)$), and θ is the over-dispersion parameter such that $var[Y] = \mu^2\pi(1-\pi) + (1-\pi)(\mu + \mu^2\theta)$.

We use the same notation $\xi \equiv (\mu, \theta, \pi)$ for each generative model as long as there is no ambiguity, and use a superscript denoting the model if distinction is needed.

The zero-inflated Gamma model is a mixture of a Gamma distribution and a point mass at zero. The density is given as

$$f_{ZIG}(y) = \pi\ 1(y=0) + (1-\pi)\ \frac{y^{\mu/\theta - 1} e^{-y/\theta}}{\Gamma(\mu/\theta)\theta^{\mu/\theta}} 1(y>0), \tag{16}$$

where π is the zero-inflation parameter or $\pi \equiv \Pr(Y=0)$, μ is the non-zero mean parameter (i.e. $\mu \equiv E[Y|Y>0]$), and θ is the over-dispersion parameter so that $var[Y|Y>0] = \mu^2\theta$.

S5 Model-based simulation setup

S5.1 Overview

To comprehensively evaluate the performance of all available methods, we considered two simulation approaches, including fully parametric (Simulation I)and semi-parametric (Simulation II). In Simulation I, three generative models (zero-inflated log normal or ZILN and two more) were used with a comprehensive set of parameters that ranges over the most of the parameter estimates of the example data. An extensive range of parameter sets is used to mitigate the potential model-misspecification, while we provide goodness of fit measures for all generative models. In Simulation II, genes and their expression levels are sampled from the example data followed by artificial insertion (i.e., "spiking") of disease effects. The second simulation serves a validation of Simulation I, and, at the same time, helps evaluate the robustness of the tested DE methods across different datasets. In what follows, we briefly describe how the two simulations were done. These analyses were done using `R` 4.0.3.

No.	μ	θ	π
B01-03	1, 10, 50	0.5	0.3
B04-06	1, 10, 50	2.0	0.3
B07-09	1, 10, 50	0.5	0.6
B10-12	1, 10, 50	2.0	0.6
B13-15	1, 10, 50	0.5	0.65
B16-18	1, 10, 50	2.0	0.65
B19-21	1, 10, 50	0.5	0.7
B22-24	1, 10, 50	2.0	0.7
B25-27	1, 10, 50	0.5	0.75
B28-30	1, 10, 50	2.0	0.75
B31-33	1, 10, 50	0.5	0.8
B34-36	1, 10, 50	2.0	0.8
B37-39	1, 10, 50	0.5	0.85
B40-42	1, 10, 50	2.0	0.85
B43-45	1, 10, 50	0.5	0.9
B46-48	1, 10, 50	2.0	0.9
B49-51	1, 10, 50	0.5	0.95
B52-54	1, 10, 50	2.0	0.95

Tab. S2: Baseline ZILN parameters

S5.2 Baseline parameters for ZILN-based simulation from ZOE2.0 at the gene level

The baseline parameters for ZILN models are chosen as shown in (Web Table S2).

S5.3 Baseline parameters for ZIG-based simulation from ZOE2.0 at the gene level

The parameter estimates for the ZIG model are identical to those of the ZILN model and for this reason are not presented. The baseline parameters for ZIG models are chosen as shown in (Web Table S3).

S5.4 Parameter estimation and baseline parameters for ZINB-based simulation from ZOE2.0 at the gene level

The estimated parameters of the ZINB from ZOE2.0 genes are in (Web Table S4). The baseline parameters, disease effects, and batch effects for ZINB models are chosen (Web Table S5) based on the parameter estimates from the ZOE2.0 data (Web Figure S4).

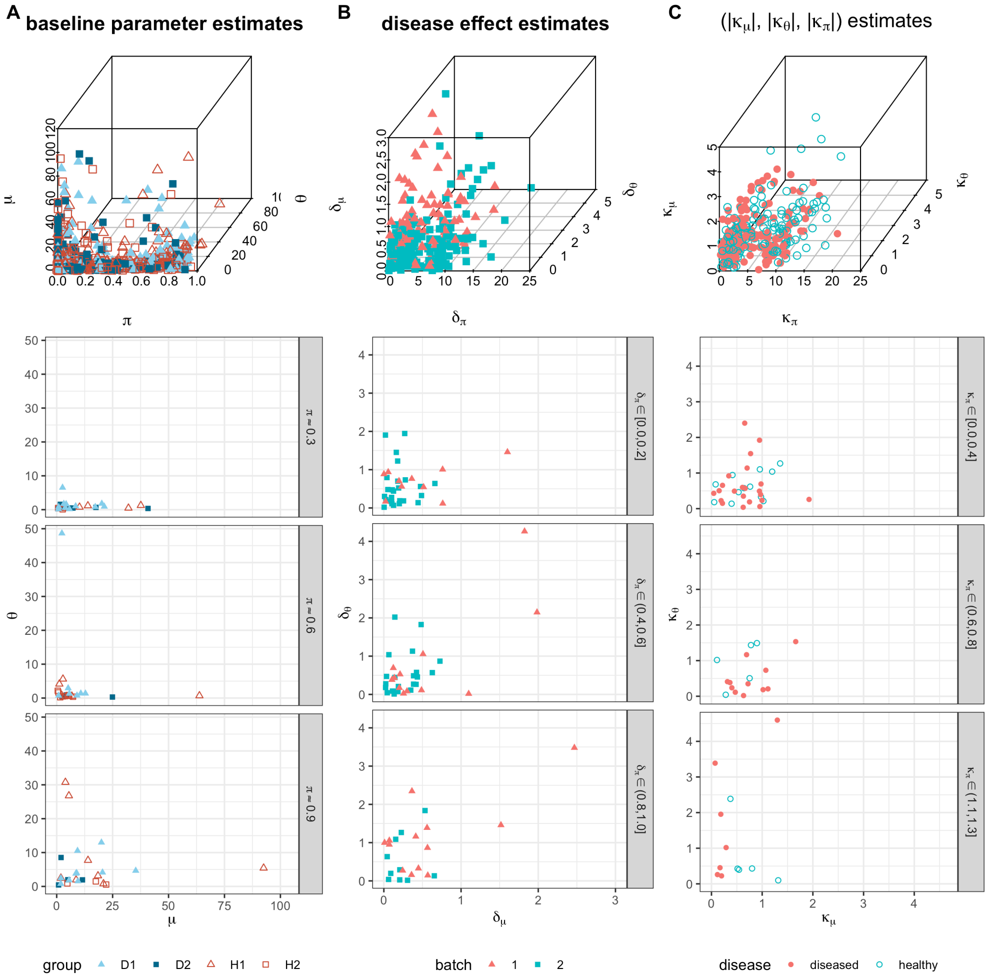

Fig. S4: ZINB-parameter estimates from the ZOE2.0 data. (A) baseline parameter estimates (TOP: $\pi > 0.8$, MIDDLE: $\pi \in (0.4, 0.8]$, BOTTOM: $\pi \leq 0.4$), (B) disease effect parameter estimates, (C) batch effect parameter estimates. The quartiles of the estimated parameters are provided in Web Table S4.

No.	μ	θ	π
B01-03	1, 10, 50	0.5	0.3
B04-06	1, 10, 50	2.0	0.3
B07-09	1, 10, 50	0.5	0.6
B10-12	1, 10, 50	2.0	0.6
B13-15	1, 10, 50	0.5	0.65
B16-18	1, 10, 50	2.0	0.65
B19-21	1, 10, 50	0.5	0.7
B22-24	1, 10, 50	2.0	0.7
B25-27	1, 10, 50	0.5	0.75
B28-30	1, 10, 50	2.0	0.75
B31-33	1, 10, 50	0.5	0.8
B34-36	1, 10, 50	2.0	0.8
B37-39	1, 10, 50	0.5	0.85
B40-42	1, 10, 50	2.0	0.85
B43-45	1, 10, 50	0.5	0.9
B46-48	1, 10, 50	2.0	0.9
B49-51	1, 10, 50	0.5	0.95
B52-54	1, 10, 50	2.0	0.95

Tab. S3: Baseline ZIG-parameters

S5.5 Estimated parameters at the gene level from the two validation datasets

To add to our understanding of metatranscriptomics data distributions generated under realistic conditions, we followed the same procedures to estimate the parameters using the ZOE-pilot and the IBD data. Because the ZILN model is the main focus of our simulation study and the expression of the IBD data was only provided in a compositional form, we use these validation data to estimate the ZILN model parameters only. In the ZILN model, μ is the only parameter that is affected by scale transformations and most test results are thus invariant to scale transformations except for the NB- and the ZINB-based tests. The estimated parameters of the ZILN (and ZIG) models from the validation data—the ZOE-pilot and the IBD data—are presented here.

S5.5.1 Estimated parameters from the ZOE-pilot data.

Web Figure S5 and Web Table S6 illustrate the distribution of the ZILN parameter estimates in the ZOE-pilot data.

S5.5.2 Estimated parameters from the IBD data

Web Figure S6 and Web Table S7 illustrate the distribution of the ZILN parameter estimates in the IBD data.

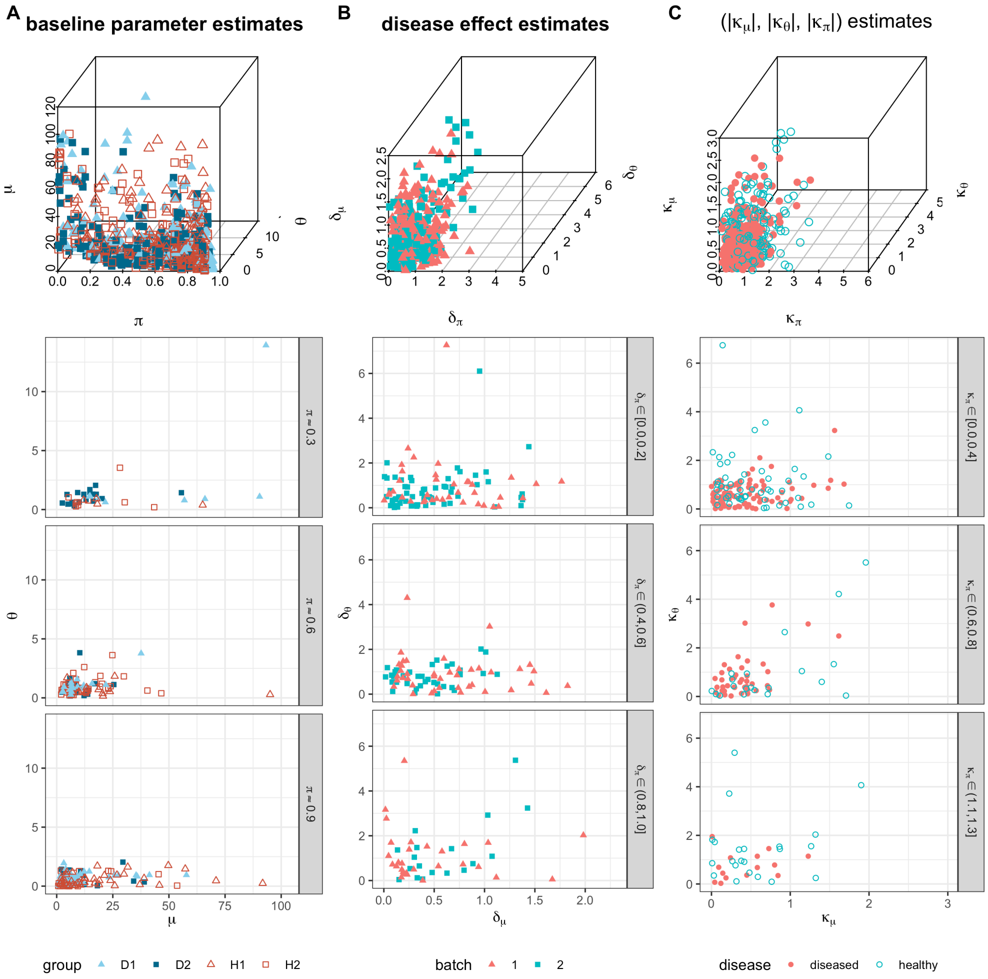

Fig. S5: The ZILN parameter estimates for genes in the ZOE-pilot data
Column A: parameter estimates of baseline ZILN distributions from the ZOE-pilot data with the 3-dimensional scatter plot on the top row and each of the subsequent rows representing π estimates being within 0.03 from 0.9, 0.6, and 0.3.
Column B: disease effect estimates based on ZILN models from the ZOE-pilot data in absolute values $(|\delta_\mu|, |\delta_\theta|, |\delta_\pi|)$
Column C: batch effect estimates based on ZILN models from the ZOE-pilot data in absolute values $(|\kappa_\mu|, |\kappa_\theta|, |\kappa_\pi|)$. The quartiles of the estimated parameters are provided in Web Table S6.

parameters	1Q	2Q	3Q
μ	0.80	2.60	8.00
θ	0.40	0.80	2.90
π	0.00	0.10	0.63
δ_μ	0.10	0.30	0.60
δ_θ	0.20	0.50	1.10
δ_π	0.50	1.30	6.60
κ_μ	0.40	0.90	1.40
κ_θ	0.30	0.60	1.30
κ_π	1.20	2.80	8.90

Tab. S4: The ZINB parameter estimate distribution of genes in the ZOE2.0 data

S5.6 Estimated parameters at the level of other microbial features

S5.6.1 Parameters for the gene expression of each gene-species combination as the joint gene-species level

The distribution of the ZILN parameter estimates of the gene-species combinations in the ZOE2.0 data are shown (Web Figure S7 and Web Table S8).

The distribution of the ZINB parameter estimates of the gene-species combinations in the ZOE2.0 data are shown (Web Figure S8 and Web Table S9).

S5.6.2 Estimated parameters for the total gene expression of each species, at the species marginal level

Web Figure S9 and Web Table S10 illustrate the distribution of the ZILN parameter estimates of species in the ZOE2.0 data.

Web Figure S10 and Web Table S11 illustrate the distribution of the ZINB parameter estimates of species in the ZOE2.0 data.

Estimated parameters (Web Figure S9) of gene expression distribution for species are well covered by the parameter sets in Web Table S2.

Estimated parameters (Web Figure S10) of gene expression distribution for species can be mostly covered by the parameter sets in Web Table S5 except when π is large and the overdispersion parameter θ is very irregular.

No.	μ	θ	π
B01-03	1, 10, 50	1	0.3
B04-06	1, 10, 50	5	0.3
B07-09	1, 10, 50	1	0.6
B10-12	1, 10, 50	5	0.6
B13-15	1, 10, 50	1	0.65
B16-18	1, 10, 50	5	0.65
B19-21	1, 10, 50	1	0.7
B22-24	1, 10, 50	5	0.7
B25-27	1, 10, 50	1	0.75
B28-30	1, 10, 50	5	0.75
B31-33	1, 10, 50	1	0.8
B34-36	1, 10, 50	5	0.8
B37-39	1, 10, 50	1	0.85
B40-42	1, 10, 50	5	0.85
B43-45	1, 10, 50	1	0.9
B46-48	1, 10, 50	5	0.9
B49-51	1, 10, 50	1	0.95
B52-54	1, 10, 50	5	0.95

Tab. S5: Baseline ZINB-parameters

parameters	1Q	2Q	3Q
μ 5.30	10.80	25.90	
θ 0.40	0.70	1.10	
π 0.48	0.72	0.86	
δ_μ 0.20	0.40	0.70	
δ_θ0.30	0.70	1.30	
δ_π 0.20	0.50	0.90	
κ_μ 0.20	0.40	0.70	
κ_θ0.20	0.60	1.10	
κ_π 0.30	0.70	1.20	

Tab. S6: The ZILN parameter estimate distribution of genes in the ZOE-pilot data

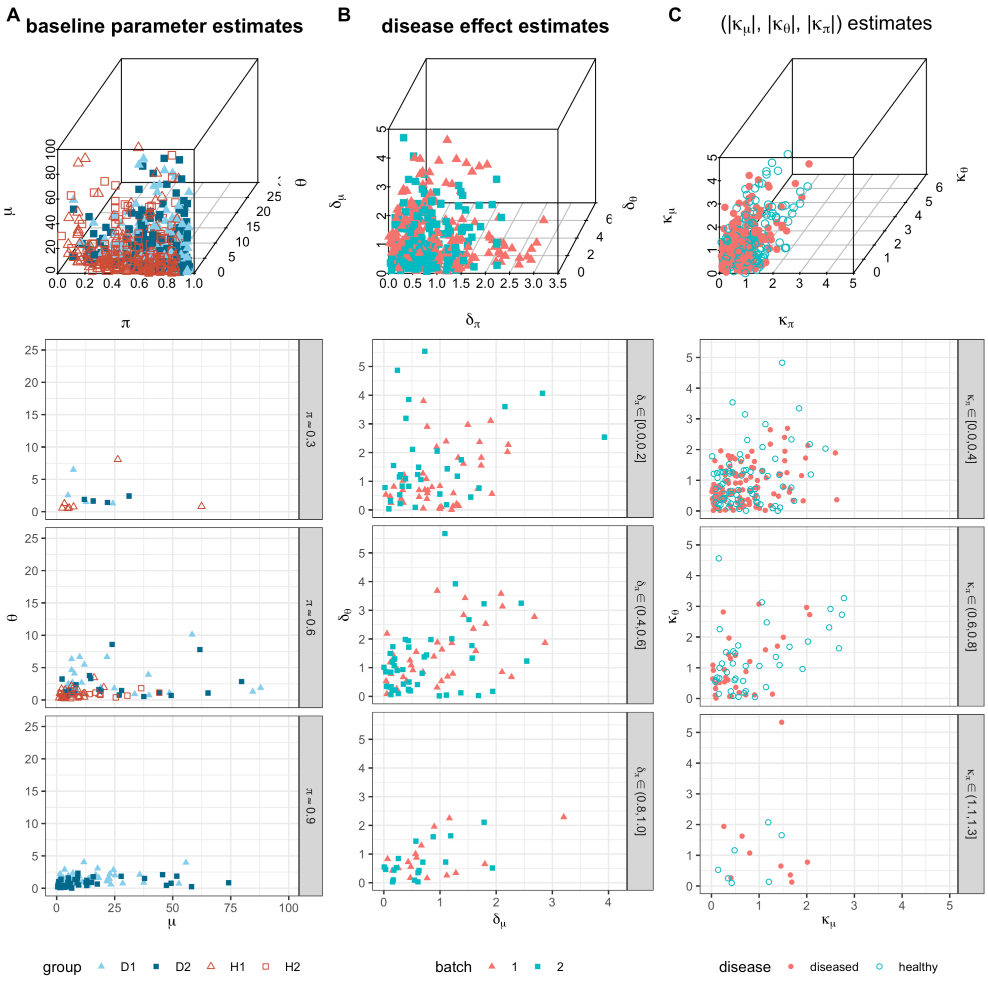

Fig. S6: The ZILN parameter estimates for genes in the IBD data.
Column A: parameter estimates of baseline ZILN distributions from the IBD data with the 3-dimensional scatter plot on the top row and each of the subsequent rows representing π estimates being within 0.03 from 0.9, 0.6, and 0.3.
Column B: disease effect estimates based on ZILN models from the IBD data in absolute values $(|\delta_\mu|, |\delta_\theta|, |\delta_\pi|)$
Column C: batch effect estimates based on ZILN models from the IBD data in absolute values $(|\kappa_\mu|, |\kappa_\theta|, |\kappa_\pi|)$. The quartiles of the estimated parameters are provided in Web Table S7.

parameters	1Q	2Q	3Q
μ	0.00	0.10	0.20
θ	0.50	1.00	1.80
π	0.56	0.75	0.86
δ_μ	0.30	0.70	1.30
δ_θ	0.40	0.90	1.60
δ_π	0.30	0.60	1.00
κ_μ	0.30	0.60	1.00
κ_θ	0.30	0.80	1.40
κ_π	0.30	0.50	0.80

Tab. S7: The ZILN parameter estimate distribution of genes in the IBD data

parameters	1Q	2Q	3Q
μ	3.40	7.70	21.50
θ	0.70	1.10	1.70
π	0.39	0.67	0.83
δ_μ	0.10	0.20	0.50
δ_θ	0.20	0.50	0.90
δ_π	0.20	0.30	0.50
κ_μ	0.30	0.60	1.00
κ_θ	0.30	0.70	1.30
κ_π	0.40	0.70	1.30

Tab. S8: The ZILN parameter estimate distribution of gene-species combinations in the ZOE2.0 data

parameters	1Q	2Q	3Q
μ	2.00	5.90	16.70
θ	0.40	0.90	2.10
π	0.00	0.37	0.71
δ_μ	0.10	0.30	0.60
δ_θ	0.20	0.50	1.20
δ_π	0.30	0.80	3.30
κ_μ	0.40	0.80	1.30
κ_θ	0.30	0.70	1.40
κ_π	0.70	1.60	6.70

Tab. S9: The ZINB parameter estimate distribution of gene-species combinations in the ZOE2.0 data

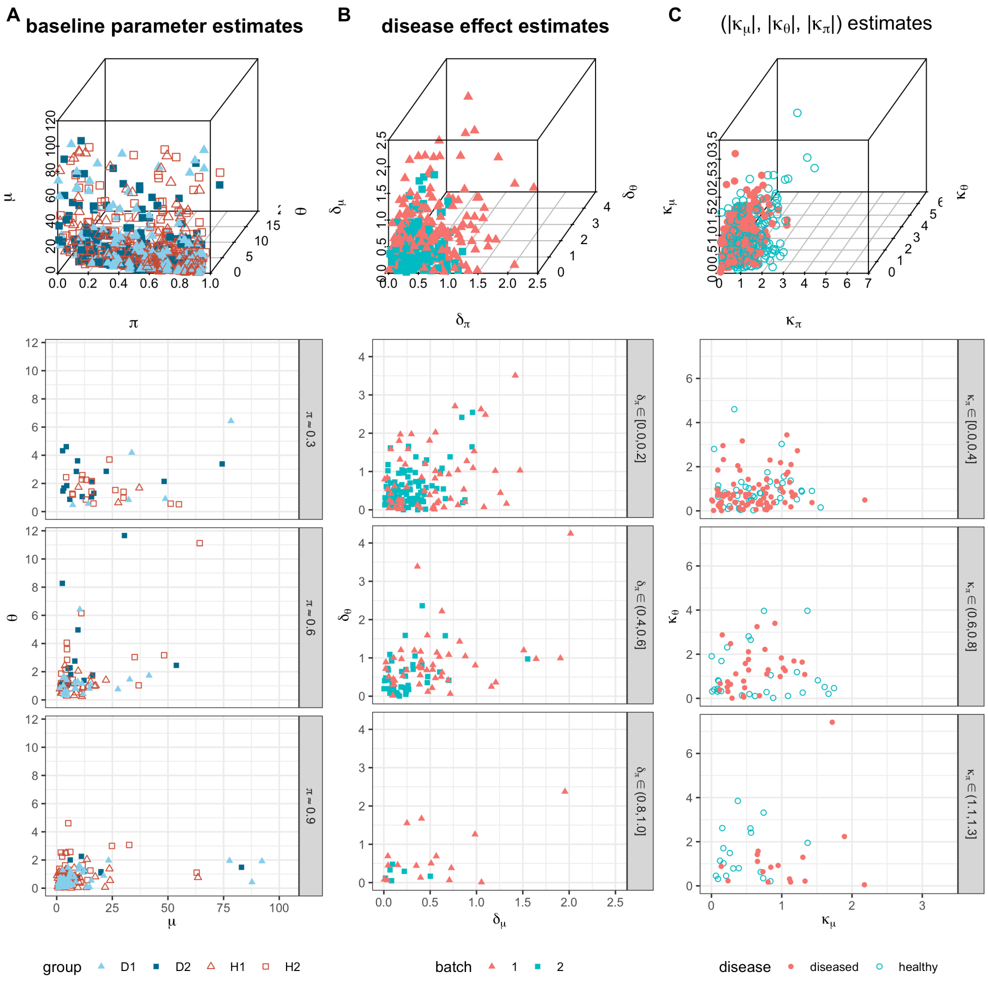

Fig. S7: The ZILN parameter estimates for gene-species combination
Column A: parameter estimates of baseline ZILN distributions from the ZOE2.0 data with the 3-dimensional scatter plot on the top row and each of the subsequent rows representing π estimates being within 0.03 from 0.9, 0.6, and 0.3.
Column B: disease effect estimates based on ZILN models from the ZOE2.0 data in absolute values $(|\delta_\mu|, |\delta_\theta|, |\delta_\pi|)$
Column C: batch effect estimates based on ZILN models from the ZOE2.0 data in absolute values $(|\kappa_\mu|, |\kappa_\theta|, |\kappa_\pi|)$. The quartiles of the estimated parameters are provided in Web Table S8.

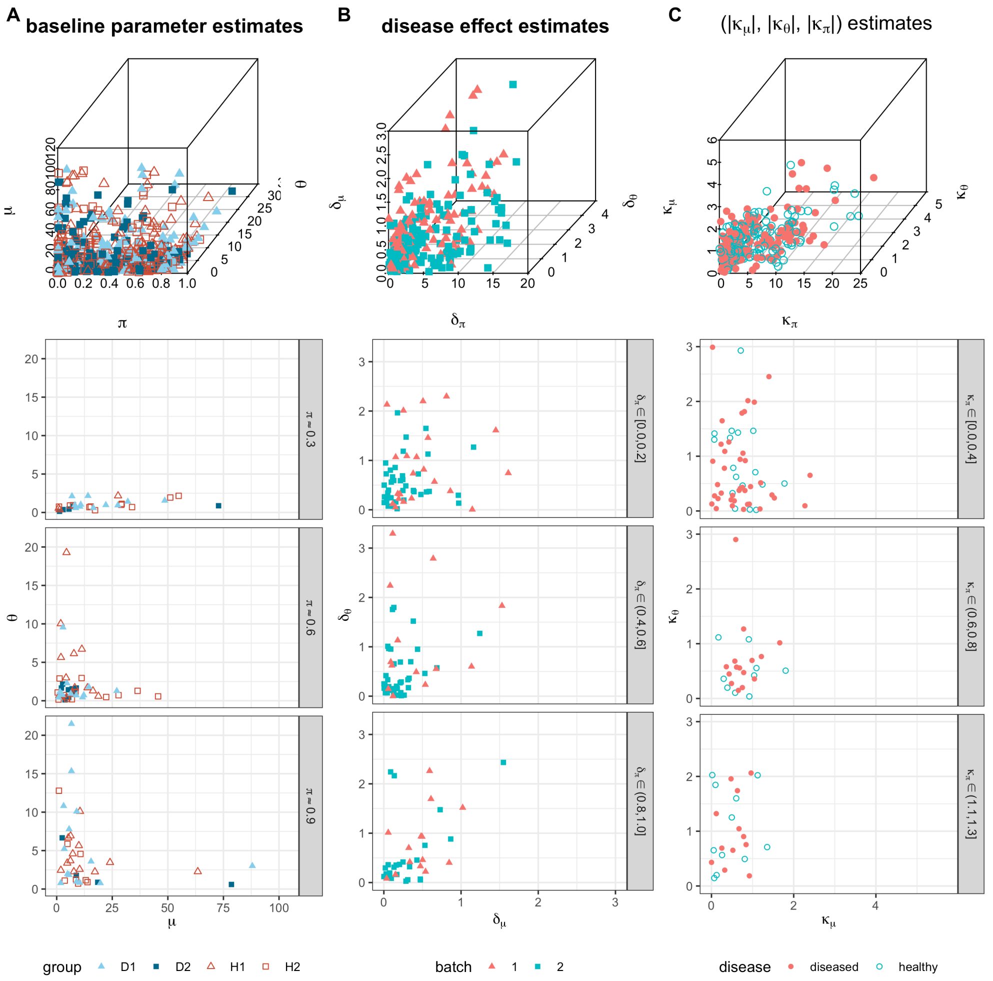

Fig. S8: ZINB parameter estimates for gene-species combinations
Column A: parameter estimates of baseline ZINB distributions in the ZOE2.0 data presented with a 3-dimensional scatter plot on the top row and each of the subsequent rows represents π estimates being within 0.03 from 0.9, 0.6, and 0.3.
Column B: disease effect estimates based on ZINB models from the ZOE2.0 data in absolute values ($|\delta_\mu|, |\delta_\theta|, |\delta_\pi|$)
Column C: batch effect estimates based on ZINB models from the ZOE2.0 data in absolute values ($|\kappa_\mu|, |\kappa_\theta|, |\kappa_\pi|$). The quartiles of the estimated parameters are provided in Web Table S9.

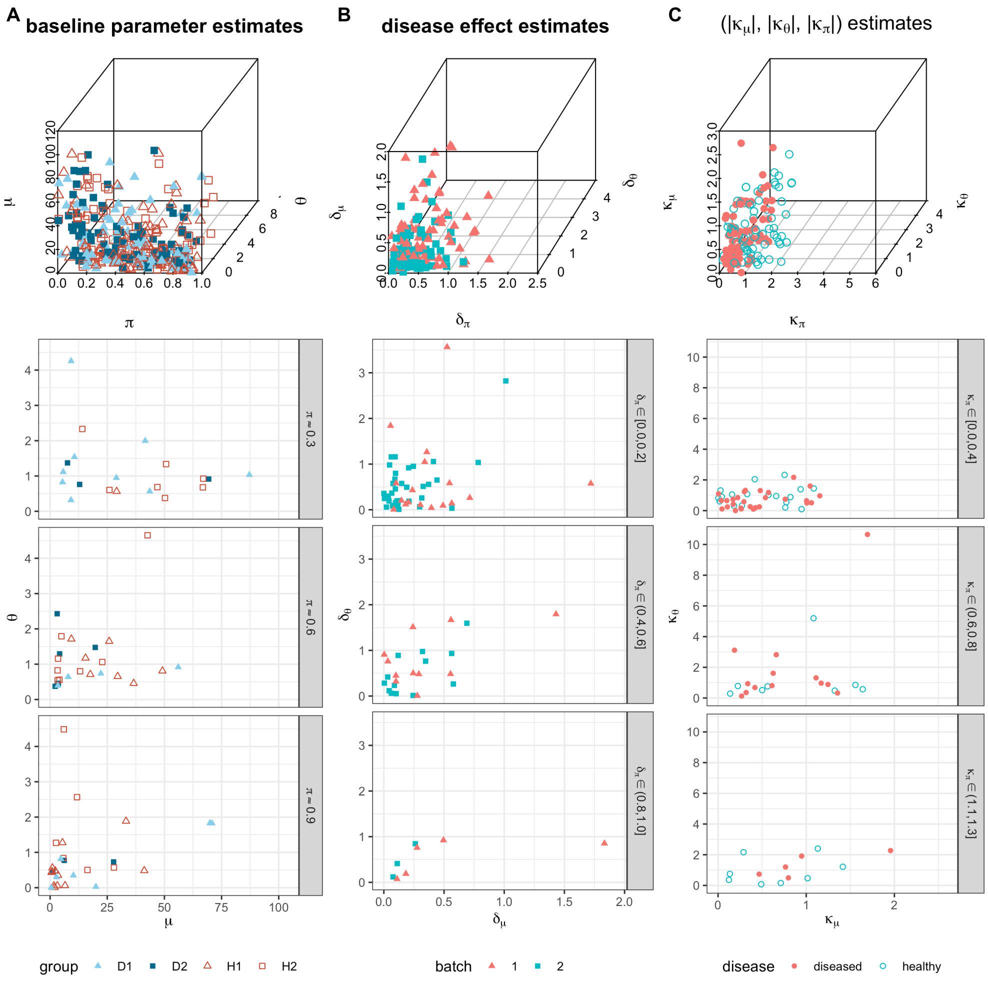

Fig. S9: ZILN parameter estimates for species

Column A: parameter estimates of baseline ZILN distributions in the ZOE2.0 data are presented with a 3-dimensional scatter plot on the top row and each of the subsequent rows represents π estimates being within 0.03 from 0.9, 0.6, and 0.3.

Column B: disease effect estimates based on ZILN models from the ZOE2.0 data in absolute values $(|\delta_\mu|, |\delta_\theta|, |\delta_\pi|)$

Column C: batch effect estimates based on ZILN models from the ZOE2.0 data in absolute values $(|\kappa_\mu|, |\kappa_\theta|, |\kappa_\pi|)$. The quartiles of the estimated parameters are provided in Web Table S10.

parameters	1Q	2Q	3Q
μ	5.80	13.10	39.40
θ	0.50	0.80	1.40
π	0.25	0.53	0.79
δ_μ	0.10	0.20	0.40
δ_θ	0.20	0.50	0.90
δ_π	0.20	0.30	0.50
κ_μ	0.30	0.50	1.00
κ_θ	0.30	0.60	1.00
κ_π	0.30	0.70	1.20

Tab. S10: The ZILN parameter estimate distribution of species in the ZOE2.0 data

parameters	1Q	2Q	3Q
μ	4.40	11.30	30.60
θ	0.80	1.50	2.80
π	0.14	0.38	0.67
δ_μ	0.10	0.20	0.40
δ_θ	0.20	0.40	0.80
δ_π	0.20	0.50	1.10
κ_μ	0.30	0.60	1.00
κ_θ	0.20	0.60	1.00
κ_π	0.50	1.10	2.20

Tab. S11: The ZINB parameter estimate distribution of species in the ZOE2.0 data

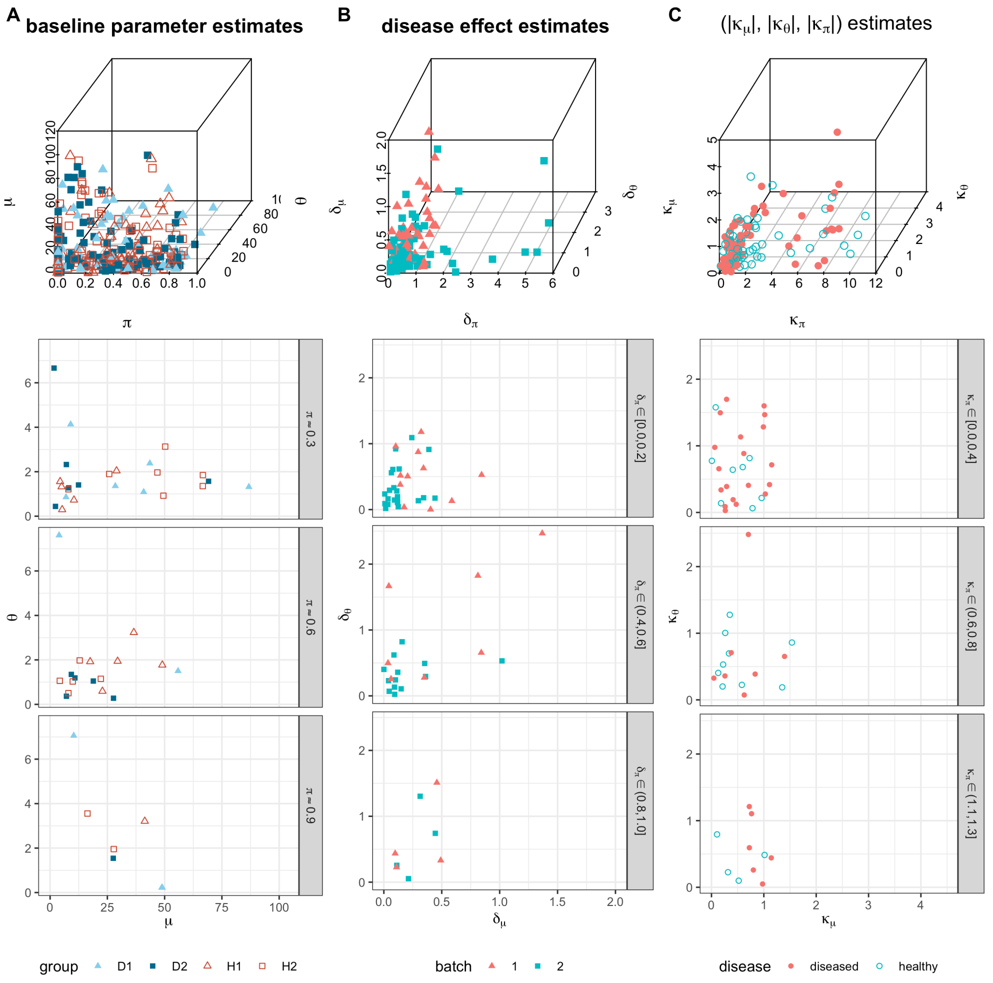

Fig. S10: ZINB parameter estimates for species
Column A: parameter estimates of baseline ZINB distributions in the ZOE2.0 data are presented with a 3-dimensional scatter plot on the top row and each of the subsequent rows represents π estimates being within 0.03 from 0.9, 0.6, and 0.3.
Column B: disease effect estimates based on ZINB models from the ZOE2.0 data in absolute values $(|\delta_\mu|, |\delta_\theta|, |\delta_\pi|)$
Column C: batch effect estimates based on ZINB models from the ZOE2.0 data in absolute values $(|\kappa_\mu|, |\kappa_\theta|, |\kappa_\pi|)$. The quartiles of the estimated parameters are provided in Web Table S11.

S6 Goodness of fit results

S6.1 Goodness of fit for the ZINB models

Web Figure S11 presents the goodness of fit results of the ZINB model for two randomly chosen genes in the ZOE2.0 data. The ZINB distribution provides a decent approximation to the RPK and TPM transformations, although it does not fit well to the arcsin transformed data.

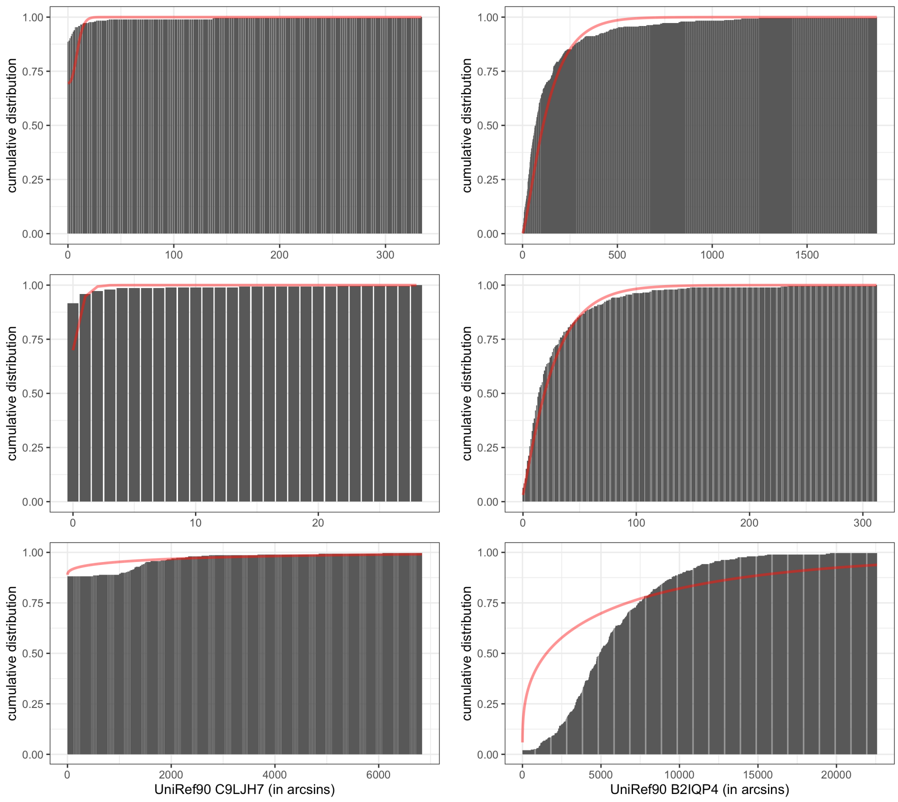

Fig. S11: The empirical distribution (bars) and the estimated ZINB distribution (curves) based on RPK (top), TPM (middle), and arcsin (bottom) transformation of two randomly chosen genes (LEFT: C9LJH7, RIGHT: B2QP4).

S7 Parametric model-based simulation results

S7.1 Full results under ZILN models

These results are presented in the main text.

S7.2 Full results under ZINB models

S7.2.1 Full results under ZINB models—Sensitivity

Results are presented in Figures S12 and S13.

S7.2.2 Type I error and FDR under ZINB model for mean shift (alternative hypothesis D2)

Results are presented in Figure S14.

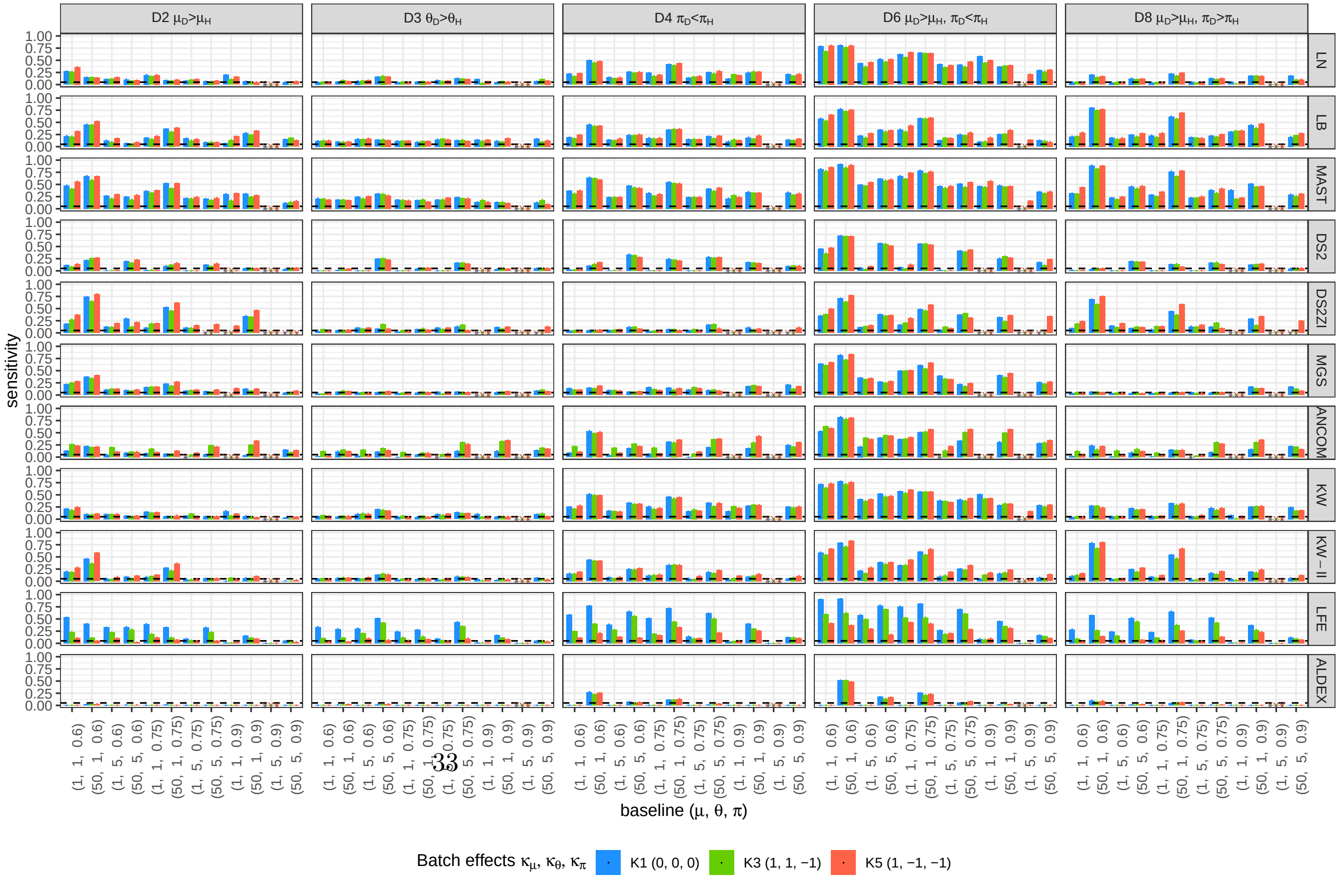

Fig. S12: Full results under ZINB model for a sample size of 80. DS2 = DESeq2, DS2ZI = DESeq2-ZINBWaVE, ANCOM = ANCOM-BC2, LFE = LEfSe, ALDEX = ALDEx2.

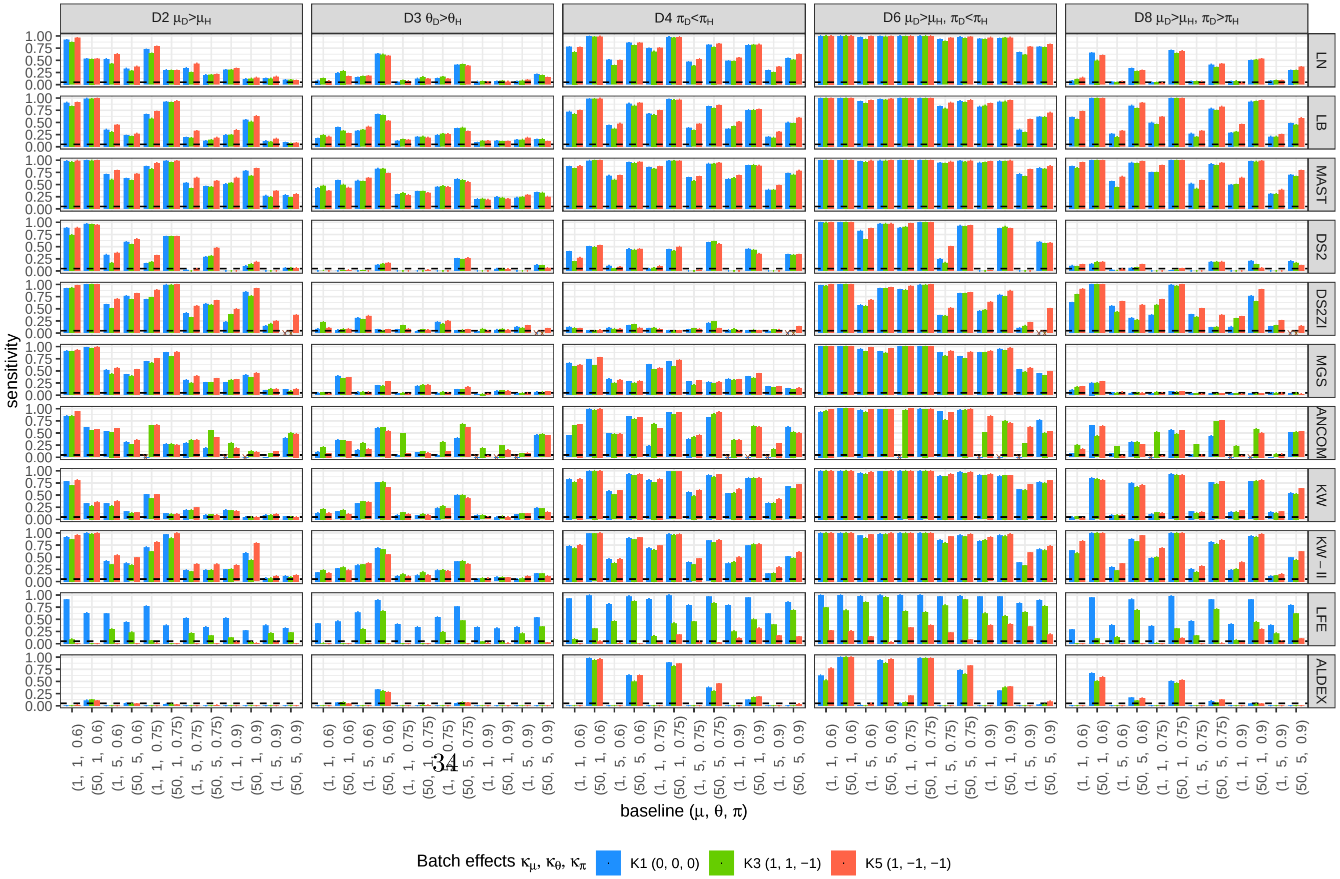

Fig. S13: Full results under ZINB model for a sample size of 400. DS2 = DESeq2, DS2ZI = DESeq2-ZINBWaVE, ANCOM = ANCOM-BC2, LFE = LEfSe, ALDEX = ALDEx2.

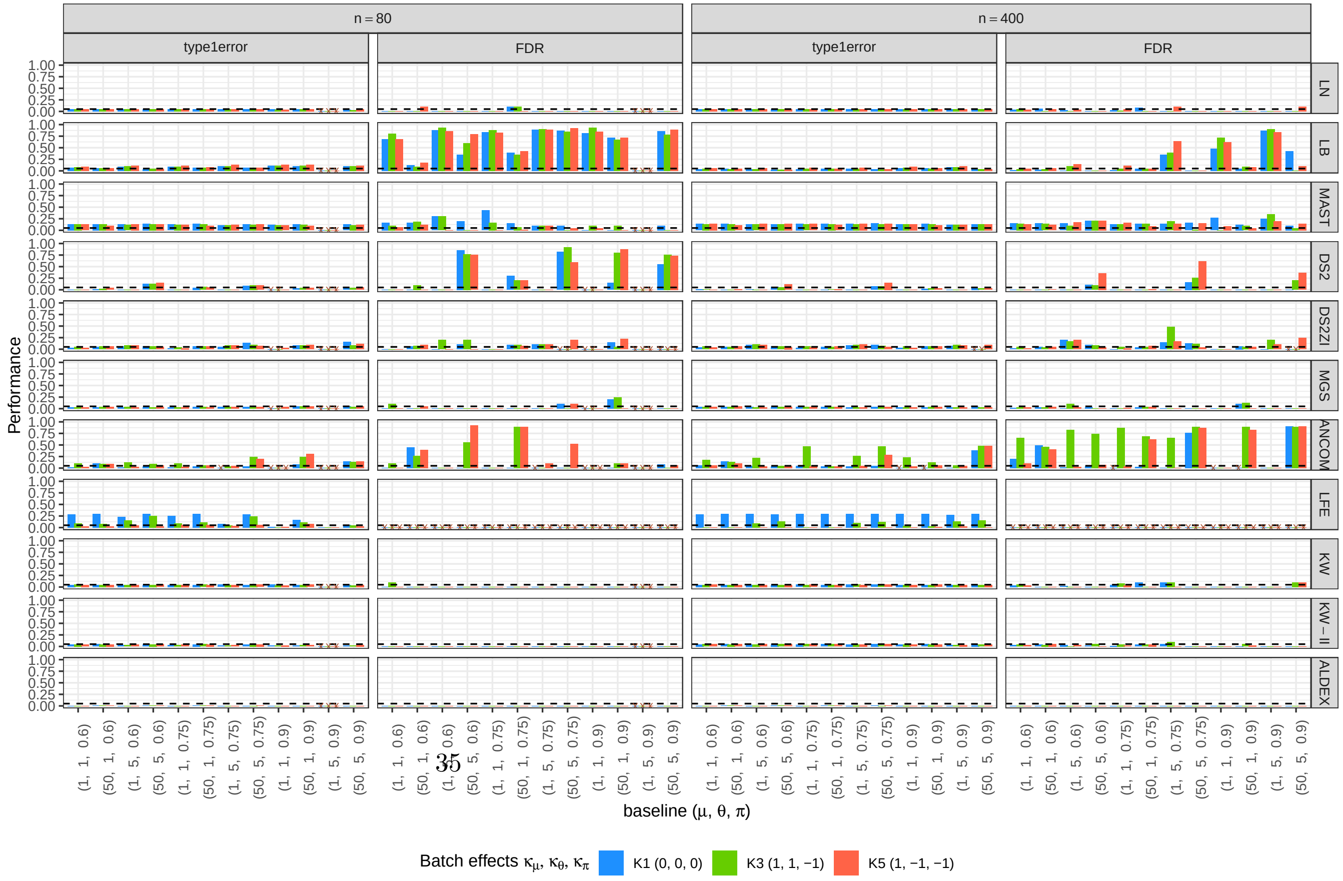

Fig. S14: Type I error and FDR results under ZINB model for mean shift (alternative hypothesis D2). DS2 = DESeq2, DS2ZI = DESeq2-ZINBWaVE, ANCOM = ANCOM-BC2, LFE = LEfSe, ALDEX = ALDEx2.

S7.3 Full results under ZIG models

S7.3.1 Full results under ZIG models—Sensitivity

Results are presented in Figures S15 and S16.

S7.4 Type I error and FDR results under ZIG model for mean shift (alternative hypothesis D2)

Results are presented in Figure S17.

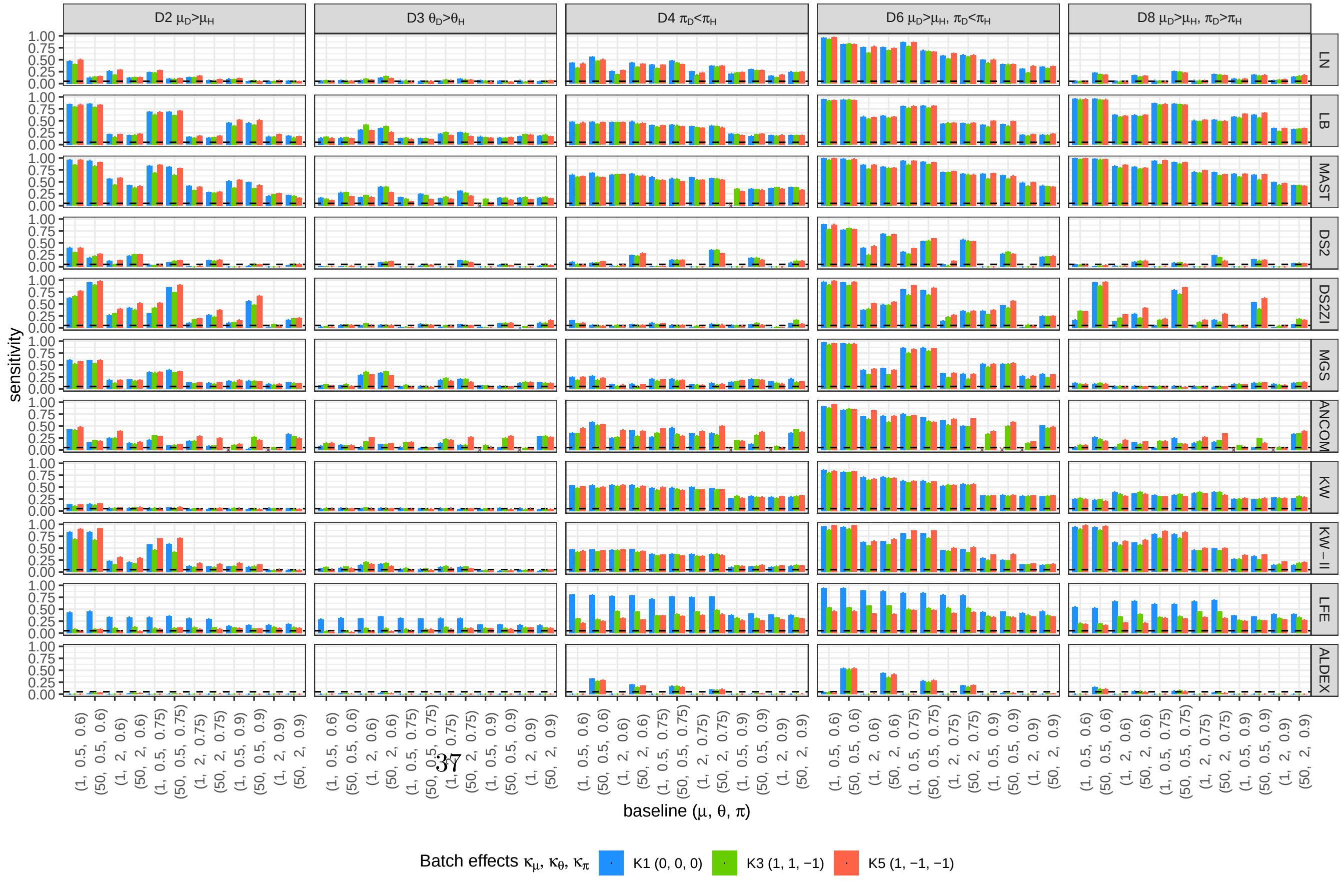

Fig. S15: Full results under ZIG model for a sample size of 80. DS2 = DESeq2, DS2ZI = DESeq2-ZINBWaVE, ANCOM = ANCOM-BC2, LFE = LEfSe, ALDEX = ALDEx2.

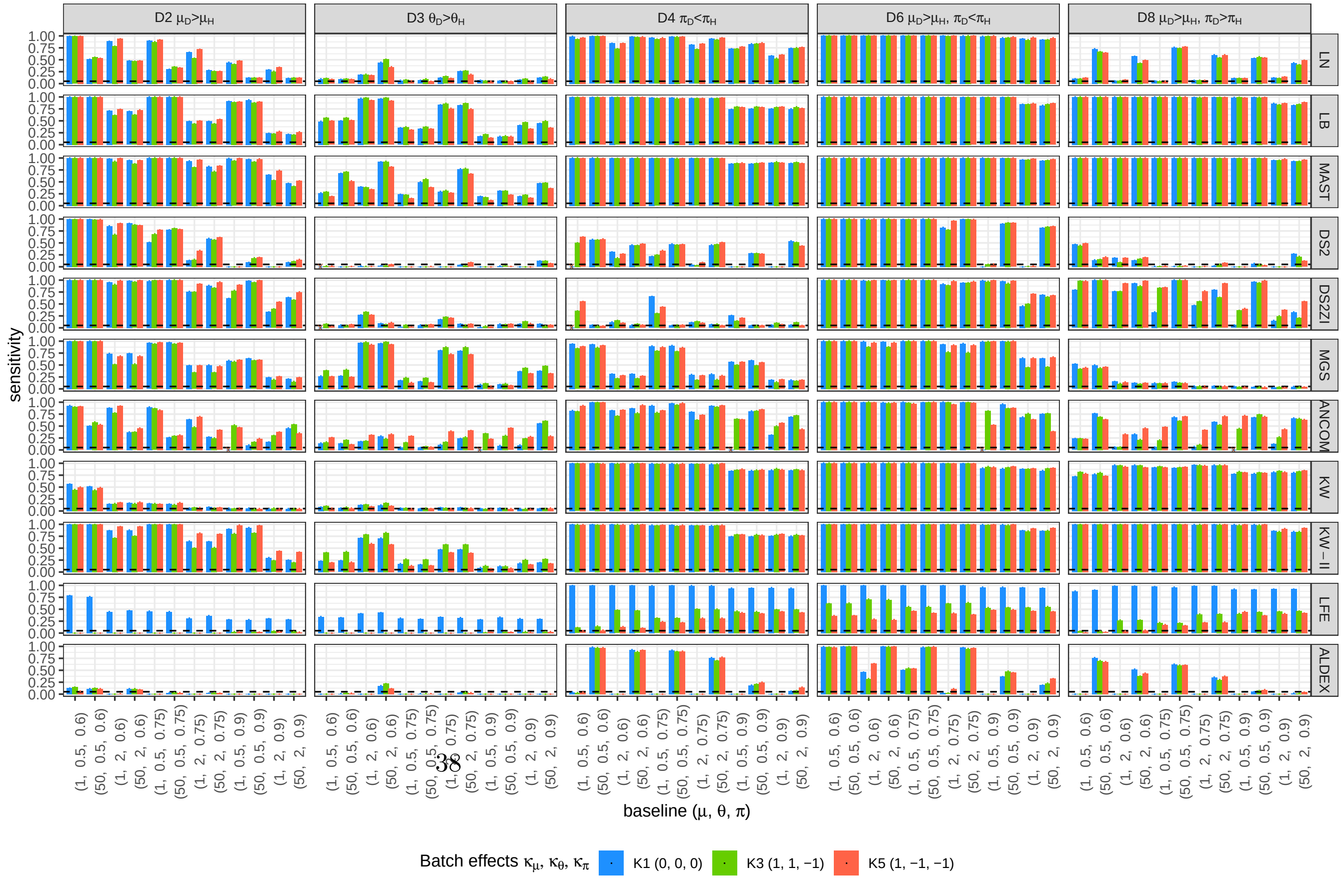

Fig. S16: Full results under ZIG model for a sample size of 400. DS2 = DESeq2, DS2ZI = DESeq2-ZINBWaVE, ANCOM = ANCOM-BC2, LFE = LEfSe, ALDEX = ALDEx2.

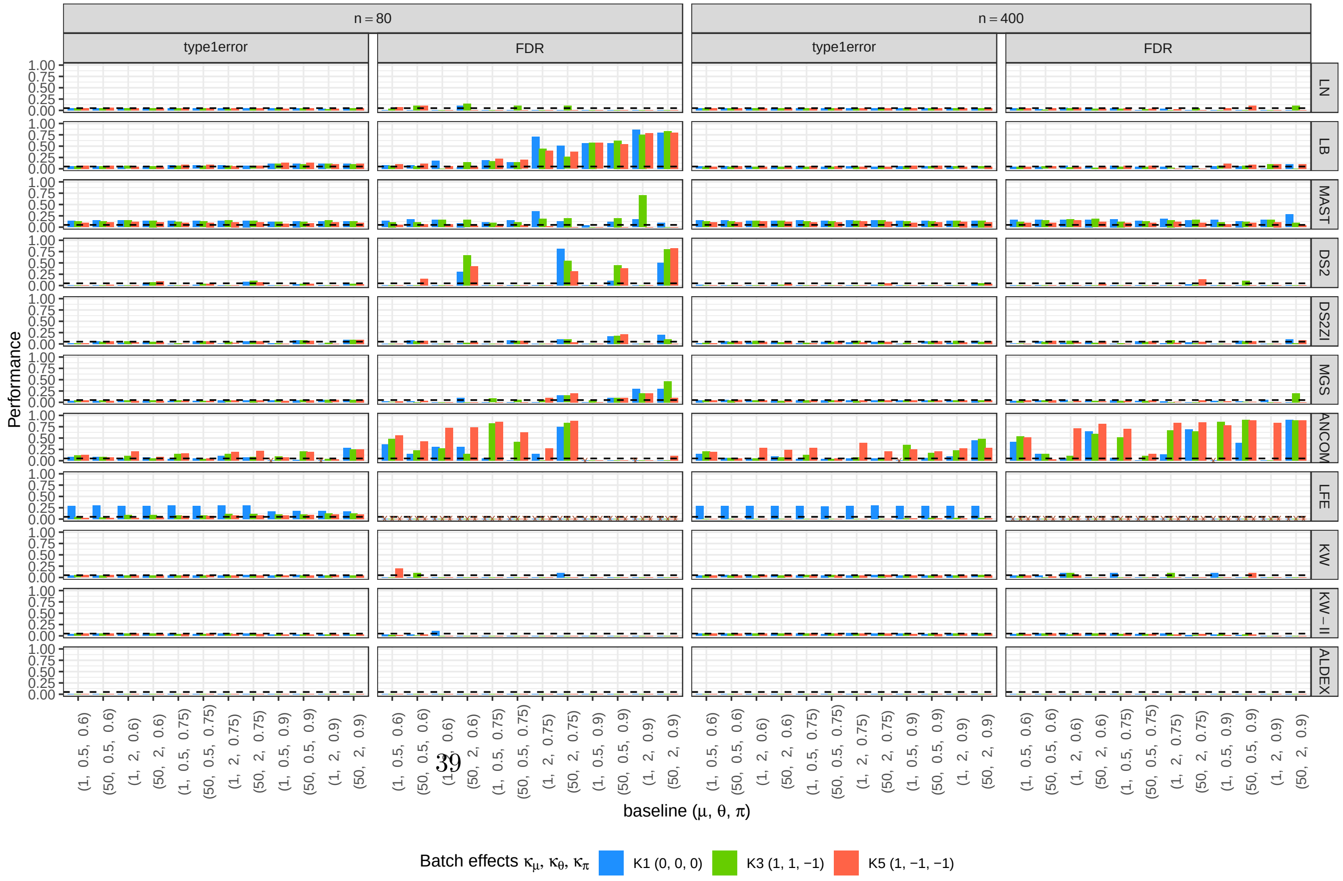

Fig. S17: Type I error and FDR results under ZIG model under for mean shift (alternative hypothesis D2). DS2 = DESeq2, DS2ZI = DESeq2-ZINBWaVE, ANCOM = ANCOM-BC2, LFE = LEfSe, ALDEX = ALDEx2.

S8 Application to the ZOE2.0 data

This section includes the analysis results from the ZOE2.0 data in terms of species and gene-species combinations. The same normalization and screening procedures are applied to these analyses except for the screening thresholds. As for the abundance-based screening, gene-species with TPM less than 0.2 and species with TPM less than 2 were filtered and not tested. Out of 535,299 gene-species combinations, after screening, 188,957 gene-species remained for testing. Out of 209 species, after screening, 97 species remained for testing.

S8.1 Application to the ZOE2.0 data - gene-species

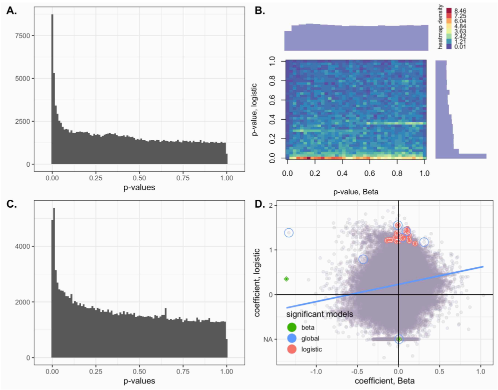

Fig. S18: A. Histogram of p-values obtained from log-normal models (gene-species combinations). B. Histogram of the joint p-values of the logistic Beta models (logistic and Beta parts; gene-species combination). C. Histogram of the single p-values of the logistic Beta models (Wald statistics; gene-species combinations). D. Scatter plot of the coefficients of the LB models (gene-species combinations), with the circled dots representing the most significant gene-species—Wald statistic $p < 10^{-5}$.

The ten most significant gene-species combinations according to the LN model are C8PHV7, C8PEV7, C8PKG9, C8PI10, C8PH26, C8PIH7, C8PHR6, C8PHV8, C8PFD0, and C8PG15, all harbored by *Campylobacter gracilis*.

Using the LB model, there were seven gene-species combinations with global p-value is less than 10^{-5}, including E0DJ07 *Corynebacterium matruchotii*, C8PHV7 *Campylobacter gracilis*, A3CQN5 *Streptococcus cristatus*, C8PEV7 *Campylobacter gracilis*, G1WEB2 *Prevotella oulorum*, C7NCB2 *Leptotrichia shahii*, and C8PHV8 *Campylobacter gracilis*.

The species and proteins mapped to and the functions of those genes can be found in Web Table S12

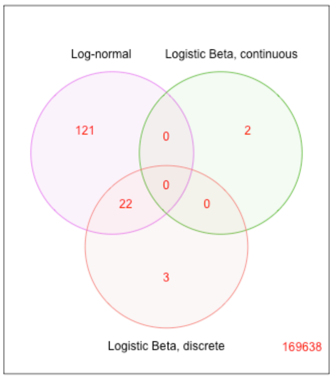

Fig. S19: Venn diagram of gene-species with p-values less than 10^{-5} for each model.

S8.2 Application to the ZOE2.0 data - species

The eight species, of which global p-value is less than 10^{-2} according to the LB model, are *Campylobacter gracilis*, *Streptococcus cristatus*, *Leptotrichia hofstadii*, *Lachnoanaerobaculum saburreum*, *Leptotrichia shahii*, *Streptococcus mutans*, *Campylobacter concisus*, and *Prevotella oulorum*.

S8.3 Application to the ZOE2.0 data - gene profiles

Tab. S12: Profiles of the top significant genes and gene-species based on the UniProt database [30]. * genes from the list of top significant genes, † genes from the list of top significant gene-species.

gene ID<br>test reference	gene name<br>protein<br>function	organism
A3CQN5	rsmA	Streptococcus sanguinis (strain SK36)
†	Ribosomal RNA small subunit methyltransferase A.<br>Specifically dimethylates two adjacent adenosines (A1518 and A1519) in the loop of a conserved hairpin near the 3'-end of 16S rRNA in the 30S particle. May play a critical role in biogenesis of 30S subunits.	
C7NCB2	Lebu_0017	Leptotrichia buccalis (strain ATCC 14201 / DSM 1135 / JCM 12969 / NCTC 10249 / C-1013-b)
†	MATE efflux family protein.	

	GO - Molecular function. antiporter activity, xenobiotic transmembrane transporter activity.	
C8PEV7 *†	CAMGR0001_2596 Uncharacterized protein. -	Campylobacter gracilis RM3268
C8PFD0 †	CAMGR0001_2483 Uncharacterized protein. -	Campylobacter gracilis RM3268
C8PG15 †	CAMGR0001_0808 Transcriptional regulator, LysR family. -	Campylobacter gracilis RM3268
C8PG93 *	CAMGR0001_0886 Tat pathway signal sequence domain protein. -	Campylobacter gracilis RM3268
C8PH26 *†	CAMGR0001_2214 Uncharacterized protein. -	Campylobacter gracilis RM3268
C8PHR6 †	CAMGR0001_0512 Methylenetetrahydrofolate reductase. -	Campylobacter gracilis RM3268
C8PHV7 *†	CAMGR0001_0553 Uncharacterized protein. -	Campylobacter gracilis RM3268
C8PHV8 *†	CAMGR0001_0554 Uncharacterized protein. -	Campylobacter gracilis RM3268
C8PI10 *†	serS Serine–tRNA ligase. Catalyzes the attachment of serine to tRNA(Ser). Is also able to aminoacylate tRNA(Sec) with serine, to form the misacylated tRNA L-seryl-tRNA(Sec), which will be further converted into selenocysteinyl-tRNA(Sec).	Campylobacter gracilis RM3268
C8PIH7 *†	accD Acetyl-coenzyme A carboxylase carboxyl transferase subunit beta. Component of the acetyl coenzyme A carboxylase (ACC) complex. Biotin carboxylase (BC) catalyzes the carboxylation of biotin on its carrier protein (BCCP) and then the CO_2 group is transferred by the transcarboxylase to acetyl-CoA to form malonyl-CoA.	Campylobacter gracilis RM3268
C8PKG9 *†	CAMGR0001_0190 NFACT-R_1 domain-containing protein. -	Campylobacter gracilis RM3268
C8PKZ2 *	asd Aspartate-semialdehyde dehydrogenase.	Campylobacter gracilis RM3268

	Catalyzes the NADPH-dependent formation of L-aspartate-semialdehyde (L-ASA) by the reductive dephosphorylation of L-aspartyl-4-phosphate.	
C8PJD1<br>*	ilvC<br>Ketol-acid reductoisomerase (NADP(+)).<br>Involved in the biosynthesis of branched-chain amino acids (BCAA). Catalyzes an alkyl-migration followed by a ketol-acid reduction of (S)-2-acetolactate (S2AL) to yield (R)-2,3-dihydroxy-isovalerate. In the isomerase reaction, S2AL is rearranged via a Mg-dependent methyl migration to produce 3-hydroxy-3-methyl-2-ketobutyrate (HMKB). In the reductase reaction, this 2-ketoacid undergoes a metal-dependent reduction by NADPH to yield (R)-2,3-dihydroxy-isovalerate.	Campylobacter gracilis RM3268
C8PJY1<br>*	pyrH<br>Uridylate kinase.<br>Catalyzes the reversible phosphorylation of UMP to UDP.	Campylobacter gracilis RM3268
E0DI62<br>*	HMPREF0299 5372<br>Sua5/YciO/YrdC/YwlC family protein<br>-	Corynebacterium matruchotii ATCC 14266
E0DJ07<br>†	HMPREF0299 5672<br>Phosphoserine transaminase<br>-	Corynebacterium matruchotii ATCC 14266
G1WEB2<br>†	HMPREF9431_02084<br>Uncharacterized protein.<br>-	Prevotella oulorum F0390

S8.4 Summary for the ZOE2.0 results

The ten genes with the lowest p-values from the LN models are: C8PIH7, C8PI10, C8PHV7, C8PEV7, C8PKZ2, C8PJY1, C8PG93, C8PKG9, C8PH26, and C8PJD1. The globally significant DE genes according to the LB Wald test were E0DI62, C8PHV7, C8PEV7, C8PI10, C8PIH7, and C8PHV8. The species and proteins associated with those genes and their functions listed in UniProt are presented in Web Table S12 of the Supplement. Similar results are obtained for the gene-species level of analysis (Web section S8); the histogram has a hike at the low p-value areas for the LN and global LB tests, significance mostly comes from the discrete part than the continuous part, and the directions of the two parts in the significant taxonomic units are weakly consistent with each other. A3CQN5, harbored by *Streptococcus sanguinis*, was the only gene identified in the gene-species level analysis, suggesting that gene-species data may carry some extra information compared to the marginal gene-family DE analysis. *Corynebacterium matruchotii*, *Campylobacter gracilis*, *Leptotrichia buccalis* and *Prevotella oulorum*, expressing these significant genes were confirmed as significant in the DE analysis of the marginal species data (WebSection S8).

The species most strongly associated with childhood dental caries in this analysis was *Campylobacter gracilis*, a gram-negative anaerobic bacillus, traditionally isolated

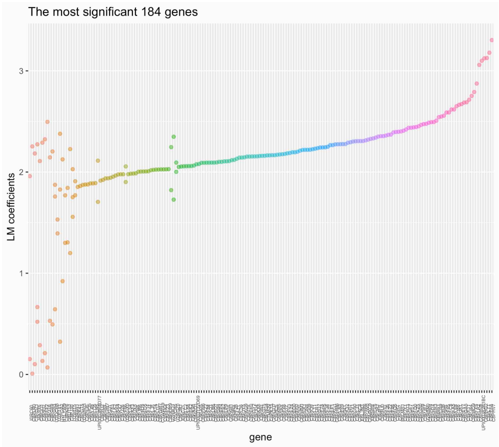

Fig. S20: LN model coefficients of gene-species for each of the most significant genes

from gingival crevices and dental biofilms accumulated close to the gingival margin [31]. Oral campylobacters are enriched in genes encoding for lactate metabolism, which plays an important role in the development and maintenance of acidic conditions in cariogenic biofilms as the predominant glucose-derived product, which is considered to be the main acid involved in dental caries formation [32]. The capacity of *Campylobacter* species to produce lactate may be contributing to the development and establishment of ECC, as other microorganisms directly associated to caries disease like *Streptococcus* sp and *Leptotrichia* sp, benefit from this lactate-rich environment [32, 33]. Chalmers et al. (2015) showed that *Campylobacters gracilis* is associated with severe ECC at a frequency detection rate of 87.5% [34]. *Campylobacters gracilis*' active genes shown significant associated with ECC in the essential steps for: (1) bacterial growth (C8PIH7, encodes for an enzyme that catalyzes the first committed step in fatty acid synthesis) [35]; (2) protein biosynthesis and transport (C8PI10, encodes for an enzyme that catalyzes the attachment of serine to its cognate transfer RNA molecule; C8PKZ2, encodes for the enzyme from biosynthesis of diverse amino acids leading to L-lysine, L-threonine, L-methionine and L-isoleucine; C8PJD1, encodes for an amino acid biosynthesis pathway) [36, 37]; (3) protein transport (C8PG93 encodes for twin-arginine translocation (Tat) pathway, which catalyzes the export of proteins from the cytoplasm across the inner/cytoplasmic membrane.) [38]; (4) DNA replication and transcription (C8PJY1, encodes for key enzymes in the synthesis of nucleoside triphosphates molecular precursors of both DNA and RNA) [39]; (5) biofilm formation or adhesion through gene C8PKG9 (encodes for NFACT-R 1 domain-containing protein) [40] and; (6) energy conservation (C8PHR6 encodes for methylenetetrahydrofolate reductase (MTHFR) of acetogenic bacteria during reduction of carbon dioxide with molecular hydrogen to acetate) [41]. Other genes associated with ECC were A3CQN5 (encodes for Ribosomal RNA small subunit methyltransferase A, which plays the role of switch protein in the ribosome assembly in *Streptococcus sanguinis*) and C7NCB2,

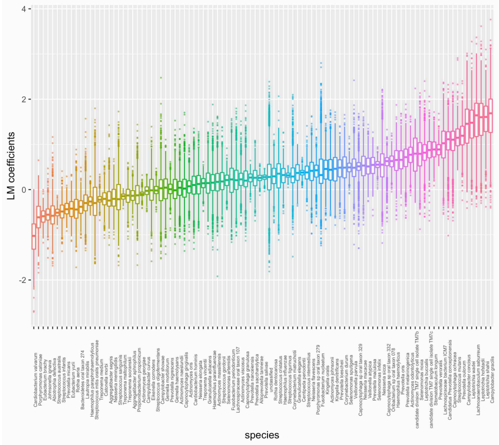

Fig. S21: LN model coefficients of gene-species for each species

which encodes for a multidrug and toxic compound extrusion (MATE) family of efflux pumps to actively transport of a solute across the membrane in *Leptotrichia buccalis* [42].

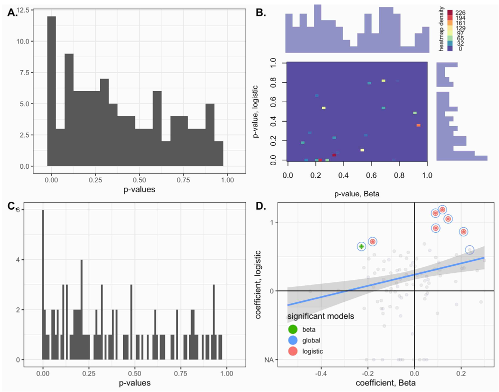

Fig. S22: A. Histogram of the p-values of the log-normal models (for species). B. Histogram of the joint p-values of the logistic Beta models (logistic and Beta parts; for species). C. Histogram of the single p-values of the logistic Beta models (Wald statistics; for species). D. Scatter plot of the coefficients of the LB models (for species), with the circled dots representing the most significant species—Wald statistic $p < 0.01$.

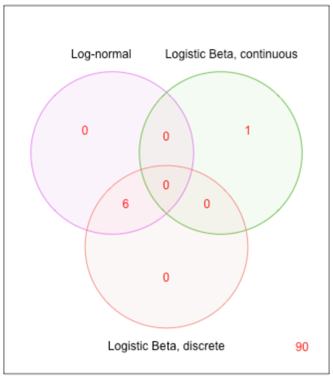

Fig. S23: Venn diagram of species with p-values less than 0.01 for each model.

S9 Application to the IBD data

S9.1 Application to the IBD data - gene profiles

Tab. S13: Profiles of the top significant genes based on the UniProt database [30]. * genes from the list of top significant genes.

gene ID<br>test reference	gene name<br>protein<br>function	organism
R5PRG3<br>*	BN489_01283<br>PlsC domain-containing protein<br>acyltransferase activity	Sutterella wadsworthensis CAG:135
R5QAG2<br>*	BN489_02406<br>Polyamine ABC transporter<br>ATP binding	Sutterella wadsworthensis CAG:135
R5QE55<br>*	BN489_00474<br>Leucine efflux protein<br>amino acid transport	Sutterella wadsworthensis CAG:135
R5PLJ0<br>*	BN489_01810<br>HTH gntR-type domain-containing protein<br>DNA binding	Sutterella wadsworthensis CAG:135
R5Q3H7<br>*	BN489_01382<br>Uncharacterized protein<br>-	Sutterella wadsworthensis
R5Q1H1<br>*	BN489_00687<br>Uncharacterized protein<br>-	Sutterella wadsworthensis CAG:135
S3CE88<br>*	HMPREF1476_01419<br>Uncharacterized protein<br>transmembrane transport	Sutterella wadsworthensis HGA0223
R5QEQ4<br>*	BN489_00642<br>Uncharacterized protein<br>-	Sutterella wadsworthensis CAG:135
G2T243<br>*	RHOM_03530<br>Stage 0 sporulation protein A homolog<br>May play the central regulatory role in sporulation. It may be an element of the effector pathway responsible for the activation of sporulation genes in response to nutritional stress. Spo0A may act in concert with spo0H (a sigma factor) to control the expression of some genes that are critical to the sporulation process.	Roseburia hominis
S3BF40<br>*	HMPREF1476_00737<br>CN hydrolase domain-containing protein<br>nitrogen compound metabolic process	Sutterella wadsworthensis HGA0223

R5Q7C5<br>*	BN489_02016<br>Uroporphyrin-III C/tetrapyrrole methyltransferase<br>methyltransferase activity	Sutterella wadsworthensis CAG:135
R5PTS5<br>*	BN489_00648<br>Uncharacterized protein<br>-	Sutterella wadsworthensis CAG:135
D4IJ04<br>*	AL1_02120<br>Uncharacterized protein<br>-	Alistipes shahii WAL 8301
R5PRP2<br>*	BN489_00146<br>Acetylglutamate kinase<br>ATP binding, kinase activity, cellular amino acid biosynthetic process	Sutterella wadsworthensis CAG:135
R5PJG3<br>*	BN489_00982<br>Uncharacterized protein<br>-	Sutterella wadsworthensis CAG:135
R5PM43<br>*	BN489_01889<br>Uncharacterized protein<br>-	Sutterella wadsworthensis CAG:135
R5W0F1 (R5W0F1_9BACT)<br>*	(Obsolete)<br>(Obsolete)<br>-	Alistipes sp. CAG:53
R5PWS5<br>*	BN489_02130<br>Uncharacterized protein<br>amino acid transport	Sutterella wadsworthensis
R5PNF6<br>*	BN489_02005<br>Uncharacterized protein<br>sulfurtransferase activity	Sutterella wadsworthensis
D4WIY6<br>*	rplC<br>50S ribosomal protein L3<br>rRNA binding, structural constituent of ribosome, translation	Bacteroides ovatus SD CMC 3f
Q0TKG5<br>*	aes<br>Acetyl esterase<br>Displays esterase activity towards short chain fatty esters (acyl chain length of up to 8 carbons). Able to hydrolyze triacetylglycerol (triacetin) and tributyrylglycerol (tributyrin), but not trioleylglycerol (triolein) or cholesterol oleate. Negatively regulates MalT activity by antagonizing maltotriose binding. Inhibits MelA galactosidase activity.	Escherichia coli O6:K15:H31
D1PDG3<br>*	PREVCOP_05253<br>ISPg3, transposase<br>-	Prevotella copri DSM 18205
Q17UW4<br>*	COII<br>Cytochrome c oxidase subunit 2 (Fragment)	Zygosaccharomyces rouxii (Candida mogii)

	Component of the cytochrome c oxidase, the last enzyme in the mitochondrial electron transport chain which drives oxidative phosphorylation. The respiratory chain contains 3 multisubunit complexes succinate dehydrogenase (complex II, CII), ubiquinol-cytochrome c oxidoreductase (cytochrome b-c1 complex, complex III, CIII) and cytochrome c oxidase (complex IV, CIV), that cooperate to transfer electrons derived from NADH and succinate to molecular oxygen, creating an electrochemical gradient over the inner membrane that drives transmembrane transport and the ATP synthase. Cytochrome c oxidase is the component of the respiratory chain that catalyzes the reduction of oxygen to water. Electrons originating from reduced cytochrome c in the intermembrane space (IMS) are transferred via the dinuclear copper A center (CU(A)) of subunit 2 and heme A of subunit 1 to the active site in subunit 1, a binuclear center (BNC) formed by heme A3 and copper B (CU(B)). The BNC reduces molecular oxygen to 2 water molecules using 4 electrons from cytochrome c in the IMS and 4 protons from the mitochondrial matrix.	
E2ZM16<br>*	HMPREF9436_02727<br>DUF3887 domain-containing protein<br>Enzyme and pathway databases: FCF748224-HMP:GTSS-1911-MONOMER	Faecalibacterium cf. prausnitzii KLE1255
R7NP61 (R7NP61_9BACE)<br>*	(Obsolete)<br>(Obsolete) iron-regulated protein A<br>-	Bacteroides sp. CAG:98
U2ZZD9 (U2ZZD9_VIBAL)<br>*	(Obsolete)<br>(Obsolete) partial hypothetical protein<br>-	Vibrio alginolyticus
R6W6W2<br>*	BN607_03102<br>Uncharacterized protein<br>-	Bacteroides faecis CAG:32
I9USK4<br>*	HMPREF1074_02638<br>Uncharacterized protein<br>-	Bacteroides xylanisolvens CL03T12C04
B0NN15<br>*	BACSTE_00845<br>Uncharacterized protein<br>-	Bacteroides stercoris ATCC 43183

S9.2 Application to the IBD data - alternative tests

Alternative tests to the logistic regression of the LB test were implemented using the likelihood-ratio and Fisher's exact tests. The likelihood-ratio test statistic is obtained by twice the difference in the log-likelihoods of the full model and the reduced model. The full model is the same as specified in the main body of paper (Section 6), and the reduced model simply lacks the disease feature from the full model. The p-value is obtained from the reference distribution of χ^2_1. The Fisher's exact test may not be

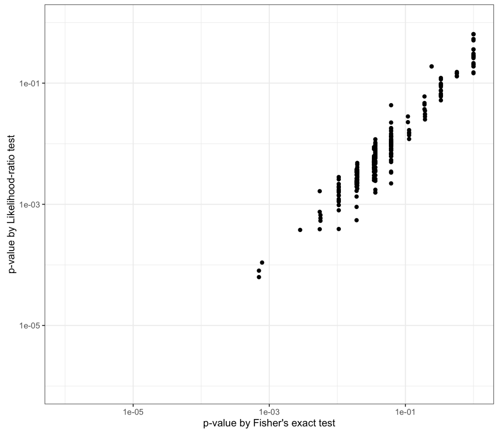

Fig. S24: P-values of the likelihood-ratio and Fisher's exact tests for genes with one of the subgroup prevalence rate at the boundary in the IBD data

as powerful as the likelihood-ratio test in this context because it can only be applied in the case of a two-by-two table, thus not being able to account for any covariates. Figure S24 confirms that the p-values from the Exact test are overall larger than those of the likelihood-ratio test. None of the two tests identified any genes at the $p < 10^{-5}$ significance level.

S9.3 Summary for the IBD results

The 10 most statistically significant genes in the LN model are S3BI82, R5Q3H7, R5PRG3, R5Q1H1, R5QAG2, R5QE55, S3CE88, R5QEQ4, R5PLJ0, and G2T243 while the top ten genes for the LB model are D4WIY6, Q0TKG5, R6W6W2, D1PDG3, Q17UW4, I9USK4, E2ZM16, R7NP61, B0NN15, and U2ZZD9 with species and proteins associated with those genes and their functions presented in Web Table S13 in Section S9. Most of the top 20 genes in LN tests corresponded to *Sutterella wadswothensis*, a Gram-negative, non-spore-forming rod of the Betaproteobacteria class that grows in a microaerophilic atmosphere or under anaerobic conditions, and was previously identified as significantly differentially abundant (prior to the correction for multiple hypothesis testing) in the original IBD study report [25]. The role of *S. wadworthensis* in IBD pathogenesis remains unresolved with recent studies supporting [43, 44] and some earlier studies not supporting this association [45, 46]. The most significant gene family S3BI82, corresponded to the hydrogenase maturation factor HypA. Hydrogenases are important factors that contribute to the metabolic versatility of Enterobacteriaceae, specifically their ability to utilize a large repertoire of terminal electron acceptors, which allows them to thrive in the inflamed gut [47, 48, 49]. Gene G2T243 is a stage 0 sporulation protein A homolog harbored by *Roseburia hominis*. *R. hominis*, a prevalent butyrate producer, has been found consistently depleted in CD ([50, 51] and was also identified as significant prior to the multiple testing correction

in the original IRB study [25]). Alistipes, the other other genus found in top list, is member of the Bacteroidetes phylum. *A. finegoldii* is considered a protective species against colitis

The 10 significant gene families identified in LB are quite different to what are in LN, and correspond to *Bacteroides ovatus, Escherichia coli, Prevotella copri, Faecalibacterium prausnitzii, Bacteroides faecis, Bacteroides xylanisolvens*, and *Bacteroides stercoris*; all identified as significant in the original study [25] prior to multiple hypothesis correction. Although the species *Zygosaccharomyces rouxii* harboring Q17UW4 was not reported in the original study [25], the gene family itself corresponded to a cytochrome c oxidase subunit 2, which is increased in UC [52]. Finally, the relevance of *Faecalibacterium praunitzii* as an important beneficial bacterium in IBD has been demonstrated in a number of studies [53]; alongside the butyrate-producing *R. hominis*, they were identified in the original IBD study report [25] and account for some of the strongest identified associations.

References

[1] Xiaoling Peng, Gang Li, and Zhenqiu Liu. Zero-inflated beta regression for differential abundance analysis with metagenomics data. *Journal of Computational Biology*, 23(2):102–110, 2016.

[2] D Mikis Stasinopoulos and Robert A Rigby. Generalized additive models for location scale and shape (gamlss) in r. *Journal of Statistical Software*, 23(7):1–46, 2007.

[3] Greg Finak, Andrew McDavid, Masanao Yajima, Jingyuan Deng, Vivian Gersuk, Alex K Shalek, Chloe K Slichter, Hannah W Miller, M Juliana McElrath, Martin Prlic, et al. Mast: a flexible statistical framework for assessing transcriptional changes and characterizing heterogeneity in single-cell rna sequencing data. *Genome biology*, 16(1):278, 2015.

[4] Andrew McDavid, Greg Finak, and Masanao Yajima. *MAST: Model-based Analysis of Single Cell Transcriptomics*, 2019. R package version 1.8.2.

[5] Michael I Love, Wolfgang Huber, and Simon Anders. Moderated estimation of fold change and dispersion for rna-seq data with deseq2. *Genome biology*, 15(12):550, 2014.

[6] Simon Anders and Wolfgang Huber. Differential expression analysis for sequence count data. *Nature Precedings*, pages 1–1, 2010.

[7] Koen Van den Berge, Fanny Perraudeau, Charlotte Soneson, Michael I Love, Davide Risso, Jean-Philippe Vert, Mark D Robinson, Sandrine Dudoit, and Lieven Clement. Observation weights unlock bulk rna-seq tools for zero inflation and single-cell applications. *Genome biology*, 19(1):24, 2018.

[8] Joseph N Paulson, O Colin Stine, Héctor Corrada Bravo, and Mihai Pop. Differential abundance analysis for microbial marker-gene surveys. *Nature methods*, 10(12):1200, 2013.

[9] Joseph Nathaniel Paulson. *Normalization and differential abundance analysis of metagenomic biomarker-gene surveys*. PhD thesis, University of Maryland, College Park, 2015.

[10] Joseph N Paulson, Nathan D. Olson, Domenick J. Braccia, Justin Wagner, Hisham Talukder, Mihai Pop, and Hector Corrada Bravo. metagenomeseq: Statistical analysis for sparse high-throughput sequncing, 2013. http://www.cbcb.umd.edu/software/metagenomeSeq.

[11] Siddhartha Mandal, Will Van Treuren, Richard A White, Merete Eggesbø, Rob Knight, and Shyamal D Peddada. Analysis of composition of microbiomes: a novel method for studying microbial composition. *Microbial ecology in health and disease*, 26(1):27663, 2015.

[12] Huang Lin and Shyamal Das Peddada. Analysis of compositions of microbiomes with bias correction. *Nature communications*, 11(1):1–11, 2020.

[13] Nicola Segata, Jacques Izard, Levi Waldron, Dirk Gevers, Larisa Miropolsky, Wendy S Garrett, and Curtis Huttenhower. Metagenomic biomarker discovery and explanation. *Genome biology*, 12(6):1–18, 2011.

[14] Andrew D Fernandes, Jennifer Ns Reid, Jean M Macklaim, Thomas A McMurrough, David R Edgell, and Gregory B Gloor. Unifying the analysis of high-throughput sequencing datasets: characterizing rna-seq, 16s rrna gene sequencing and selective growth experiments by compositional data analysis. *Microbiome*, 2(1):1–13, 2014.

[15] William H Kruskal and W Allen Wallis. Use of ranks in one-criterion variance analysis. *Journal of the American statistical Association*, 47(260):583–621, 1952.

[16] Torsten Hothorn, Kurt Hornik, Mark A Van De Wiel, and Achim Zeileis. A lego system for conditional inference. *The American Statistician*, 60(3):257–263, 2006.

[17] Torsten Hothorn. Package 'coin'. 2019.

[18] Peter A Lachenbruch. Analysis of data with clumping at zero. *Biometrische Zeitschrift*, 18(5):351–356, 1976.

[19] Sandra Taylor and Katherine Pollard. Hypothesis tests for point-mass mixture data with application to omics data with many zero values. *Statistical Applications in Genetics and Molecular Biology*, 8(1):1–43, 2009.

[20] Brandie D Wagner, Charles E Robertson, and J Kirk Harris. Application of two-part statistics for comparison of sequence variant counts. *PloS one*, 6(5):e20296, 2011.

[21] Nigel B Pitts, Ramon J Baez, Carolina Diaz-Guillory, Kevin J Donly, Carlos Alberto Feldens, Colman McGrath, Prathip Phantumvanit, W Kim Seow, Nikolai Sharkov, Yupin Songpaisan, et al. Early childhood caries: Iapd bangkok declaration. *Journal of dentistry for children (Chicago, Ill.)*, 86(2):72, 2019.

[22] Kimon Divaris, Gary D. Slade, Andrea G. Ferreira Zandona, John S. Preisser, Jeannie Ginnis, Miguel A. Simancas-Pallares, Cary S. Agler, Poojan Shrestha, Deepti S. Karhade, Apoena de Aguiar Ribeiro, Hunyong Cho, B. Yu Gu, Beau D. Meyer, Ashwini R. Joshi, M. Andrea Azcarate-Peril, Patria V. Basta, Di Wu, and Kari E. North. Cohort profile: Zoe 2.0—a community-based, genetic epidemiologic study of early childhood oral health. *International Journal of Environmental Research and Public Health*, 17(21):8056, 2020.

[23] Kimon Divaris, Dmitry Shungin, Adaris Rodríguez-Cortés, Patricia V Basta, Jeff Roach, Hunyong Cho, Di Wu, Andrea G Ferreira Zandoná, Jeannie Ginnis, Sivapriya Ramamoorthy, et al. The supragingival biofilm in early childhood caries: clinical and laboratory protocols and bioinformatics pipelines supporting oral metagenomics, metatranscriptomics and metabolomics studies of the oral microbiome. *Methods in molecular biology (Clifton, NJ)*, 1922:525, 2019.

[24] Sahar Abubucker, Nicola Segata, Johannes Goll, Alyxandria M Schubert, Jacques Izard, Brandi L Cantarel, Beltran Rodriguez-Mueller, Jeremy Zucker, Mathangi Thiagarajan, Bernard Henrissat, et al. Metabolic reconstruction for metagenomic data and its application to the human microbiome. *PLoS Comput Biol*, 8(6):e1002358, 2012.

[25] Jason Lloyd-Price, Cesar Arze, Ashwin N Ananthakrishnan, Melanie Schirmer, Julian Avila-Pacheco, Tiffany W Poon, Elizabeth Andrews, Nadim J Ajami, Kevin S Bonham, Colin J Brislawn, et al. Multi-omics of the gut microbial ecosystem in inflammatory bowel diseases. *Nature*, 569(7758):655–662, 2019.

[26] John S Preisser, John W Stamm, D Leann Long, and Megan E Kincade. Review and recommendations for zero-inflated count regression modeling of dental caries indices in epidemiological studies. *Caries research*, 46(4):413–423, 2012.

[27] Davide Risso, Fanny Perraudeau, Svetlana Gribkova, Sandrine Dudoit, and Jean-Philippe Vert. A general and flexible method for signal extraction from single-cell rna-seq data. *Nature communications*, 9(1):284, 2018.

[28] Jun Chen, Emily King, Rebecca Deek, Zhi Wei, Yue Yu, Diane Grill, and Karla Ballman. An omnibus test for differential distribution analysis of microbiome sequencing data. *Bioinformatics*, 34(4):643–651, 2017.

[29] Yinglin Xia, Jun Sun, and Ding-Geng Chen. *Statistical analysis of microbiome data with R*. Springer, 2018.

[30] UniProt Consortium. Uniprot: a worldwide hub of protein knowledge. *Nucleic acids research*, 47(D1):D506–D515, 2019.

[31] Peter Vandamme, MI Daneshvar, FE Dewhirst, BJ Paster, Karel Kersters, H Goossens, and CW Moss. Chemotaxonomic analyses of bacteroides gracilis and bacteroides ureolyticus and reclassification of b. gracilis as campylobacter gracilis comb. nov. *International Journal of Systematic and Evolutionary Microbiology*, 45(1):145–152, 1995.

[32] Gregorio Iraola, Ruben Perez, Hugo Naya, Fernando Paolicchi, Eugenia Pastor, Sebastian Valenzuela, Lucía Calleros, Alejandra Velilla, Martín Hernández, and Claudia Morsella. Genomic evidence for the emergence and evolution of pathogenicity and niche preferences in the genus campylobacter. *Genome biology and evolution*, 6(9):2392–2405, 2014.

[33] Jeffrey S McLean, Sarah J Fansler, Paul D Majors, Kathleen McAteer, Lisa Z Allen, Mark E Shirtliff, Renate Lux, and Wenyuan Shi. Identifying low ph active and lactate-utilizing taxa within oral microbiome communities from healthy children using stable isotope probing techniques. *PloS one*, 7(3):e32219, 2012.

[34] Natalia I Chalmers, Kevin Oh, Christopher V Hughes, Nooruddin Pradhan, Eleni Kanasi, Ygal Ehrlich, Floyd E Dewhirst, and Anne CR Tanner. Pulp and plaque microbiotas of children with severe early childhood caries. *Journal of oral microbiology*, 7(1):25951, 2015.

[35] C Freiberg, J Pohlmann, PG Nell, R Endermann, J Schuhmacher, B Newton, M Otteneder, T Lampe, D Häbich, and K Ziegelbauer. Novel bacterial acetyl coenzyme a carboxylase inhibitors with antibiotic efficacy in vivo. *Antimicrobial agents and chemotherapy*, 50(8):2707–2712, 2006.

[36] Russell J Cox, Jennifer S Gibson, and María Belén Mayo Martín. Aspartyl phosphonates and phosphoramidates: The first synthetic inhibitors of bacterial aspartate-semialdehyde dehydrogenase. *ChemBioChem*, 3(9):874–886, 2002.

[37] Sonya Tadrowski, Marcelo M Pedroso, Volker Sieber, James A Larrabee, Luke W Guddat, and Gerhard Schenk. Metal ions play an essential catalytic role in the mechanism of ketol–acid reductoisomerase. *Chemistry–A European Journal*, 22(22):7427–7436, 2016.

[38] Keith Stephenson. Sec-dependent protein translocation across biological membranes: evolutionary conservation of an essential protein transport pathway. *Molecular membrane biology*, 22(1-2):17–28, 2005.

[39] Erwin Chargaff. *The Nucleic Acids*. Elsevier, 2012.

[40] A Maxwell Burroughs and L Aravind. A highly conserved family of domains related to the dna-glycosylase fold helps predict multiple novel pathways for rna modifications. *RNA biology*, 11(4):360–372, 2014.

[41] Johannes Bertsch, Christian Öppinger, Verena Hess, Julian D Langer, and Volker Müller. Heterotrimeric nadh-oxidizing methylenetetrahydrofolate reductase from the acetogenic bacterium acetobacterium woodii. *Journal of bacteriology*, 197(9):1681–1689, 2015.

[42] Milton H Saier. A functional-phylogenetic classification system for transmembrane solute transporters. *Microbiology and molecular biology reviews*, 64(2):354–411, 2000.

[43] MV Gryaznova, SA Solodskikh, AV Panevina, MY Syromyatnikov, Yu D Dvoretskaya, TN Sviridova, ES Popov, and VN Popov. Study of microbiome changes in patients with ulcerative colitis in the central european part of russia. *Heliyon*, 7(3):e06432, 2021.

[44] GM Douglas, R Hansen, CMA Jones, KA Dunn, AM Comeau, JP Bielawski, R Tayler, EM El-Omar, RK Russell, GL Hold, et al. Multi-omics differentially classify disease state and treatment outcome in pediatric crohn's disease. microbiome 6: 13, 2018.

[45] Indrani Mukhopadhya, Richard Hansen, Charlotte E Nicholl, Yazeid A Alhaidan, John M Thomson, Susan H Berry, Craig Pattinson, David A Stead, Richard K Russell, Emad M El-Omar, et al. A comprehensive evaluation of colonic mucosal isolates of sutterella wadsworthensis from inflammatory bowel disease. *PLoS One*, 6(10):e27076, 2011.

[46] Kaisa Hiippala, Veera Kainulainen, Marko Kalliomäki, Perttu Arkkila, and Reetta Satokari. Mucosal prevalence and interactions with the epithelium indicate commensalism of sutterella spp. *Frontiers in microbiology*, 7:1706, 2016.

[47] Elizabeth R Hughes, Maria G Winter, Laice Alves da Silva, Matthew K Muramatsu, Angel G Jimenez, Caroline C Gillis, Luisella Spiga, Rachael B Chanin, Renato L Santos, Wenhan Zhu, et al. Reshaping of bacterial molecular hydrogen metabolism contributes to the outgrowth of commensal e. coli during gut inflammation. *Elife*, 10:e58609, 2021.

[48] Bidong D Nguyen, Miguelangel Cuenca, Johannes Hartl, Ersin Gül, Rebekka Bauer, Susanne Meile, Joel Rüthi, Celine Margot, Laura Heeb, Franziska Besser, et al. Import of aspartate and malate by dcuabc drives h2/fumarate respiration to promote initial salmonella gut-lumen colonization in mice. *Cell host & microbe*, 27(6):922–936, 2020.

[49] Elizabeth R Hughes, Maria G Winter, Breck A Duerkop, Luisella Spiga, Tatiane Furtado de Carvalho, Wenhan Zhu, Caroline C Gillis, Lisa Büttner, Madeline P Smoot, Cassie L Behrendt, et al. Microbial respiration and formate oxidation as metabolic signatures of inflammation-associated dysbiosis. *Cell host & microbe*, 21(2):208–219, 2017.

[50] Kathleen Machiels, Marie Joossens, João Sabino, Vicky De Preter, Ingrid Arijs, Venessa Eeckhaut, Vera Ballet, Karolien Claes, Filip Van Immerseel, Kristin Verbeke, et al. A decrease of the butyrate-producing species roseburia hominis and faecalibacterium prausnitzii defines dysbiosis in patients with ulcerative colitis. *Gut*, 63(8):1275–1283, 2014.

[51] Angela M Patterson, Imke E Mulder, Anthony J Travis, Annaig Lan, Nadine Cerf-Bensussan, Valerie Gaboriau-Routhiau, Karen Garden, Elizabeth Logan, Margaret I Delday, Alistair GP Coutts, et al. Human gut symbiont roseburia hominis promotes and regulates innate immunity. *Frontiers in immunology*, 8:1166, 2017.

[52] Nesrine S El Sayed and Aya S Sayed. Protective effect of methylene blue on tnbs-induced colitis in rats mediated through the modulation of inflammatory and apoptotic signalling pathways. *Archives of toxicology*, 93(10):2927–2942, 2019.

[53] Harry Sokol, Bénédicte Pigneur, Laurie Watterlot, Omar Lakhdari, Luis G Bermúdez-Humarán, Jean-Jacques Gratadoux, Sébastien Blugeon, Chantal Bridonneau, Jean-Pierre Furet, Gérard Corthier, et al. Faecalibacterium prausnitzii is an anti-inflammatory commensal bacterium identified by gut microbiota analysis of crohn disease patients. *Proceedings of the National Academy of Sciences*, 105(43):16731–16736, 2008.
